# Synthesis of dibenzosuberenone-based novel polycyclic π-conjugated dihydropyridazines, pyridazines and pyrroles

**DOI:** 10.3762/bjoc.17.61

**Published:** 2021-03-15

**Authors:** Ramazan Koçak, Arif Daştan

**Affiliations:** 1 Department of Chemistry, Faculty of Sciences, Atatürk University, Erzurum, 25240, Turkey

**Keywords:** dibenzosuberenone, inverse electron-demand Diels–Alder cycloaddition reactions, *p*-quinone methide, polycyclic π-conjugated dihydropyridazines, pyridazines, pyrroles

## Abstract

The synthesis of novel polycyclic π-conjugated dihydropyridazines, pyridazines, and pyrroles was studied. Dihydropyridazine dyes were synthesized by inverse electron-demand Diels–Alder cycloaddition reactions between a dibenzosuberenone and tetrazines that bear various substituents. The pyridazines were synthesized in high yields by oxidation of dihydropyridazine-appended dibenzosuberenones with PIFA or NO. *p*-Quinone derivatives of pyridazines were also obtained by H-shift isomerization following the inverse electron-demand Diels–Alder reaction of tetrazines with *p*-quinone dibenzosuberenone. Then these pyridazines were converted to the corresponding pyrroles by reductive treatment with zinc. It was observed that all the dihydropyridazines obtained gave absorbance and emission at long wavelengths.

## Inroduction

Dibenzosuberone and dibenzosuberenone derivatives are commonly used for the synthesis of biologically active compounds having enzyme inhibition and antiviral activity [1–2], and are found in the structures of many commercially available antidepressant drugs [3–12]. In addition, dibenzosuberenone (**1**) and polyconjugated derivatives exhibit photophysical properties such as photosensitization [13], fluorescence, and aggregation-induced emission (AIE) [14–16].

π-Conjugated polycyclic hydrocarbons (CPHs) containing polycyclic heteroaromatic molecules (PHAs) and aza-polycyclic aromatic hydrocarbons (aza-PAHs) have been attracting considerable attention as they are widespread in natural products, as well as in pharmaceuticals, agrochemicals, and organic materials. Among the π-CPHs, pyridazines and pyrroles have important roles [17–22].

**Figure 1:**
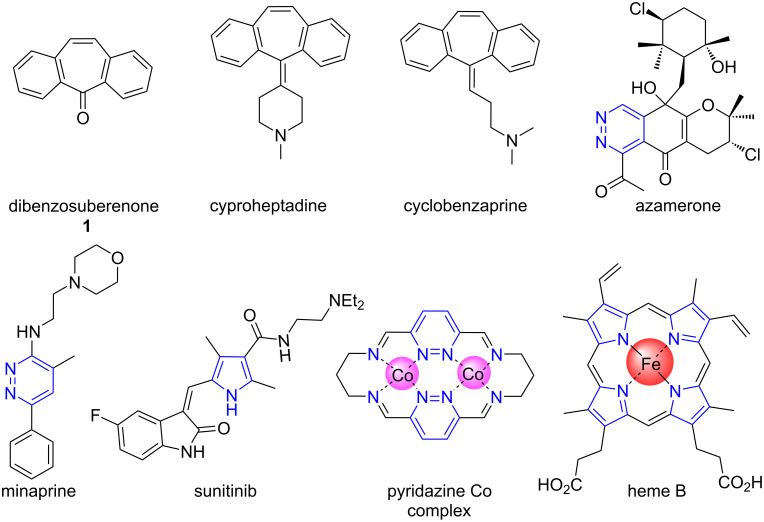
Structures of dibenzosuberenone **1** and pyridazine and pyrrole derivatives.

Although there are very few pyridazine ring-containing compounds isolated from nature (pyridazomycin, pyridazocidin, and azamerone (Figure 1)) [23–25], numerous pyridazine derivatives have been synthesized and used in a wide variety of biochemical and physicochemical applications. Examples of these applications are as drug ingredients [26–31]; for their analgesic, anticancer, antihypertensive, anti-Parkinson, anti-inflammatory, anticonvulsant, vasodilatory, antidiabetic, antitubercular, antifungal, and antibacterial activities [32–38], pH-sensing [39], OLEDs [40], chemiluminescent materials [41–42], metal complexes (Figure 1) [43–46], liquid crystal [47], and self-assembled supramolecular architectures [48].

On the other hand, many natural compounds that contain a pyrrole core, such as bilirubin, hemoglobin, chlorophyll a, and vitamin B12, are very important for life. In addition to being common in natural products and biological systems, the active ingredient of many top-selling drugs such as atorvastatin, sunitinib, and ketorolac, contains a pyrrole unit (Figure 1) [49].

Due to these unique features, the synthesis of new dihydropyridazines, pyridazines, and pyrroles, which have the potential to be used in many applications, is very important. inverse electron-demand Diels–Alder cycloaddition reactions of alkenes with tetrazines are commonly used for the synthesis of dihydropyridazines and pyridazines [50–54].

**Scheme 1:**
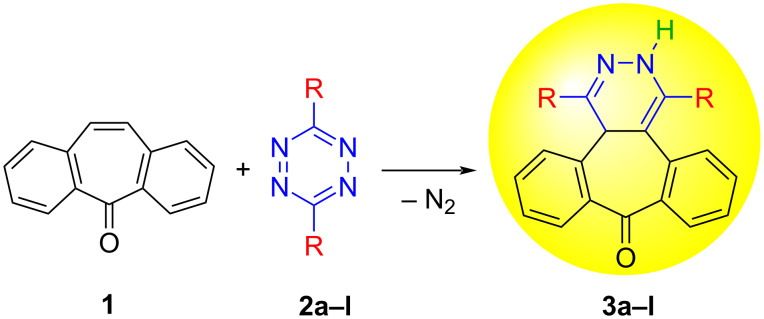
Inverse electron-demand Diels–Alder reactions of dibenzosuberenone (**1**) with tetrazines **2a–l**.

In our previous study, we made a discovery that would form the basis of a new class of dyestuffs with skeletons unlike those of classic organic dyestuffs. In that study, two highly fluorescent dibenzosuberenone-based dihydropyridazine dyes, **3a**,**b**, were synthesized, and it was found that they can be used as a selective and sensitive sensor of fluoride anions (Scheme 1, Table 1) [55]. In another work, we reported the design, synthesis, and structural and photophysical characterization of a new series of 3,7-substituted dihydropyridazine dibenzosuberenone units with electron-withdrawing and electron-donating functional groups [56].

Herein, we report the examined impact of various electron-withdrawing and electron-donating functional groups at the 3- and 6-positions of *s*-tetrazine on inverse electron-demand Diels–Alder cycloaddition reactions with a dibenzosuberenone (**1**) and the photophysical properties of dihydropyridazines. The corresponding pyridazines and pyrroles were obtained from dihydropyridazines. Finally, we investigated the photophysical properties of dihydropyridazines.

## Results and Discussion

### Synthesis

**Figure 2:**
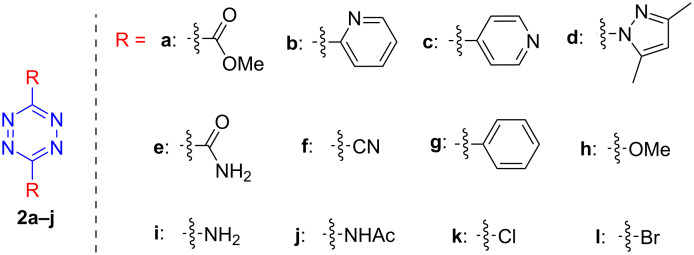
Structures of *s-*tetrazines **2a–l**.

In the first part of the study, we focused on the inverse electron-demand Diels–Alder cycloaddition reactions of dibenzosuberenone (**1**) with *s*-tetrazines **2a–l** (Figure 2), which were synthesized according to the literature procedures (**2j** was synthesized by acetylation of **2i**, while **2b** and **2g** were purchased) [57–63].

When dibenzosuberenone (**1**) and s-tetrazines **2a–j** (1.1 equiv) were dissolved in toluene in a sealed tube and stirred at 100–125 °C for 2–48 h, a sequence of a [4 + 2]-Diels–Alder cycloaddition reaction, a retro Diels–Alder reaction of the resulting adduct, and a final 1,3-prototropic hydrogen shift took place to afford cycloadducts **3a**,**b** [55] and **3c–f** (87–96% yield) (Scheme 1 and Table 1).

**Table 1:** Synthesis of cycloadducts **3a–l**.

Entry	R	Temperature (°C)	Time (h)	Yield of **3** (%)

**1** [55]	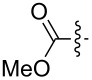 **a**	100	10	95
**2** [55]	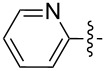 **b**	120	10	96
**3**	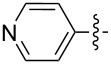 **c**	125	16	96^a^
**4**	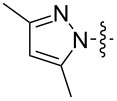 **d**	120	48	87^a^
**5**	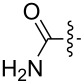 **e**	100	16	95^a^
**6**	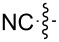 **f**	100	2	87^a^
**7**	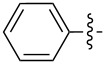 **g**	150	48	–
**8**	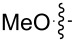 **h**	150	24	–
**9**	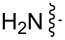 **i**	150	24	–
**10**	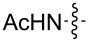 **j**	150	24	–

^a^All reactions were carried using the compounds **1** and **2a–l** (1.1 equivalents) in toluene in a sealed tube (see the Experimental section in Supporting Information File 1 for details).

As shown by the reaction conditions in Table 1, the reactions of tetrazine derivatives with electron-withdrawing groups (EWGs) **2a–f** (4-pyridyl, 3,5-dimethyl-1*H*-pyrazol-1-yl, CONH_2_, CN) resulted in the formation of the target molecules **3a–f** in high yields. However, the reactions of tetrazine derivatives with electron-donating groups (EDGs) **2g–j** (Ph, OMe, NH_2_, NHAc) did not work under the same reaction conditions.

Inverse electron-demand Diels–Alder reactions are cycloadditions between electron-rich dienophiles and electron-poor dienes. EDGs raise the electron density of dienes and, in parallel, raise the LUMO_diene_–HOMO_dienophile_ energy gap, and consequently the reactivity decreases. Although the tetrazine ring is an electron-poor diene, the dibenzosuberenone (**1**) dienophile is not electron-rich enough. Therefore, for such cycloaddition reactions, the tetrazine must be substituted by EWGs to decrease the electron density of the diene. Consequently, our results confirm that EDGs and EWGs play a crucial role in the reactivity.

**Scheme 2:**
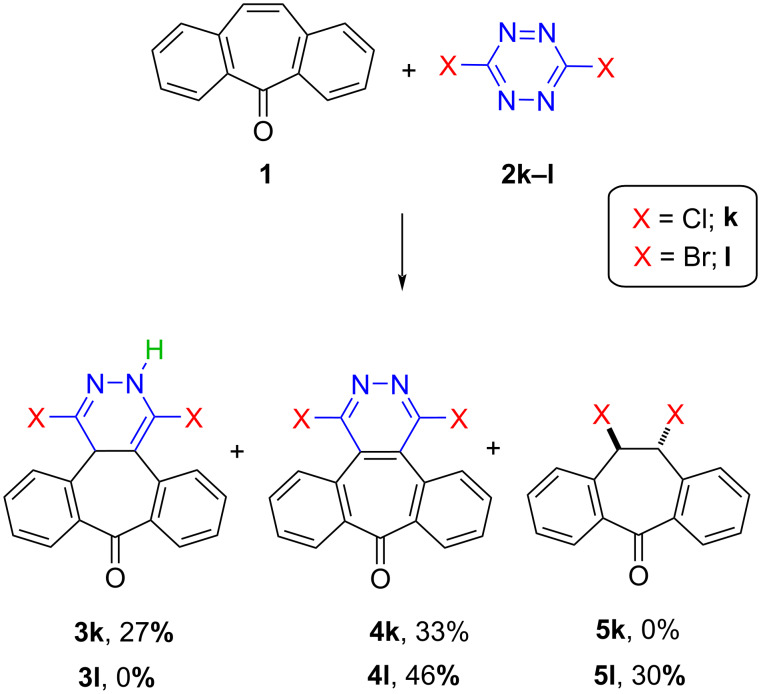
Inverse electron-demand Diels–Alder reactions between dibenzosuberenone **1** and tetrazines **2k**^a^ and **2l**^b^. ^a^5.55 mmol **1**, 3.70 mmol **2k**, 10 mL toluene, 120 ˚C, 48 h. ^b^4.85 mmol **1**, 0.98 mmol **2l**, 100 ˚C, overnight (solvent free).

It should be noted that, unlike the other tetrazine derivatives, dichlorotetrazine (**2k**) afforded the addition product **3k** and the oxidation product **4k** as a result of its reaction with dibenzosuberenone (**1**). When dibromotetrazine (**2l**) was used, on the other hand, the oxidation product **4l** and an unexpected product, dibromobenzonorbornadiene **5l**, were formed (Scheme 2).

**Scheme 3:**
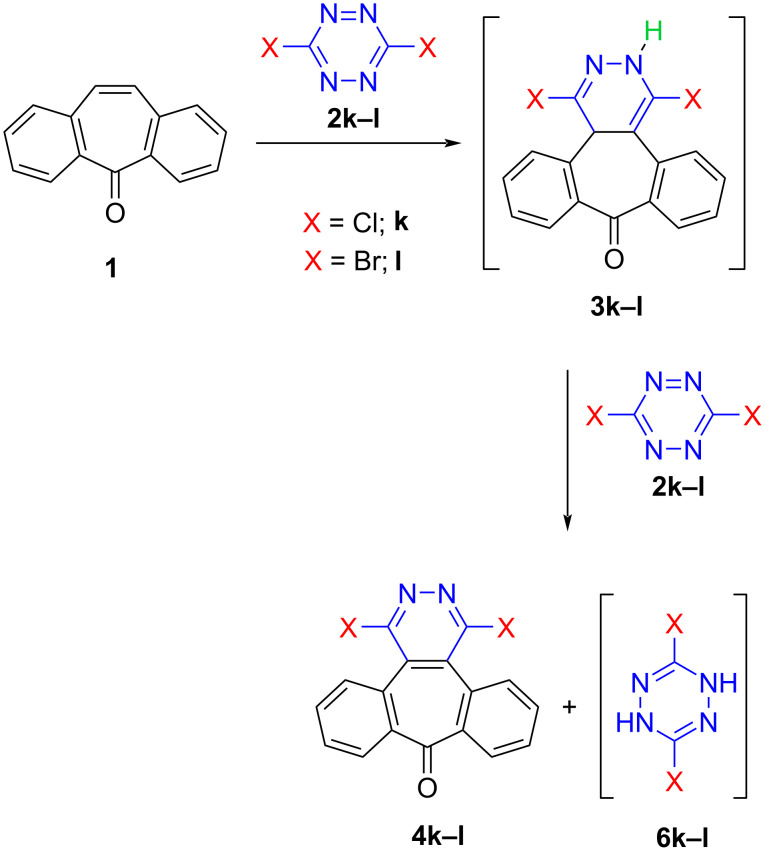
Proposed reaction mechanism for the formation of dibenzosuberenone derivatives **3** and **4**.

The formation of the oxidized products **4k** and **4l** in these reactions was probably due to the properties of tetrazines because they are known to act, in some cases, as oxidizing agents in Diels–Alder reactions, depending on the structure of the diene and the reaction conditions [64]. A proposed reaction mechanism for the formation of the dibenzosuberenone derivatives **3** and **4** is illustrated in Scheme 3. Although **3k** was isolated, **3l** could not be detected in the reaction mixture due to its complete conversion to **4l**, revealing its ease of oxidation compared to the chloro derivative **3k**. In addition, dihydrotetrazines **6k** and **6l** could not be isolated, as probably compound **6k** decomposed completely in the reaction medium and **6l** decomposed following a bromine transfer to dibenzosuberenone (**1**, Scheme 3).

**Scheme 4:**
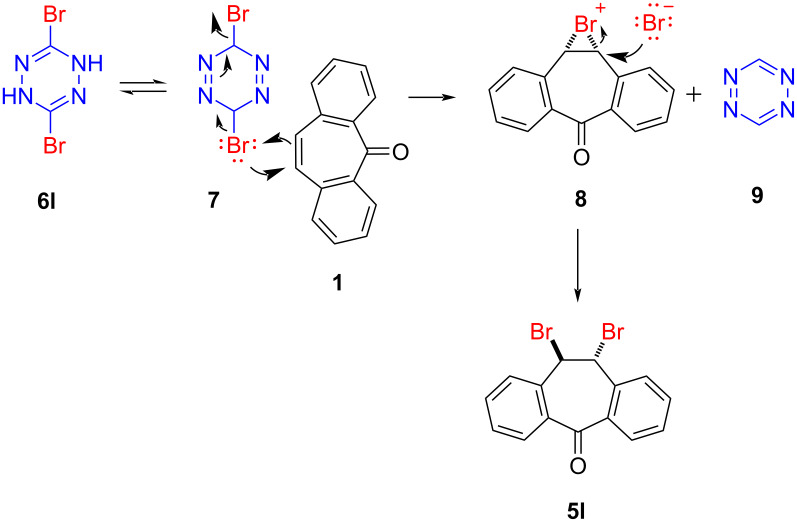
Proposed mechanism for the formation of **5l**.

The formation of **5l** was also an unexpected result because there has been no precedent reported to date, in which a tetrazine acts as a halogen source in the halogenation of a double bond. Therefore, the formation of **5l** constitutes the first example of this unusual behavior. In the proposed mechanism illustrated in Scheme 4, tetrazine **6l** first tautomerizes into **7**, from which dibenzosuberenone (**1**) receives bromine to give **5l** via bromonium **8**. As tetrazine **9** was unstable under the current reaction conditions, it decomposed and could not be observed [65].

**Scheme 5:**
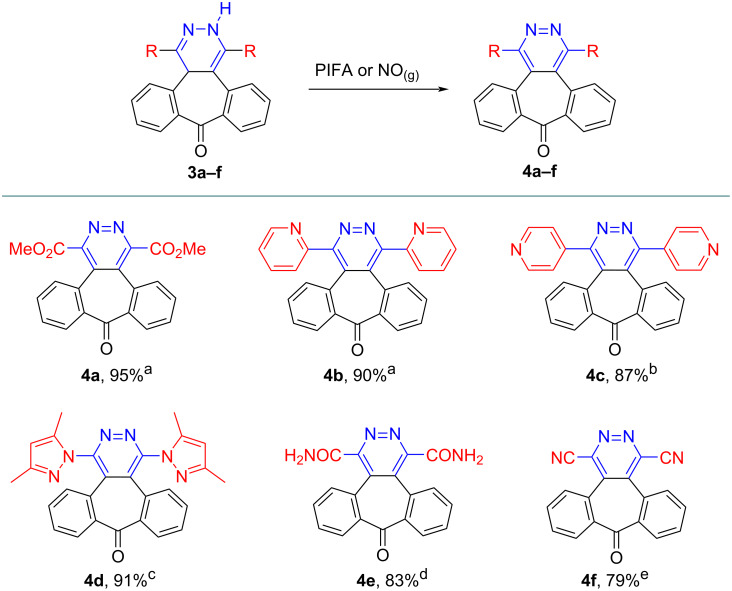
Oxidation of dihydropyridazines **3a–f**. All reactions were carried in CH_2_Cl_2_ at room temperature (**4e:** 0 °C). ^a^1.0 equivalent PIFA, 1 h. ^b^1.5 equivalents PIFA, overnight. ^c^1.2 equivalents PIFA, overnight. ^d^Nitrous gases were bubbled through a solution of **3e** for 1 h. ^e^1.0 equivalent PIFA, overnight.

In the second part of the study, dihydropyridazines **3a–f** were oxidized to pyridazines. In contrast to dihydropyridazineamide **3e**, the reaction of dihydropyridazines **3a–d** and **3f** with PIFA ([bis(trifluoroacetoxy)iodo]benzene) afforded the corresponding pyridazine derivatives **4a–d** and **4f** in good yields (79–95%). As a result of the reaction of PIFA with dihydropyridazine **3e**, the intended pyridazine compound could not be obtained. Alternatively, nitrogen monoxide (NO) gas was used as oxidizer and pyridazineamide **4e** was obtained in high yield (83%, Scheme 5).

In another important part of this study, pyridazines were converted into the corresponding pyrroles via a ring contraction under reductive conditions in presence of zinc dust in acetic acid according to the Boger procedure, which is a highly reliable synthetic approach [66–68]. For pyrrole conversions, methoxycarbonyl- and 2-pyridylpyridazine derivatives **4a** and **4b** were used.

**Scheme 6:**
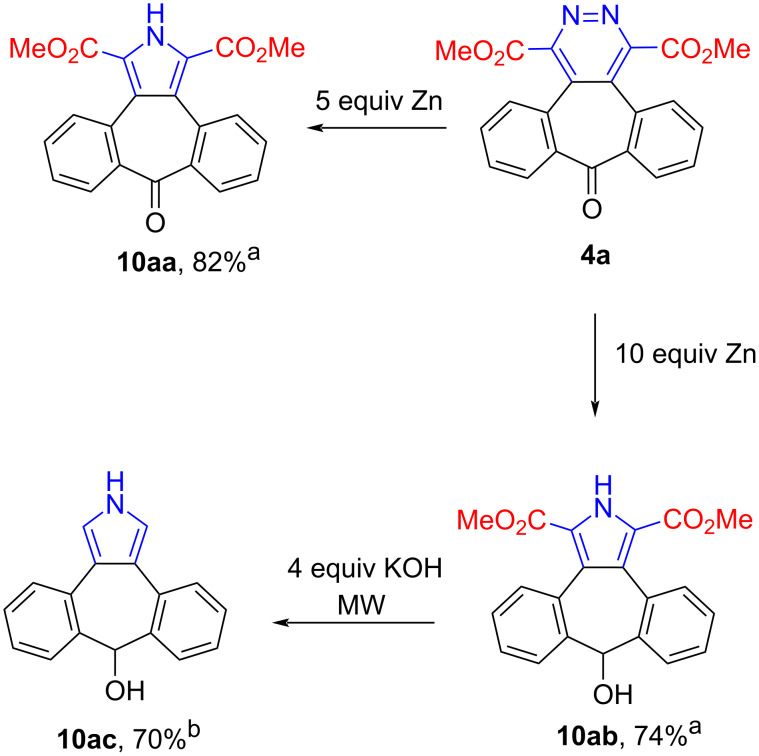
Synthesis of pyrrole **10a**. ^a^1.34 mmol **4a**, Zinc (for **10aa**: 6.68 mmol, for **10ab**: 13.36 mmol), 10 mL glacial acetic acid, room temperature, overnight. ^b^0.55 mmol **10ab**, 2.20 mmol KOH, 5 mL THF/CH_3_OH/H_2_O (2:2:1) solvent mixture, 150 °C, 200 W, 2 h.

When methoxycarbonylpyridazine **4a** reacted with 5 equivalents of Zn in acetic acid at room temperature overnight the corresponding pyrrole **10aa** was obtained. When using 10 equivalents of Zn instead of 5 equivalents, hydroxypyrrole **10ab** was formed, in which the carbonyl group was also reduced to the hydroxy group. Then unsubstituted pyrrole **10ac** was synthesized by the reaction of pyrrole **10ab** with 4 equivalents of KOH under microwave irradiation (Scheme 6).

**Scheme 7:**
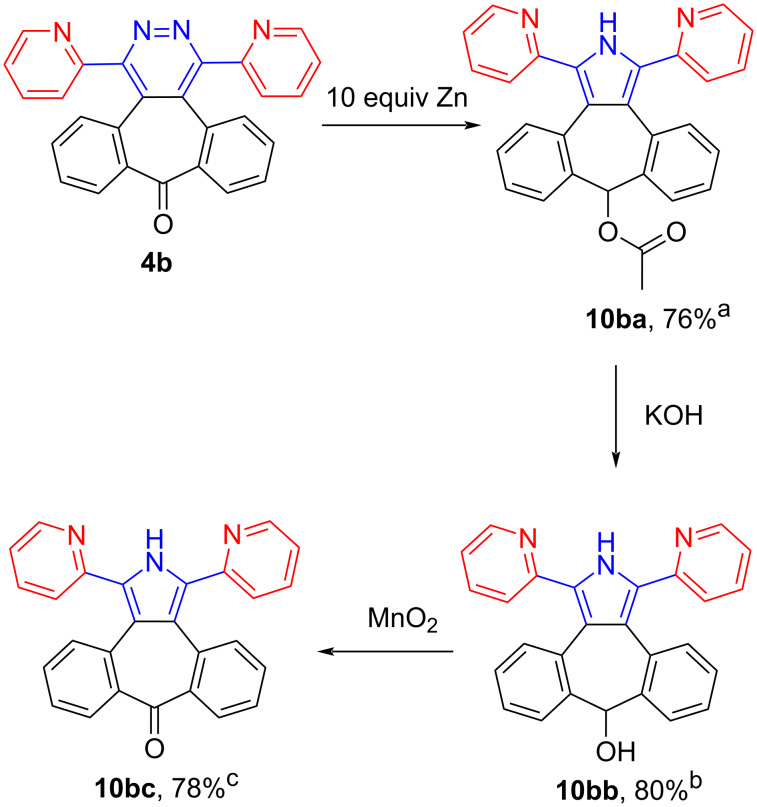
Synthesis of pyrrole **10b**. ^a^1.21 mmol **4b**, 12.10 mmol Zinc, 118 °C, 2 h. ^b^1.13 mmol **10ba**, 1.69 mmol KOH, 10 mL H_2_O/EtOH (1:3), room temperature, 4 h. ^c^1.25 mmol **10bb**, 12.45 mmol MnO_2_, 10 mL CH_2_Cl_2_, room temperature, 3 h.

The reaction of 2-pyridylpyridazine **4b** with Zn did not work at room temperature. Under reflux conditions compound **10ba** was obtained, which contained the corresponding pyrrole and acetate structures. The acetate is formed by reducing the carbonyl group to alcohol and then reacting this alcohol with acetic acid. After hydrolysis of the acetate group with sodium hydroxide, alcohol derivate **10bb** was formed. Oxidation of alcohol **10bb** with MnO_2_ led to the formation of the corresponding ketone **10bc**. Eventually, pyrrole derivative **10bc**, having a carbonyl group, was obtained by these reactions (Scheme 7).

**Scheme 8:**
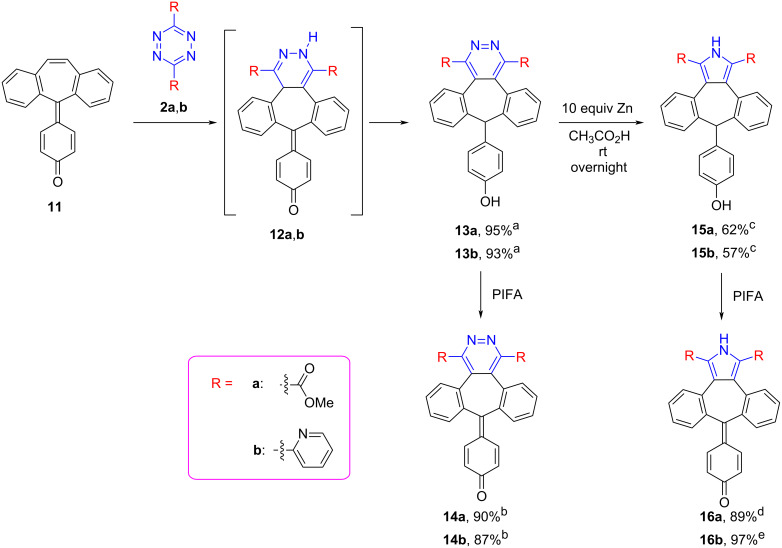
Synthesis of *p*-quinone methides **13–16. **^a^1.77 mmol **11**, 1.77 mmol **2**, 5 mL toluene, 80 °C (**13a**: overnight, **13b**: 3 days). ^b^1.11 mmol **13,** 1.33 mmol PIFA, 20 mL CH_2_Cl_2_, room temperature, overnight. ^c^1.1 mmol **13**, 11.1 mmol Zinc, 10 mL glacial acetic acid, room temperature, overnight. ^d^1.02 mmol **15a**, 1.22 mmol PIFA, 20 mL CH_2_Cl_2_, at room temperature, overnight. ^e^1.05 mmol **15b**, 1.05 mmol DDQ, 20 mL CH_2_Cl_2_, room temperature, 30 min.

In order to increase the conjugation of dibenzosuberenone **1** for the photophysical aspect, the *p*-quinone methide derivative of dibenzosuberenone **11** was synthesized according to the method in the literature [69]. The products expected from the reaction of *p*-quinone methide **11** with tetrazines **2a**,**b** are dihydropyridazines **12a**,**b**, but this molecule could not be obtained. Instead, surprisingly, products **13a**,**b** were obtained, of which the dihydropyridazine part was oxidized to pyridazine and the *p*-quinone methides part was reduced to phenol. After the phenolic part of **13a**,**b** was oxidized to *p*-quinone methides with PIFA, **14a** and **14b** were synthesized in 87% and 91% yields, respectively. Moreover, by submitting **13a**,**b** to reductive conditions in presence of Zn, the pyridazine part of **13a**,**b** was converted to pyrrole **15a**,**b**. Finally, the phenolic parts of **15a,b** were oxidized to *p*-quinone methides **16a**,**b** with PIFA in excellent yield (89–97%, Scheme 8).

**Scheme 9:**
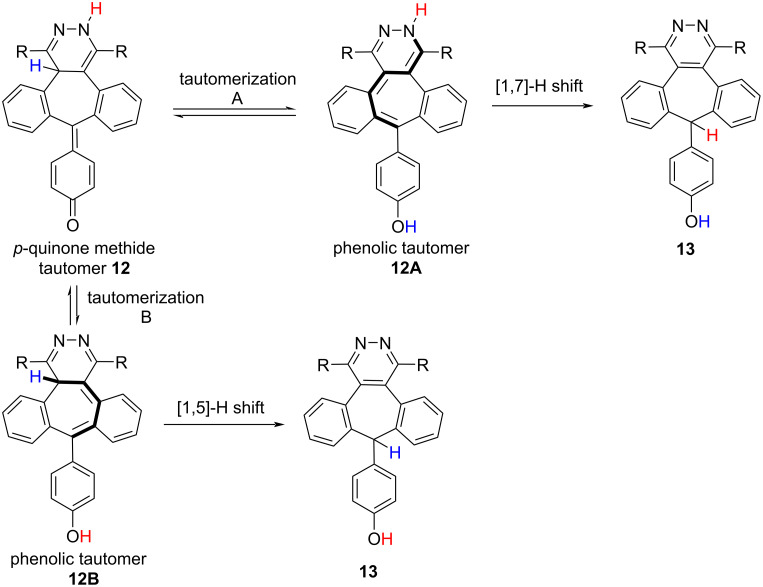
Proposed mechanism for the formation of **13**.

For the formation of unexpected compound **13**, we propose two different mechanisms. First, following the formation of phenolic tautomer **12A** by tautomerization A with a [1,7]-H shift **13** would be formed. Alternatively, in the second mechanism, **13** is thought to occur by tautomerization B and a [1,5]-H shift (Scheme 9).

### Photophysical properties

**Figure 3:**
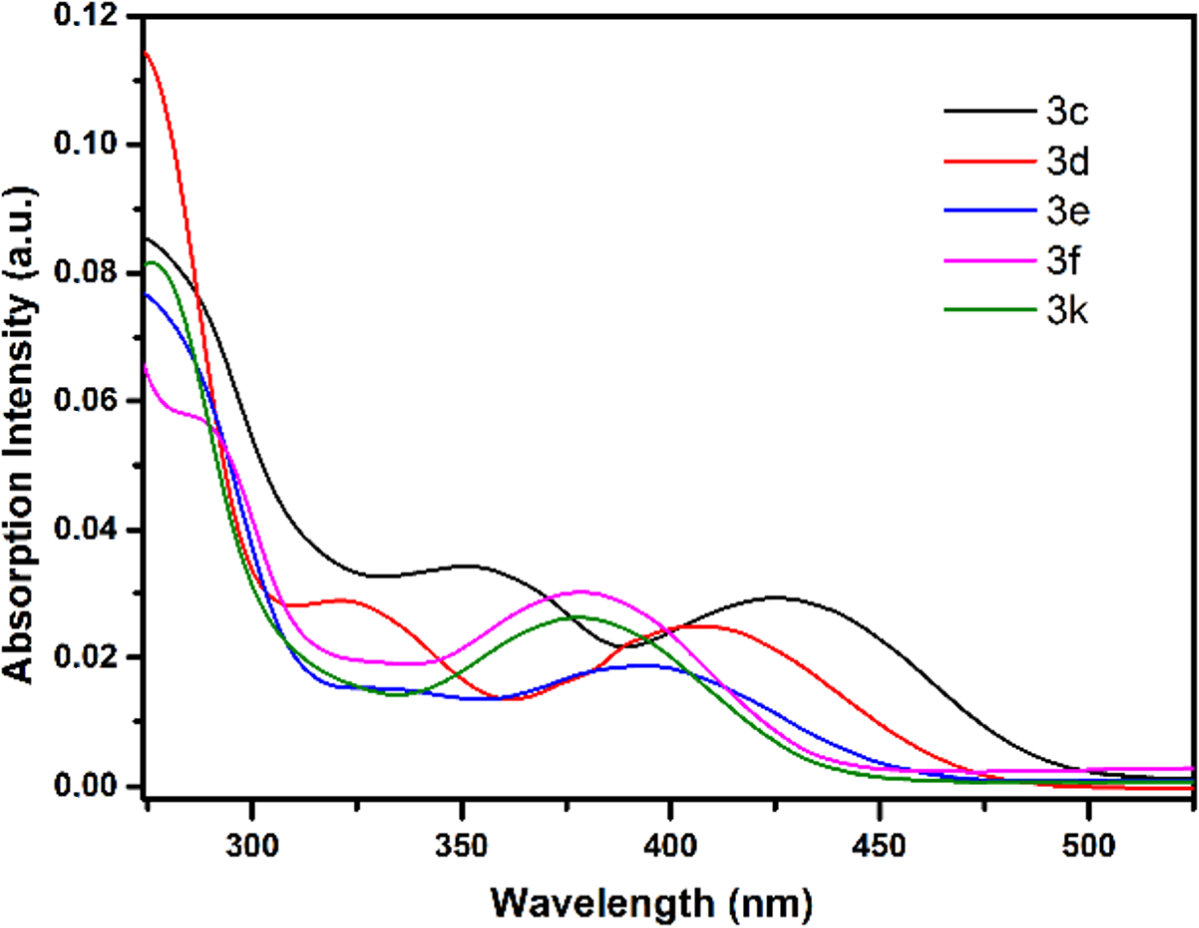
UV–vis spectra of **3c–f** and **3k** in CH_3_CN at rt (*c* = 5 μM).

**Figure 4:**
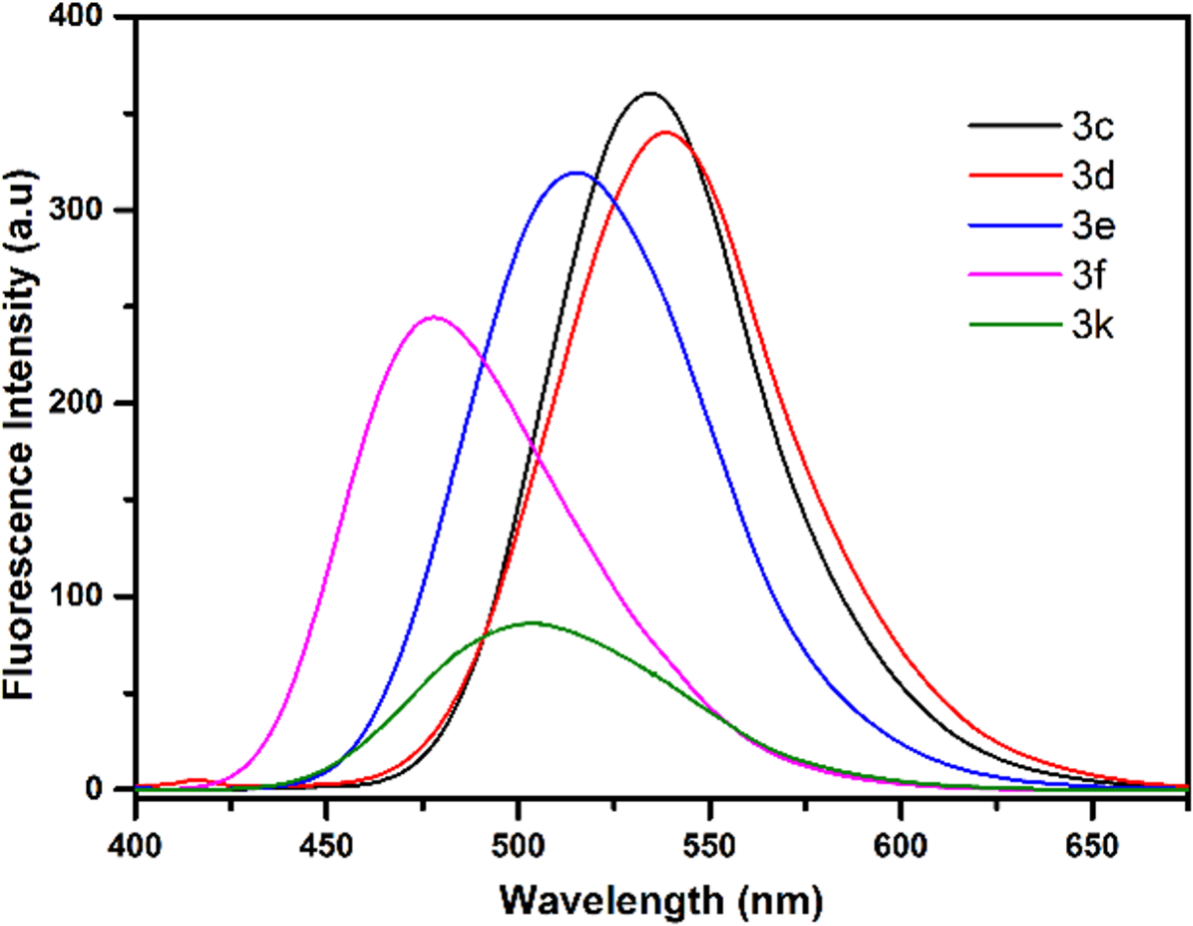
Fluorescence spectra of **3c–f** and **3k** in CH_3_CN at rt (*c* = 5 μM).

**Figure 5:**
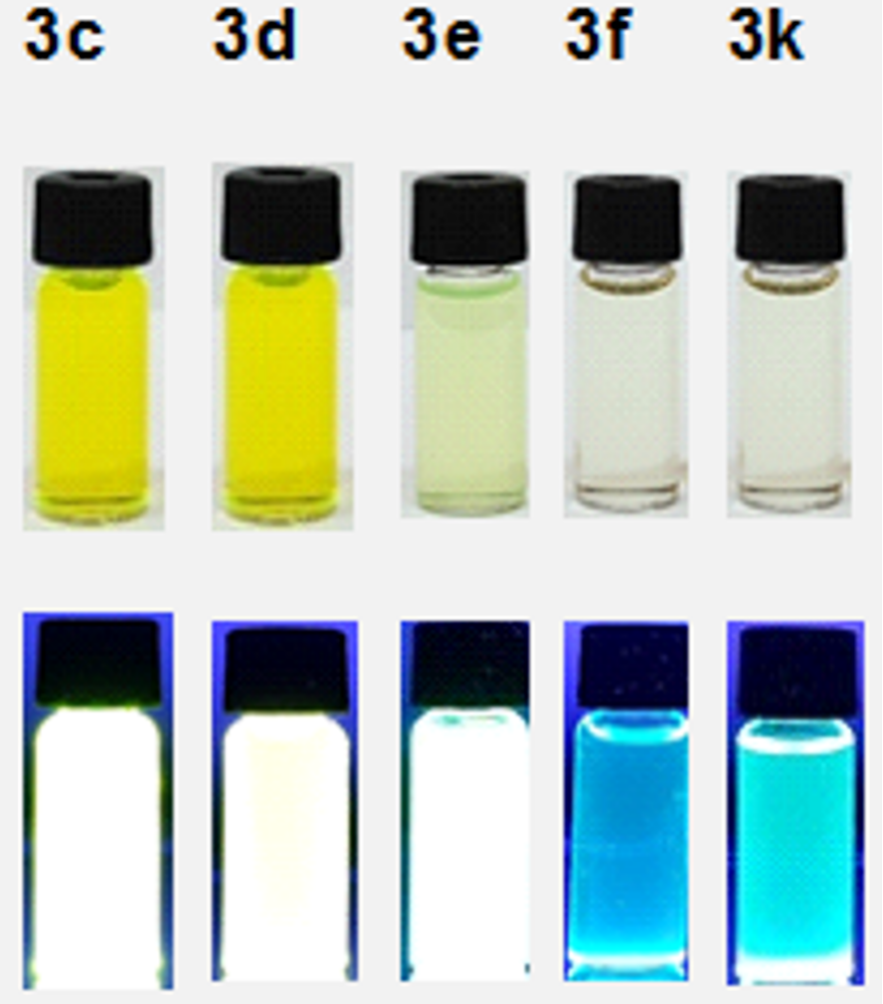
Ambient (top) and fluorescence (bottom, under 365 nm UV light) images of **3c–f** and **3k** in CH_3_CN.

In our previous work, we examined the photophysical and fluoride sensing properties of dihydropyridazine fluorescent dyes **3a**,**b** in detail. In the present study, the effects of functional groups with different conjugations on maximum absorbance (λ_max_, _abs_) and emission (λ_max_, _ems_), and wavelengths of dihydropyridazines **3c–f** and **3k** were investigated. Compounds **3c**,**d**, with high conjugation, show the highest absorption and emission maxima values and compounds **3f** and **3k**, with low conjugation, show the lowest absorption and emission maxima values (Figure 3, Figure 4, and Figure 5). All these molecules (**3c–f** and **3k**) have Stokes shifts greater than 100 nm. The fluorescence quantum yields of **3c–f** and **3k** were calculated by comparison with a well-known reference, quinine sulfate, in 0.5 M H_2_SO_4_ solution (Ф_F_ = 0.546) as the standard dye (Table 2). When the UV–vis and fluorescence spectra of pyridazines and pyrroles were examined, it was seen that generally, they did not have effective absorbance or emission intensity. Although all π-conjugated pyridazines and pyrroles known in the literature did not show high emission, along with other photophysical properties the excellent coordination ability and especially their importance in biological systems always make these compounds valuable.

**Table 2:** Some photophysical properties of cycloadducts **3c–3f** and **3k**.

Compound	λ_ems_/nm^a^ ( λ_exc_/nm)	λ_abs_/nm ^[a]^	Stokes shift (nm)	Quantum yields (Ф_F_)

**3c**	534 (400)	427	107	0.78
**3d**	539 (375)	408	131	0.60
**3e**	515 (360)	393	122	0.53
**3f**	487 (350)	378	109	0.28
**3k**	503 (350)	378	125	0.16

^a^*c* = 5 μM (CH_3_CN).

## Conclusion

Novel polycyclic π-conjugated dibenzosuberenone-based dihydropyridazine dyes were synthesized by inverse electron-demand Diels–Alder cycloaddition reactions between dibenzosuberenone (**1**) and tetrazines bearing various substituents. These products showed long absorption wavelengths and emission bands, large Stokes shifts, and good fluorescence quantum yields. The dihydropyridazines were oxidized into pyridazines and then converted to pyrroles. Moreover, *p*-quinone methide derivatives of dibenzosuberenone-based pyridazines and pyrroles were synthesized. We continue intensively to perform various photochemical and biochemical studies on these compounds, which have the potential to be used in chemosensors, light harvesting, organic optoelectronics, metal coordination complexes and other photophysical applications and in biological systems.

## Supporting Information

File 1 Experimental procedures, copies of ^1^H NMR, ^13^C NMR, and HRMS(Q-TOF) spectra.
