# Supplementary material for: Synthesis of dibenzosuberenone-based novel polycyclic π-conjugated dihydropyridazines, pyridazines and pyrroles
Source: Beilstein J Org Chem. 2021 Mar 15;17:719–29. doi: 10.3762/bjoc.17.61 (PMC7991620; doi:10.3762/bjoc.17.61)
Supplement: File 1 — Experimental procedures, copies of 1H NMR, 13C NMR, and HRMS(Q-TOF) spectra. [file Beilstein_J_Org_Chem-17-719-s001.pdf]

BEILSTEIN JOURNAL OF ORGANIC CHEMISTRY

Supporting Information

for

Synthesis of dibenzosuberenone-based novel polycyclic π-conjugated dihydropyridazines, pyridazines and pyrroles

Ramazan Koçak and Arif Daştan

Beilstein J. Org. Chem. **2021,** *17,* 719–729. doi:10.3762/bjoc.17.61

Experimental procedures, copies of ^{1}H NMR, ^{13}C NMR, and HRMS(Q-TOF) spectra

License and Terms: This is a supporting information file under the terms of the Creative Commons Attribution License (https://creativecommons.org/licenses/by/4.0). Please note that the reuse, redistribution and reproduction in particular requires that the author(s) and source are credited and that individual graphics may be subject to special legal provisions.
The license is subject to the *Beilstein Journal of Organic Chemistry* terms and conditions: (https://www.beilstein-journals.org/bjoc/terms)

Experimental

General

The one and two dimensional ^{1}H and ^{13}C NMR spectra were recorded on a Varian-400 or a Bruker-400 spectrometer in $CDCl_3$, CD_3CN, and DMSO-d_6 using tetramethylsilane as the internal reference. All spectra were recorded at 25 °C and coupling constants (*J* values) are given in Hz. Chemical shifts are given in parts per million (ppm). Abbreviations used to define the multiplicities are as follows: s = singlet; d = doublet; dd = doublet of doublets; m = multiplet. Mass spectra were recorded on an Agilent Technologies 6530 Accurate-Mass Q-TOF–LC/MS spectrometer. Absorption spectrometry was performed using a Perkin Elmer Lambda 35 spectrophotometer. Steady-state fluorescence measurements were conducted using a Shimadzu RF-5301PC spectrofluorometer. Stock solutions of **3c-f** and **3k** (1×10^{-3} M) were prepared in acetonitrile. The concentration of **3c–f** and **3k** for all spectroscopy measurement was kept at 5 µM by diluting the stock solution. The reactions under microwave irradiation were carried out in a 300W CEM Discover microwave reactor.

General procedure A: invers-Diels–Alder cycloaddition reactions between dibenzosuberenone (1) and tetrazine derivatives

Dibenzosuberenone (**1**) or *p*-quinone metide **11** and tetrazine **2** were dissolved in toluene in an ACE pressure tube. The reaction mixture was head and stirred. At the end of the reaction, the red color of tetrazine disappeared. The mixture was cooled to rt and some of the solvent was evaporated under reduced pressure. The precipitated product was purified by crystallization or silica gel column chromatography to give dihydropyridazines.

General procedure B: Oxidation with PIFA

To a solution of **3**, **13** or **15** in 20 mL of CH_2Cl_2 was added PIFA and this was stirred at room temperature for 1 h to overnight. The solvent was evaporated, and the crude product was purified by column chromatography and crystallization to give corresponding oxidized products **4**, **14** and **16**.

General procedure C: Ring contraction of pyridazines to pyrroles

Zinc dust was added to a solution of pyridazine **4a**,**b** and **13a**,**b** in 10 mL of glacial acetic acid and the reaction was stirred. At the end of the reaction, mixture was filtered through Celite®, the filtrate was neutralized with the addition of saturated aqueous $NaHCO_3$, and extracted with EtOAc (2 × 25). The combined organic layer was dried over Na_2SO_4 and evaporated under vacuo. The obtained solid was purified by column

chromatography and crystallization to give related pyrrole (**10aa**, **10ab**, **10ba** and **15a**,**b**).

_1_,4-Di(pyridin-4-yl)-2,4a-dihydro-9_H_-dibenzo[3,4:6,7]cyclohepta[1,2-_d_]pyridazin-9-one (3c)

The reaction was performed according to the general procedure A with **1** (1.0 g, 4.85 mmol) and tetrazine **2c** (575 mg, 2.43 mmol) in 3 mL toluene at 125 °C for 16 h. The precipitated product was filtrated, washed with Et_2O and recrystallized from *n*-hexane/CH_2Cl_2 (1:9) to give **3c** as yellow crystals (yield 1.93 g, 96%). Mp: 294-296 ˚C. ^{1}H-NMR (400 M Hz, $CDCl_3$): δ=8.60 (d, *J* = 5.8 Hz, 2H), 8.57-8.52 (m, 3H), 8.28 (bs, NH, 1H), 7.98 (dd, *J* = 7.0 Hz, *J* = 1.4 Hz, 1H), 7.61 (d, *J* = 5.9 Hz, 2H), 7.44-7.33 (m, 3H), 7.20-7.13 (m, 3H), 6.86 (d, *J* = 7.5 Hz, 2H), 5.15 (s, 1H). ^{13}C-NMR (100 M Hz, $CDCl_3$): δ=193.4, 150.8, 150.4, 142.9, 141.9, 138.41, 137.37, 137.3, 137.1, 135.6, 132.7, 132.5, 132.2, 132.0, 131.9, 131.6, 127.71, 127.65, 124.0, 123.95, 120.2, 106.1, 40.9. HRMS (Q-TOF): *m/z* $[M + H]^+$ calcd for $C_{27}H_{19}N_4O$: 415.1559, found: 415.1549.

1,4-Bis(3,5-dimethyl-1_H_-pyrazol-1-yl)-2,4a-dihydro-9_H_-dibenzo[3,4:6,7]cyclohepta[1,2-_d_]pyridazin-9-one (3d).

The reaction was performed according to the general procedure A with **1** (1.14 g, 5.55 mmol) and tetrazine **2d** (1.0 g, 3.70 mmol) in 2 mL toluene at 120 °C for 48 h. The product was purified by column chromatography (SiO_2, *n*-hexane/EtOAc (4:1) to give **3d** as an orange solid (yield 1.44 g, 87%). Mp: 228-230 ˚C. ^{1}H-NMR (400 M Hz, $CDCl_3$): δ=8.46 (d, *J* = 7.9 Hz, 1H), 7.84 (bs, NH, 1H), 7.82 (d, *J* = 7.9 Hz, 1H), 7.43 (t, *J* = 7.5 Hz, 1H), 7.34 (d, *J* = 7.4 Hz, 1H), 7.30 (d, *J* = 7.4 Hz, 1H), 7.28-7.23 (m, 1H), 7.00 (d, *J* = 7.7 Hz, 1H), 6.68 (d, *J* = 7.7 Hz, 1H), 5.90 (s, 1H), 5.75 (s, 1H), 5.72 (s, 1H), 2.61 (s, 3H), 2.27 (s, 3H), 2.08 (s, 3H), 1.12 (s, 3H). ^{13}C-NMR (100 M Hz, $CDCl_3$): δ=194.2, 151.3, 149.7, 142.5, 141.8, 138.4, 137.6, 137.5, 137,4, 136.2, 132.9, 132.2, 131.6, 131.3, 130.8, 129.2, 127.2, 127.0, 123.4, 109.4, 107.8, 100.5, 42.6, 14.8, 13.8, 13.7, 10.4. HRMS (Q-TOF): *m/z* $[M + H]^+$ calcd for $C_{27}H_{25}N_6O$: 449.2090, found: 449.2085.

9-Oxo-4a,9-dihydro-2_H_-dibenzo[3,4:6,7]cyclohepta[1,2-_d_]pyridazine-1,4-dicarboxamide (3e)

The reaction was performed according to the general procedure A with **1** (1.0 g, 4.85 mmol) and tetrazine **2e** (163 mg, 0.97 mmol) at 100 °C for overnight (solvent free). The product was filtered, washed with Et_2O and recrystallized from *n*-hexane/CH_2Cl_2 (1:9) to give **3e** as green crystals (yield 292 mg, 95%). Mp: 282-283 ˚C decomposition. ^{1}H-NMR (400 M Hz, DMSO-d_6): δ= 10.79 (bs, 1H, NH), 8.35 (d, *J* = 7.8 Hz, 1H), 7.77 (d, *J* = 7.8 Hz, 1H), 7.69-7.63 (m, 2H), 7.60 (s, 1H), 7.58-7.45 (m, 4H), 7.38 (s, 1H), 7.35 (d, *J* = 7.6 Hz, 1H), 6.67 (d, *J* = 7.6 Hz, 1H), 4.99 (s, 1H). ^{13}C-NMR (100 M Hz, DMSO-d_6): δ= 192.8, 165.5, 164.7, 138.6, 138.4, 138.1, 136.2, 132.5, 132.2, 131.2, 131.0, 130.4, 130.0, 129.7, 127.5, 126.7, 123.2, 105.2, 36.6. HRMS (Q-TOF): *m/z* $[M + H]^+$ calcd for $C_{19}H_{15}N_4O_3$: 347.1144, found: 347.1137.

9-Oxo-4a,9-dihydro-2*H*-dibenzo[3,4:6,7]cyclohepta[1,2-*d*]pyridazine-1,4-dicarbonitrile (3f)

The reaction was performed according to the general procedure A with **1** (500 mg, 2.42 mmol) and tetrazine **2f** (160 mg, 1.21 mmol) in 5 mL toluene at 100 °C for 2 h. The precipitated product was filtrated, washed with Et_2O and recrystallized from *n*-hexane/CH_2Cl_2 (1:9) to give **3f** as agreen crystals (yield 1.31g, 87%). Mp: 245-246 ˚C. ^{1}H-NMR (400 M Hz, $CDCl_3$): δ= 8.53 (dd, *J* = 7.1 Hz, *J* = 1.5 Hz, 1H), 8.39 (bs, 1H, NH), 7.88 (d, *J* = 7.8 Hz, 1H), 7.84 (d, *J* = 7.1 Hz, 1H), 7.73-7.64 (m, 2H), 7.58 (t, *J* = 7.5 Hz, 1H), 7.47 (t, *J* = 7.5 Hz, 1H), 6.99 (t, *J* = 7.6 Hz, 1H), 4.94 (s, 1H).^{13}C-NMR (100 M Hz, $CDCl_3$): δ= 192.1, 138.4, 137.2, 133.9, 133.23, 133.18, 132.9, 132.5, 132.3, 131.2, 129.3, 129.0, 123.6, 122.45, 115.4, 114.5, 112.6, 107.8, 41.9. HRMS (Q-TOF): *m/z* $[M + H]^+$ calcd for $C_{19}H_{11}N_4O$: 311.0933, found: 311.0931.

Reaction of Dibenzosuberenone (1) with 3,6-dichloro-1,2,4,5-tetrazine (2k).

The reaction was performed according to the general procedure A with **1** (1.14 g, 5.55 mmol) and tetrazine **2k** (0.56 g, 3.70 mmol) in 10 mL toluene at 120 ˚C for 48 h. The crude reaction products was purified by column chromatography on silica gel (10% EtOAc/*n*-hexane).

1. Fraction: **1,4-Dichloro-2,4a-dihydro-9*H*-dibenzo[3,4:6,7]cyclohepta[1,2-*d*]pyridazin-9-one (3k).** Yellow solid (yield 164 mg, 27%). Mp: 223-224 ˚C. ^{1}H-NMR (400 M Hz, $CDCl_3$): δ=8.49 (dd, *J* = 8.0 Hz, *J* = 1.5 Hz, 1H), 7.87 (dd, *J* = 7.6 Hz, *J* = 1.3 Hz, 1H), 7.83 (dd, *J* = 7.7 Hz, *J* = 1.0 Hz, 1H), 7.57 (dt, *J* = 7.6 Hz, *J* = 1.5 Hz, 1H), 7.53 (dt, *J* = 7.7 Hz, *J* = 1.3 Hz, 1H), 7.48 (dt, *J* = 8.0 Hz, *J* = 1.3 Hz, 1H), 7.41 (s, 1H, NH), 7.40 (dt, *J* = 7.6 Hz, *J* = 1.0 Hz, 1H), 7.09 (d, *J* = 7.6 Hz, 1H), 4.90 (s, 1H). ^{13}C-NMR (100 M Hz, $CDCl_3$): δ=192.9, 138.4, 136.3, 136.0, 135.5, 134.4, 132.7, 132.5, 131.9, 131.8, 130.2, 128.1, 127.9, 124.7, 123.1, 102.8, 50.0. HRMS (Q-TOF): *m/z* $[M + H]^+$ calcd for $C_{17}H_{11}Cl_2N_2O$: 329.0248, found: 329.0248.

2. Fraction: **1,4-Dichloro-9*H*-dibenzo[3,4:6,7]cyclohepta[1,2-*d*]pyridazin-9-one (4k).** Colorless solid (yield 199 mg, 33%). Mp: 257-258 ˚C. ^{1}H-NMR (400 M Hz, $CDCl_3$): δ=7.96-7.90 (m, 2H), 7.63-7.56 (m, 6H). ^{13}C-NMR (100 M Hz, $CDCl_3$): δ=196.1, 155.8, 146.2, 135.9, 131.42, 131.39, 130.0, 127.3, 126.0. HRMS (Q-TOF): *m/z* $[M + H]^+$ calcd for $C_{17}H_9Cl_2N_2O$: 327,0092, found: 327,0088.

Reaction of dibenzosuberenone (1) with 3,6-dibromo-1,2,4,5-tetrazine (3l).

The reaction was performed according to the general procedure A with **1** (1.0 g, 4.85 mmol) and tetrazine **2l** (235 mg, 0.98 mmol) at 100 °C for overnight (solvent free). The crude reaction products was purified by recrystallization from CH_2Cl_2/*n*-Hexane (9:1). Pyridazine **4l** was obtained by first crystallization. Then recrystallization of the residue afforded diboromo **5l**.

1. Fraction: **1,4-Dibromo-9*H*-dibenzo[3,4:6,7]cyclohepta[1,2-*d*]pyridazin-9-one (4l).** Colorless solid (yield 186 mg, 46%). Mp: 300-301 ˚C. ^{1}H-NMR (400 M Hz, $CDCl_3$): δ=7.98-7.92 (m, 2H), 7.61-7.53 (m, 6H). ^{13}C-NMR (100 M Hz, $CDCl_3$): δ=196.0, 149.8, 146.1, 137.4, 131.7, 131.4, 129.9, 128.8, 125.8. HRMS (Q-TOF): *m/z* $[M + H]^+$ calcd for $C_{17}H_9Br_2N_2O$: 416.9061, found: 416.9045.

2. Fraction: **10,11-ibromo-10,11-dihydro-5*H*-dibenzo[*a*,*d*][7]annulen-5-one (5l).** Colorless solid (yield 107 mg, 30%). Mp: 204-206 ˚C. ^{1}H-NMR (400 M Hz, $CDCl_3$): δ= 8.09 (dd, *J* = 7.9 Hz, *J* = 1.5 Hz, 2H), 7.57 (dt, *J* = 7.3 Hz, *J* = 1.5 Hz, 2H), 7.50 (dt, *J* = 7.9 Hz, *J* = 1.5 Hz, 2H), 7.41 (d6, *J* = 7.3 Hz, *J* = 1.5 Hz, 2H), 5.80 (s, 2H). ^{13}C-NMR (100 M Hz, $CDCl_3$): δ= 192.3, 138.1, 136.8, 132.9, 131.6, 131.1, 129.7, 52.9. HRMS (Q-TOF): *m/z* $[M + H]^+$ calcd for $C_{15}H_{11}Br_2O$: 364.9177, found: 364.9165.

Dimethyl 9-oxo-9*H*-dibenzo[3,4:6,7]cyclohepta[1,2-d]pyridazine-1,4-dicarboxylate (4a)

The reaction was performed according to the general procedure B with **3a** (1 g, 2.66 mmol) and PIFA (1.14 g, 2.66 mmol) at room teperature for 1 h. The product was purified by gradial chromatography (Al_2O_3, hexane then CH_2Cl_2/EtOAc (1:1)) and recrystallization from CH_3COOH to give **4a** as a colorless crystal (945 mg, 95% yield). mp = 224-225 ˚C. ^{1}H-NMR (400 M Hz, $CDCl_3$): δ= 7.76-7.72 (m, 2H), 7.66-7.55 (m, 6H), 3.80 (s 6H).^{13}C-NMR (100 M Hz, $CDCl_3$): δ= 195.3, 165.8, 154.9, 145.1, 133.2, 131.3, 131.1, 128.9, 128.5, 127.3, 53.3. HRMS (Q-TOF): m/z $[M + H]^+$ calcd. for $C_{21}H_{15}N_2O_5$: 375.0981, found: 375.0974.

1,4-Di(pyridin-2-yl)-9*H*-dibenzo[3,4:6,7]cyclohepta[1,2-*d*]pyridazin-9-one (4b)

The reaction was performed according to the general procedure B with **3b** (1 g, 2.41 mmol) and PIFA (1.04 g, 2.41 mmol) at room teperature °C for 1 h. The product was purified by gradial chromatography (Al_2O_3, hexane then MeOH/EtOAc (1:4)) and recrystallization from MeOH/diethyl ether (9:1) to give **4b** as a colorless crystal (896 mg, 90% yield). mp = 248 ˚C (decomposition). ^{1}H-NMR (400 M Hz, $CDCl_3$): δ= 8.49 (bd, *J* = 4.7 Hz, 2H), 7.73 (dt, *J* = 7.7 Hz, *J* = 1.7 Hz, 2H), 7.67-7.62 (m, 4H), 7.37 (dt, *J* = 7.6 Hz, *J* = 1.2 Hz, 2H), 7.27 (m, 2H), 7.05 (dt, *J* = 7.7 Hz, *J* = 1.3 Hz, 2H), 6.98 (bd, *J* = 7.8 Hz, 2H). ^{13}C-NMR (100 M Hz, $CDCl_3$): δ= 196.8, 158.8, 156.4, 149.3, 146.0, 136.5, 133.9, 131.7, 130.2, 129.53, 129.46, 126.2, 125.0, 123.2. HRMS (Q-TOF): m/z $[M + H]^+$ calcd. for $C_{27}H_{17}N_4O$: 413.1402, found: 413.1392.

1,4-Di(pyridin-4-yl)-9*H*-dibenzo[3,4:6,7]cyclohepta[1,2-*d*]pyridazin-9-one (4c)

The reaction was performed according to the general procedure B with **3c** (1.0 g, 2.41 mmol) and PIFA (1.56 g, 3.62 mmol) at room teperature °C for overnight. The product was purified by column chromatography (SiO_2, *n*-hexane/EtOAc (3:7)) and recrystallization from MeOH/diethyl ether (9:1) to give **4c** as a colorless crystal (866 mg, 87% yield). mp > 300 ˚C. ^{1}H-NMR (400 MHz, DMSO-d_6): δ= 8.59 (d, *J* = 4.7 Hz,

4H), 7.64 (d, J = 7.7 Hz, 2H), 7.55 (t, J = 7.5 Hz, 2H), 7.33 (d, J = 4.7 Hz, 4H), 7.24 (t, J = 7.6 Hz, 2H), 7.14 (d, J = 7.8 Hz, 2H). ^{13}C-NMR (100 MHz, DMSO-d_6): δ= 195.6, 157.6, 149.5, 145.6, 145.4, 133.2, 132.3, 130.6, 130.0, 128.6, 125.9, 124.3. HRMS (Q-TOF): m/z $[M + H]^+$ calcd. for $C_{27}H_{17}N_4O$: 413.1402, found: 413.1393.

1,4-Bis(3,5-dimethyl-1*H*-pyrazol-1-yl)-9*H*-dibenzo[3,4:6,7]cyclohepta[1,2-*d*]pyridazin-9-one (4d)

The reaction was performed according to the general procedure B with **3d** (500 mg, 1.11 mmol) and PIFA (575 mg, 1.34 mmol) at room temperature for 4 h. The product was purified by column chromatography (SiO_2, *n*-hexane/EtOAc (7:3)) and recrystallization from CH_2Cl_2/*n*-hexane (9:1) to give **4d** as a white crystal (453 mg, 91% yield). mp = 315-317 ˚C (decomposed). ^{1}H-NMR (400 MHz, $CDCl_3$): δ= 7.60 (d, J = 7.6 Hz, 2H), 7.45 (t, J = 7.5 Hz, 2H), 7.22 (t, J = 7.6 Hz, 2H), 6.87 (d, J = 7.9 Hz, 2H), 5.92 (s, 2H), 2.17 (s, 6H), 2.00 (s, 6H).^{13}C-NMR (100 MHz, $CDCl_3$): δ= 195.9, 153.9, 151.1, 146.0, 141.8, 133.9, 130.4, 130.4, 129.0, 127.9, 126.4, 107.7, 13.6, 11.4. HRMS (Q-TOF): m/z $[M + H]^+$ calcd. for $C_{27}H_{23}N_6O$: 447.1933, found: 447.1924.

9-Oxo-9*H*-dibenzo[3,4:6,7]cyclohepta[1,2-*d*]pyridazine-1,4-dicarboxamide (4e)

Nitrous gases, generated by adding conc. HCl (4.00 mL) to a solution of $NaNO_2$ (2.48 g) in water (6.00 mL), were bubbled at 0 °C through a solution of dihydropyridazine **3e** (100 mg, 0,30 mmol) in CH_2Cl_2 (20.0 mL). The reaction mixture was stirred at the same temperature for 1 h. Excess gases and the some solvent were removed under reduced pressure. After the precipitated product was filtered and wash with CH_2Cl_2, pyridazine **4e** (82,5 mg, 83%) was obtained as a white solid. mp > 300 ˚C. ^{1}H-NMR (400 MHz, DMSO-d_6): δ= 8.36 (bs, NH, 2H), 7.95 (bs, NH, 2H), 7.89-7.85 (m, 2H), 7.69-7.57 (m, 6H).^{13}C-NMR (100 MHz, DMSO-d_6): δ= 196.0, 167.5, 157.4, 144.68, 131.73, 130.9, 130.8, 129.5, 128.7, 125.9. HRMS (Q-TOF): m/z $[M + H]^+$ calcd. for $C_{19}H_{13}N_4O_3$: 345.0988, found: 345.0979.

Caution: When working with nitrous gases a well ventilated fume hood is essential.

9-Oxo-9*H*-dibenzo[3,4:6,7]cyclohepta[1,2-*d*]pyridazine-1,4-dicarbonitrile (4f)

The reaction was performed according to the general procedure B with **3f** ((500 mg, 1.61 mmol) and PIFA (693 mg, 1.61 mmol) at room temperature for overnight. The product was purified by column chromatography (SiO_2, *n*-hexane/EtOAc (4:1)) to give **4f** as a white solid (392 mg, 79% yield). mp > 300 ˚C. ^{1}H-NMR (400 MHz, DMSO-d_6): δ= 8.33 (d, J = 7.0 Hz, 2H), 7.91-7.82 (m, 4H), 7.77-7.72 (m, 2H). ^{13}C-NMR (100 MHz, DMSO-d_6): δ= 193.7, 145.1, 140.0, 138.0, 132.8, 131.5, 130.8, 127.0, 125.8, 115.7. HRMS (Q-TOF): m/z $[M + H]^+$ calcd. for $C_{19}H_9N_4O$: 309.0776, found: 309.0763.

Dimethyl 8-oxo-2,8-dihydrodibenzo[3,4:6,7]cyclohepta[1,2-*c*]pyrrole-1,3-dicarboxylate (10aa)

The reaction was performed according to the general procedure C with **4a** (500 mg, 1.34 mmol) and Zinc dust (436.6 mg, 6.68 mmol) at room temperature °C for overnight. The product was purified by column chromatography (SiO_2, EtOAc/*n*-Hexane (1:4) and recrystallization from CH_2Cl_2 to give **10aa** as a colorless crystal (369 mg, 82% yield). mp = 249-250 ˚C. ^{1}H-NMR (400 MHz, $CDCl_3$): δ= 10.11 (bs, NH, 1H), 7.80 (dd, *J* = 7.8 Hz, *J* = 1.1 Hz, 2H), 7.60 (dd, *J* = 7.5 Hz, *J* = 1.5 Hz, 2H), 7.49 (dt, *J* = 7.6 Hz, *J* = 1.6 Hz, 2H), 7.44 (dt, *J* = 7.5 Hz, *J* = 1.3 Hz, 2H), 3.86 (s, 6H). ^{13}C-NMR (100 MHz, $CDCl_3$): δ= 199.7, 160.2, 143.0, 131.4, 129.6, 128.5, 128.3, 127.7, 126.3, 121.3, 52.1. HRMS (Q-TOF): m/z $[M + H]^+$ calcd. for $C_{21}H_{16}NO_5$: 362.1028, found: 362.1028.

Dimethyl 8-hydroxy-2,8-dihydrodibenzo[3,4:6,7]cyclohepta[1,2-*c*]pyrrole-1,3-dicarboxylate 10ab

The reaction was performed according to the general procedure C with **4a** (500 mg, 1.34 mmol) and Zinc dust (873 mg, 13.36 mmol) at room temperature for overnight. The product was purified by column chromatography (SiO_2, EtOAc/*n*-Hexane (1:4) and recrystallization from CH_2Cl_2 to give **10aa** as a colorless crystal (359 mg, 74% yield). mp = 245-246 ˚C. ^{1}H-NMR (400 MHz, $CDCl_3$): δ= 10.08 (bs, 1H, NH), 7.72 (bd, *J* = 7.8 Hz, 2H), 7.57 (dd, *J* = 7.6 Hz, *J* = 0.8 Hz, 2H), 7.37 (dt, *J* = 7.7 Hz, *J* = 1.0 Hz, 2H), 7.21 (dt, *J* = 7.5 Hz, *J* = 1.1 Hz, 2H), 5.45 (s, 1H), 3.84 (s, 6H), 2.25 (bs, 1H). ^{13}C-NMR (100 MHz, $CDCl_3$): δ= 160.7, 143.7, 130.9, 129.1, 128.4, 126.7, 125.5, 120.7, 120.0, 70.3, 52.0. HRMS (Q-TOF): m/z $[M + H]^+$ calcd. for $C_{21}H_{18}NO_5$: 364.1185, found: 364.1171.

2,8-Dihydrodibenzo[3,4:6,7]cyclohepta[1,2-*c*]pyrrol-8-ol (10ac)

A 10 mL round bottom microwave vial equipped with a stir bar was charged with pyrrole **10ab** (200 mg 0.55 mmol), KOH (123.5 mg, 2.20 mmol) and 5 mL THF/CH_3OH/H_2O (2:2:1) solvent mixture. The vial was sealed and the reaction was irradiated (200W) in the microwave reactor at 150 ˚C for 2 h. The reaction mixture was extracted with EtOAc (2 × 25). The combined organic layer was dried over Na_2SO_4 and the solvent was evaporated under reduced pressure to give **10ac** as a brown solid (97mg, 70% yield). ^{1}H-NMR (400 MHz, DMSO-d_6): δ= 11.40 (s, NH, 1H), 7.70 (d, *J* = 7.4 Hz, 2H), 7.39 (d, *J* = 7.2 Hz, 2H), 7.30-7.13 (m, 6H), 5.96 (d, *J* = 3.9 Hz, 1H), 5.28 (bs, 1H). ^{13}C-NMR (100 MHz, DMSO-d_6): δ= 142.4, 132.1, 127.27, 126.8, 126.2, 123.2, 122.6, 115.7, 69.8. HRMS (Q-TOF): m/z $[M + Na]^+$ calcd. for $C_{17}H_{14}NO$: 270.0895, found: 270.0900.

1,3-Di(pyridin-2-yl)-2,8-dihydrodibenzo[3,4:6,7]cyclohepta[1,2-*c*]pyrrol-8-yl acetate (10ba)

The reaction was performed according to the general procedure C with **4b** (500 mg, 1.21 mmol) and Zinc dust (1.59 g, 24.25 mmol) at 118 °C for 2 h. The product was crystallized from MeOH/diethyl ether (9:1) to give **10ba** as a yellow crystal (409 mg, 76% yield). mp = 254-255 ˚C. ^{1}H-NMR (400 MHz, $CDCl_3$): δ= 10.68 (bs, NH, 1H), 8.56 (bd, *J* = 4.7 Hz, 2H), 7.53 (d, *J* = 7.8 Hz, 2H), 7.47 (d, *J* = 8.1 Hz, 2H), 7.38 (dt, *J* = 7.6 Hz, *J* = 1.7 Hz, 2H), 7.43 (d, *J* = 7.6 Hz, 2H), 7.23 (dt, *J* = 7.6 Hz, *J* = 1.0 Hz, 2H), 7.08-6.99 (m, 4H), 6.77 (s, 1H), 2.34 (s, 3H). ^{13}C-NMR (100 MHz, $CDCl_3$): δ=169.6, 150.4, 149.5, 139.8, 135.9, 130.1, 129.6, 128.4, 127.4, 126.5, 122.6, 121.4, 121.2, 121.1, 72.4, 21.3. HRMS (Q-TOF): m/z $[M + H]^+$ calcd. for $C_{29}H_{22}N_3O_2$: 444.1712, found: 444.1702.

1,3-Di(pyridin-2-yl)-2,8-dihydrodibenzo[3,4:6,7]cyclohepta[1,2-*c*]pyrrol-8-ol (10bb)

To a solution of **10ba** (500 mg, 1.13 mmol) in 10 mL of H_2O/EtOH (1:3) was added KOH (94.88 mg, 1.69 mmol) and stirred at room temperature for 4h. The reaction mixture was extracted with EtOAc (2 × 25). The combined organic layer was dried over Na_2SO_4 and evaporated under vacuo. The product was purified by column chromatography (SiO_2, EtOAc/*n*-Hexane (4:6) and recrystallization from CH_2Cl_2/*n*-hexane (9:1) to give **10bb** as a yellow crystal (363 mg, 80% yield). mp = 273-274 ˚C. ^{1}H-NMR (400 MHz, $CDCl_3$): δ= 10.76 (bs, NH, 1H), 8.62 (d, *J* = 4.6 Hz, 2H), 7.81 (d, *J* = 7.8 Hz, 2H), 7.56 (d, *J* = 8.1 Hz, 2H), 7.50-7.30 (m, 4H), 7.34 (t, *J* = 7.6 Hz, 2H), 7.15-7.06 (m, 4H), 5.90 (s, 1H), 2.51 (s, 1H). ^{13}C-NMR (100 MHz, $CDCl_3$): δ=150.6, 149.6, 143.3, 136.0, 129.9, 129.4, 128.1, 127.5, 126.2, 123.2, 121.4(2C), 121.0, 70.7. HRMS (Q-TOF): m/z $[M + H]^+$ calcd. for $C_{27}H_{20}N_3O$: 402.1606, found: 402.1605.

1,3-Di(pyridin-2-yl)dibenzo[3,4:6,7]cyclohepta[1,2-*c*]pyrrol-8(2*H*)-one (10bc)

To a solution of **10bb** (500 mg, 1.25 mmol) in 10 mL of CH_2Cl_2 was added MnO_2 (1.08 g, 12.45 mmol) and stirred at room temperature for 3h. The solvent was evaporated under reduced pressure and the reaction mixture was filtered through Celite. The product was crystallized from MeOH/diethyl ether (9:1) to give **10ba** as a yellow crystal (388 mg, 78% yield). mp = 244-245 ˚C. ^{1}H-NMR (400 MHz, $CDCl_3$): δ= 10.50 (bs, NH, 1H), 8.64 (d, *J* = 4.6 Hz, 2H), 7.69 (d, *J* = 7.3 Hz, 2H), 7.63 (d, *J* = 7.5 Hz, 2H), 7.47 (t, *J* = 7.8 Hz, 2H), 7.41-7.30 (m, 6H), 7.15-7.09 (m, 2H). ^{13}C-NMR (100 MHz, $CDCl_3$): δ= 200.8, 150.6, 149.7, 142.5, 135.9, 131.4, 130.4, 130.1, 130.0, 127.4, 127.3, 121.7, 121.4, 121.2. HRMS (Q-TOF): m/z $[M + H]^+$ calcd. for $C_{27}H_{18}N_3O$: 400.1450, found: 400.1440.

Dimethyl 9-(4-hydroxyphenyl)-9*H*-dibenzo[3,4:6,7]cyclohepta[1,2-*d*]pyridazine-1,4-dicarboxylate (13a)

The reaction was performed according to the general procedure A with **11** (500 mg, 1.77 mmol) and DET **2a** (351 mg, 1.77 mmol) in 5 mL toluene at 80 °C for overnight.

The product was purified by column chromatography (SiO_2, *n*-hexane/EtOAc (1,5:8,5) to give **13a** as a white solid (697 mg, 95% yield). mp = 270-271 ˚C. ^{1}H-NMR (400 MHz, $CDCl_3$): δ= 7.55-7.47 (m, 4H), 7.38 (d, *J* = 7.5 Hz, 2H), 7.32 (t, *J* = 7.4 Hz, 2H), 6.68 (d, *J* = 8.5 Hz, 2H), 6.41 (d, *J* = 8.5 Hz, 2H), 5.37 (s, 1H), 4.92 (s, 1H), 3.85 (s, 6H). ^{13}C-NMR (100 MHz, $CDCl_3$): δ= 166.0, 154.0, 152.7, 147.5, 135.3, 130.9, 130.8, 130.1, 129.5, 129.3, 128.8, 127.0, 114.5, 56.3, 53.0. HRMS (Q-TOF): m/z $[M + H]^+$ calcd. for $C_{27}H_{21}N_2O_5$: 453.1450, found: 453.1448.

4-(1,4-Di(pyridin-2-yl)-9*H*-dibenzo[3,4:6,7]cyclohepta[1,2-*d*]pyridazin-9-yl)phenol (13b)

The reaction was performed according to the general procedure A with **11** (500 mg, 1.77 mmol) and DPT **2b** (841 mg, 1.77 mmol) in 5 mL toluene at 80 °C for 3 day. The mixture was cooled to room temperature and the precipitated product was filtrated, washed with Et_2O to give **13b** as a white solid (808 mg, 93% yield). mp > 300 ˚C. ^{1}H-NMR (400 MHz, $CDCl_3$): δ= 8.61 (d, *J* = 4.6 Hz, 2H), 7.82 (t, *J* = 7.8 Hz, 2H), 7.55 (d, *J* = 7.5 Hz, 2H), 7.46-7.39 (m, 2H), 7.37 (d, *J* = 7.8 Hz, 2H), 7.32 (t, *J* = 7.5 Hz, 2H), 7.22 (d, *J* = 8.4 Hz, 2H), 6.88 (t, *J* = 7.6 Hz, 2H), 6.83 (d, *J* = 7.6 Hz, 2H), 6.52 (d, *J* = 8.4 Hz, 2H), 5.53 (s, 1H). ^{13}C-NMR (100 MHz, $CDCl_3$): δ= 158.6, 157.5, 156.8, 150.2, 149.5, 138.1, 138.0, 133.7, 132.1, 131.3, 130.8, 130.6, 130.4, 126.8, 126.2, 124.9, 115.5, 57.9. HRMS (Q-TOF): m/z $[M + H]^+$ calcd. for $C_{33}H_{23}N_4O$: 491.1872, found: 491.1872.

Dimethyl 9-(4-oxocyclohexa-2,5-dien-1-ylidene)-9*H*-dibenzo[3,4:6,7]cyclohepta[1,2-*d*]pyridazine-1,4-dicarboxylate (14a)

The reaction was performed according to the general procedure B with **13a** (500 mg, 1.11 mmol) and PIFA (570 mg,1.33 mmol) at room temperature for overnight. The product was purified by gradial chromatography (Al_2O_3, *n*-hexane then CH_2Cl_2/EtOAc (9:1)) and recrystallization from CH_2Cl_2/*n*-hexane (9:1) to give **14a** as a yellow crystal (453 mg, 90% yield). mp = 253-254 ˚C. ^{1}H-NMR (400 MHz, $CDCl_3$): δ= 7.59 (t, *J* = 7.5 Hz, 2H), 7.54 (d, *J* = 7.7 Hz, 2H), 7.47 (t, *J* = 7.7 Hz, 2H), 7.42-7.35 (m, 4H), 6.43 (d, *J* = 10.0 Hz, 2H), 3.89 (s, 6H).^{13}C-NMR (100 MHz, $CDCl_3$): δ= 186.8, 165.9, 154.2, 151.6, 143.2, 136.4, 134.7, 130.8, 130.5, 129.7, 129.2, 129.2, 128.8, 128.0, 77.5, 77.2, 76.8, 53.5. HRMS (Q-TOF): m/z $[M + H]^+$ calcd. for $C_{27}H_{19}N_2O_5$: 451.1294, found: 451.1283.

4-(1,4-Di(pyridin-2-yl)-9*H*-dibenzo[3,4:6,7]cyclohepta[1,2-*d*]pyridazin-9-ylidene)cyclohexa-2,5-dien-1-one (14b)

The reaction was performed according to the general procedure B with **13b** (500 mg, 1.02 mmol) and PIFA (526 mg,1.22 mmol) at room temperature for overnight. The product was purified by gradial chromatography (Al_2O_3, *n*-hexane then EtOAc/MeOH (9:1)) and recrystallization from then MeOH/diethyl ether (9:1) to give **14b** as a yellow

crystal (433 mg, 87% yield). mp = 272-273 ˚C. ^{1}H-NMR (400 MHz, $CDCl_3$): δ= 8.48 (d, *J* = 4.7 Hz, 2H), 7.79-7.74 (m, 4H), 7.64 (d, *J* = 10.1 Hz, 2H), 7.35-7.23 (m, 6H), 7.01-6.95 (m, 2H), 6.92 (d, *J* = 7.6 Hz, 2H), 6.51 (d, *J* = 10.1 Hz, 2H). ^{13}C-NMR (100 MHz, $CDCl_3$): δ= 187.0, 158.3, 156.3, 154.5, 149.3, 143.7(2C), 137.1, 136.5, 135.0, 131.4, 130.9, 130.10, 128.8, 127.3, 126.9, 124.9, 123.2. HRMS (Q-TOF): m/z $[M + H]^+$ calcd. for $C_{33}H_{21}N_4O$: 489.1715, found: 489.1700.

Dimethyl 8-(4-hydroxyphenyl)-2,8-dihydrodibenzo[3,4:6,7] cyclohepta[1,2-c]pyrrole-1,3-dicarboxylate (15a)

The reaction was performed according to the general procedure C with **13a** (500 mg, 1.1 mmol) and Zinc dust (722 mg, 11.1 mmol) at room temperature for overnight. The product was crystallized from MeOH/diethyl ether (9:1) to give **15a** as a white crystal (301 mg, 62% yield). mp = 302-303 ˚C. ^{1}H-NMR (400 MHz, Acetone-d_6): δ= 10.86 (s, NH, 1H), 7.82 (s, OH, 1H), 7.63 (d, *J* = 7.5 Hz, 2H), 7.54 (d, *J* = 7.5 Hz, 2H), 7.35 (t, *J* = 7.5 Hz, 2H), 7.25 (t, *J* = 7.5 Hz, 2H), 6.55 (d, *J* = 8.5 Hz, 2H), 6.36 (d, *J* = 8.5 Hz, 2H), 5.33 (s, 1H), 3.74 (s, 6H). ^{13}C-NMR (100 MHz, Acetone-d_6): δ= 161.1, 155.8, 144.9, 133.3, 133.0, 130.8, 130.5, 129.9, 128.8, 128.5, 126.6, 120.9, 114.7, 57.7, 51.7. HRMS (Q-TOF): m/z $[M + H]^+$ calcd. for $C_{27}H_{22}NO_5$: 440.1498, found: 440.1490.

4-(1,3-Di(pyridin-2-yl)-2,8-dihydrodibenzo[3,4:6,7]cyclohepta[1,2-*c*]pyrrol-8-yl)phenol (15b)

The reaction was performed according to the general procedure C with **13b** (500 mg, 1.02 mmol) and Zinc dust (666 mg, 10.2 mmol) at room temperature for overnight. The product was crystallized from MeOH/diethyl ether (9:1) to give **15b** as a brown crystal (278 mg, 57% yield). mp = 303-304 ˚C. ^{1}H-NMR (400 MHz, $CDCl_3$): δ= 10.15 (s, NH, 1H), 8.51 (d, *J* = 4.6 Hz, 2H), 7.52 (d, *J* = 7.5 Hz, 2H), 7.49 (d, *J* = 7.7 Hz, 2H), 7.43 (dt, *J* = 7.7 Hz, *J* = 1.2 Hz, 2H), 7.32 (d, *J* = 7.5 Hz, 2H), 7.31 (t, *J* = 7.1 Hz, 2H), 7.16 (t, *J* = 7.5 Hz, 2H), 7.07-6.97 (m, 2H), 6.82 (d, *J* = 8.5 Hz, 2H), 6.34 (d, *J* = 8.5 Hz, 2H), 5.32 (s, 1H). ^{13}C-NMR (100 MHz, $CDCl_3$): δ= 153.1, 150.8, 149.3, 143.2, 135.7, 134.1, 131.9, 131.5, 130.0, 127.7, 127.6, 127.4, 126.6, 123.6, 121.0, 120.6, 114.1, 57.6. HRMS (Q-TOF): m/z $[M + H]^+$ calcd. for $C_{33}H_{24}N_3O$: 478.1919, found: 478.1909.

Dimethyl 8-(4-oxocyclohexa-2,5-dien-1-ylidene)-2,8-dihydrodibenzo[3,4:6,7]cyclohepta[1,2-*c*]pyrrole-1,3-dicarboxylate (16a)

The reaction was performed according to the general procedure B with **15a** (500 mg, 1.02 mmol) and PIFA (526 mg,1.22 mmol) at room temperature for overnight. The product was purified by gradial chromatography (Al_2O_3, *n*-hexane then CH_2Cl_2) and recrystallization from then CH_2Cl_2/*n*-hexane (9:1) to give **16a** as a yellow crystal (444 mg, 89% yield). mp = 303-304 ˚C. ^{1}H-NMR (400 MHz, $CDCl_3$): δ= 9.98 (s, NH, 1H), 7.85-7.75 (m, 2H), 7.47 (d, *J* = 10.0 Hz, 2H), 7.44-7.36 (m, 4H), 7.29-7.23 (m, 2H), 6.37 (d, *J* = 10.0 Hz, 2H), 3.88 (s, 6H). ^{13}C-NMR (100 MHz, $CDCl_3$): δ= 187.3, 160.4,

158.2, 139.8, 137.8, 131.8, 129.4, 129.0, 128.9, 128.8, 128.2, 127.7, 127.4, 120.7, 52.2. HRMS (Q-TOF): m/z $[M + H]^+$ calcd. for $C_{27}H_{20}NO_5$: 438.1341, found: 438.1331.

4-(1,3-Di(pyridin-2-yl)dibenzo[3,4:6,7]cyclohepta[1,2-*c*]pyrrol-8(2*H*)-ylidene)cyclohexa-2,5-dien-1-one (16b)

DDQ (238 mg, 1.05 mmol) and **15b** (500 mg, 1.05 mmol) were dissolved in 20 mL CH_2Cl_2 and stirred at room temperature for 30 min. The reaction mixture was washed with 20 mL of 5% $NaHCO_3$ solution and H_2O (2x20 mL). The organic phase was dried over Na_2SO_4 and the solvent was evaporated under reduced pressure to give **16b** as a yellow solid (483 mg, 97% yield). mp > 300 ˚C. ^{1}H-NMR (400 MHz, $CDCl_3$): δ= 10.60 (s, NH, 1H), 8.62 (d, *J* = 4.7 Hz, 2H), 7.66–7.58 (m, 4H), 7.50 (dt, *J* = 7.8 Hz, *J* = 1.7 Hz, 2H), 7.43 (d, *J* = 8.0 Hz, 2H), 7.40-7.31 (m, 4H), 7.31-7.24 (m, 2H), 7.11 (dd, *J* = 6.4 Hz, *J* = 5.5 Hz, 2H), 6.43 (d, *J* = 10.1 Hz, 2H).^{13}C-NMR (100 MHz, $CDCl_3$): δ= 187.3, 159.8, 150.5, 149.8, 139.3, 137.8, 136.0, 131.8, 130.7, 129.4, 129.2, 128.5, 128.4, 128.3, 127.2, 122.4, 121.7, 121.1. HRMS (Q-TOF): m/z $[M + H]^+$ calcd. for $C_{33}H_{22}N_3O$: 476.1763, found: 476.1750.

Table 1. Some photophysical properties of cycloadducts **3c-3f** and **3k.**

Compound	λ_{ems}/nm (λ_{exc}/nm)	λ_{abs}/nm	Stokes shift (nm)	Quantum yields (Φ_F)	ε ($M^{-1}.cm^{-1}$)
**3c**	534 (400)	427	107	0.78	5867
**3d**	539 (375)	408	131	0.60	4987
**3e**	515 (360)	393	122	0.53	3749
**3f**	487 (350)	378	109	0.28	6044
**3k**	503 (350)	378	125	0.16	5261

^{1}H NMR, ^{13}C NMR, and HRMS Spectra:

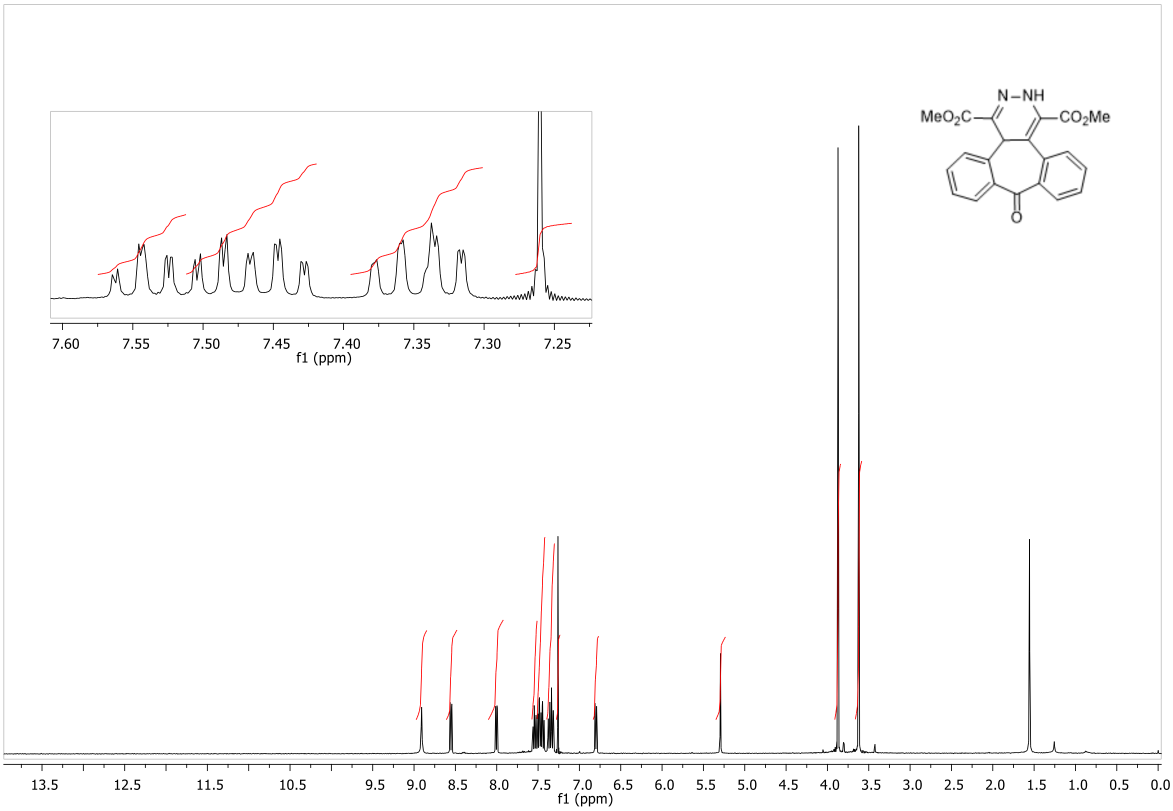

Fig. S1. ^{1}H-NMR spectrum of **3a** (400 MHz, $CDCl_3$).

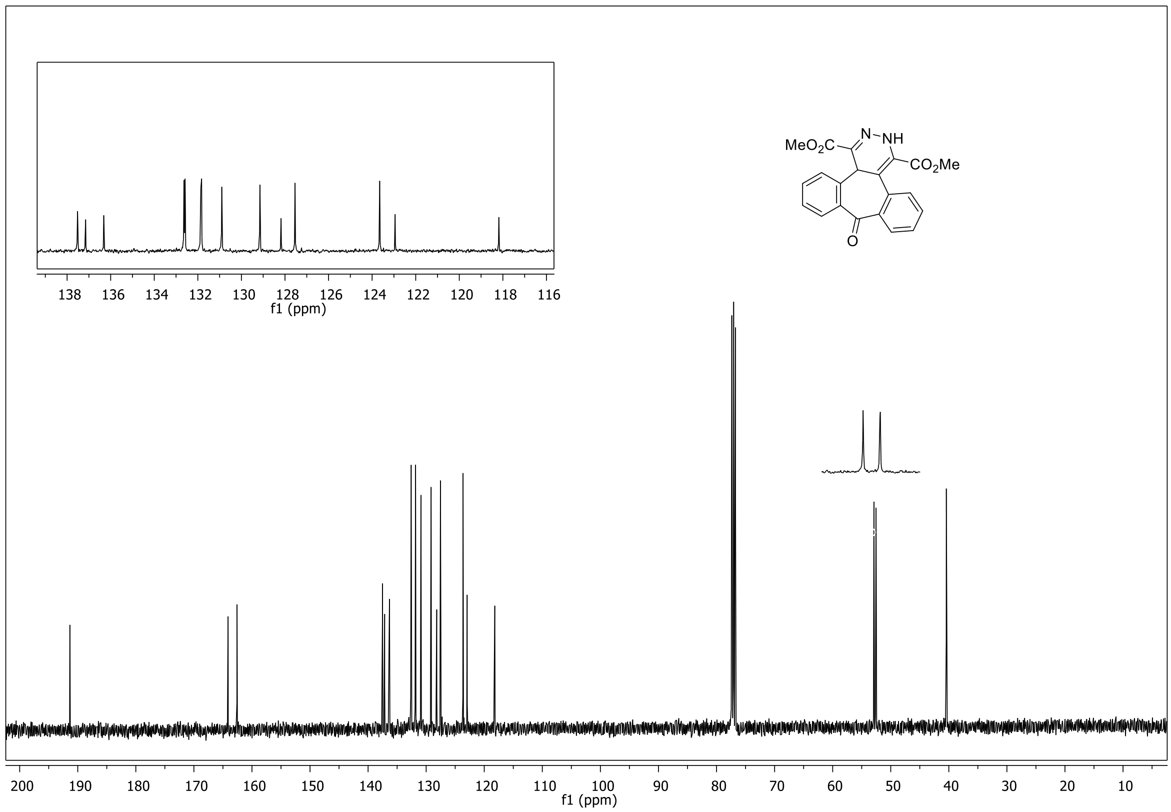

Fig. S2. ^{13}C-NMR spectrum of **3a** (100 MHz, $CDCl_3$).

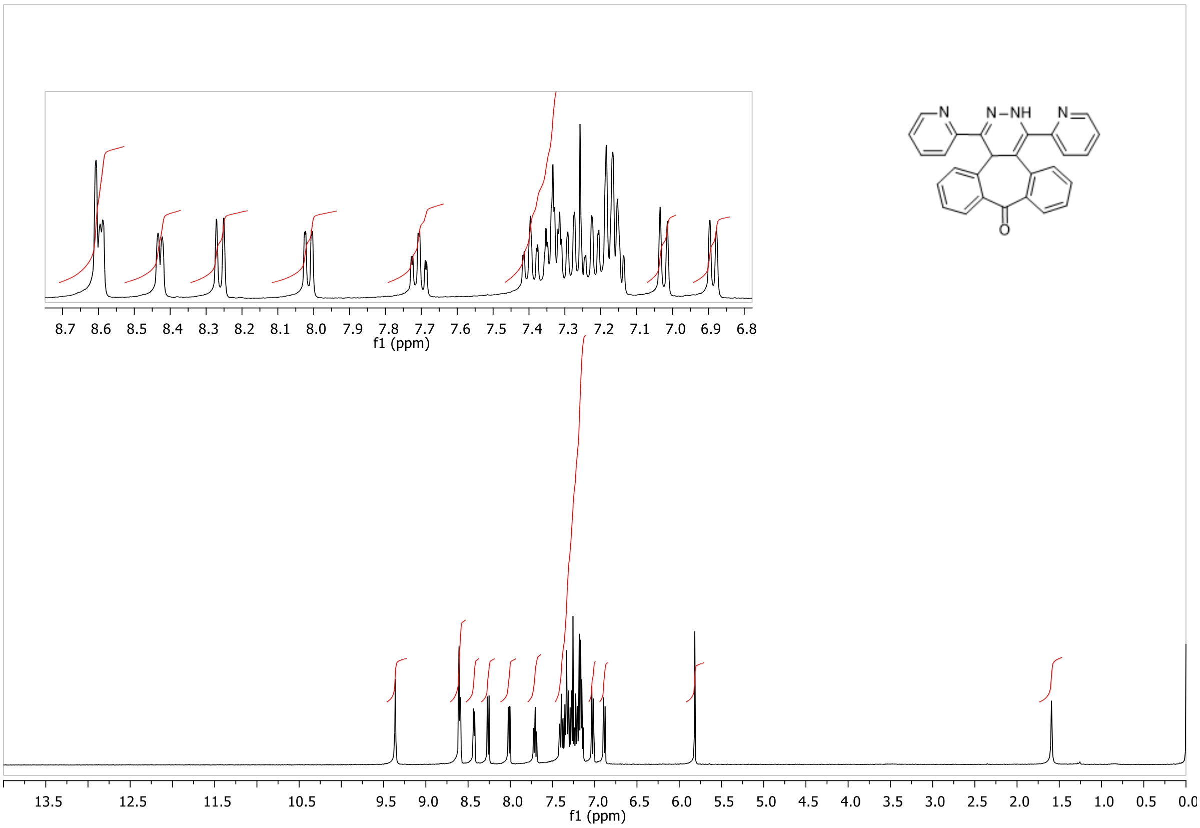

Fig. S3. ^{1}H-NMR spectrum of **3b** (400 MHz, $CDCl_3$).

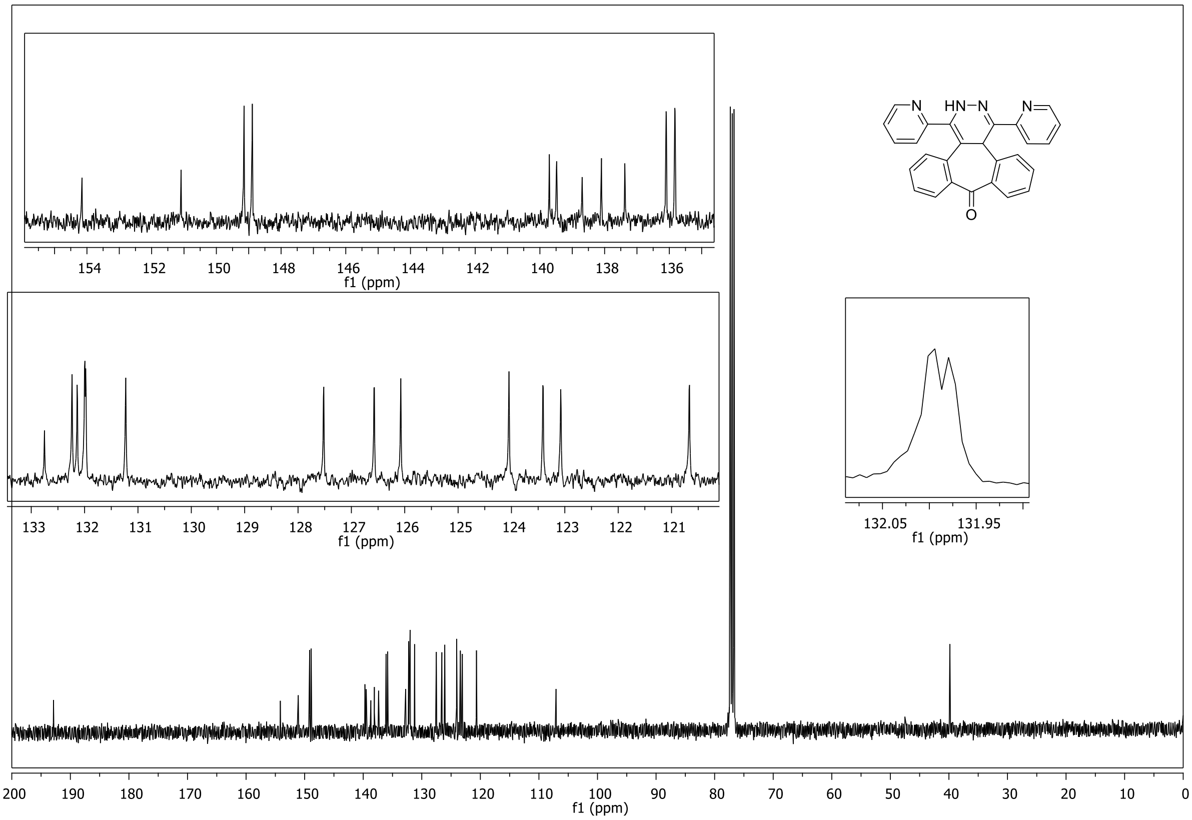

Fig. S4. ^{13}C-NMR spectrum of **3b** (100 MHz, $CDCl_3$).

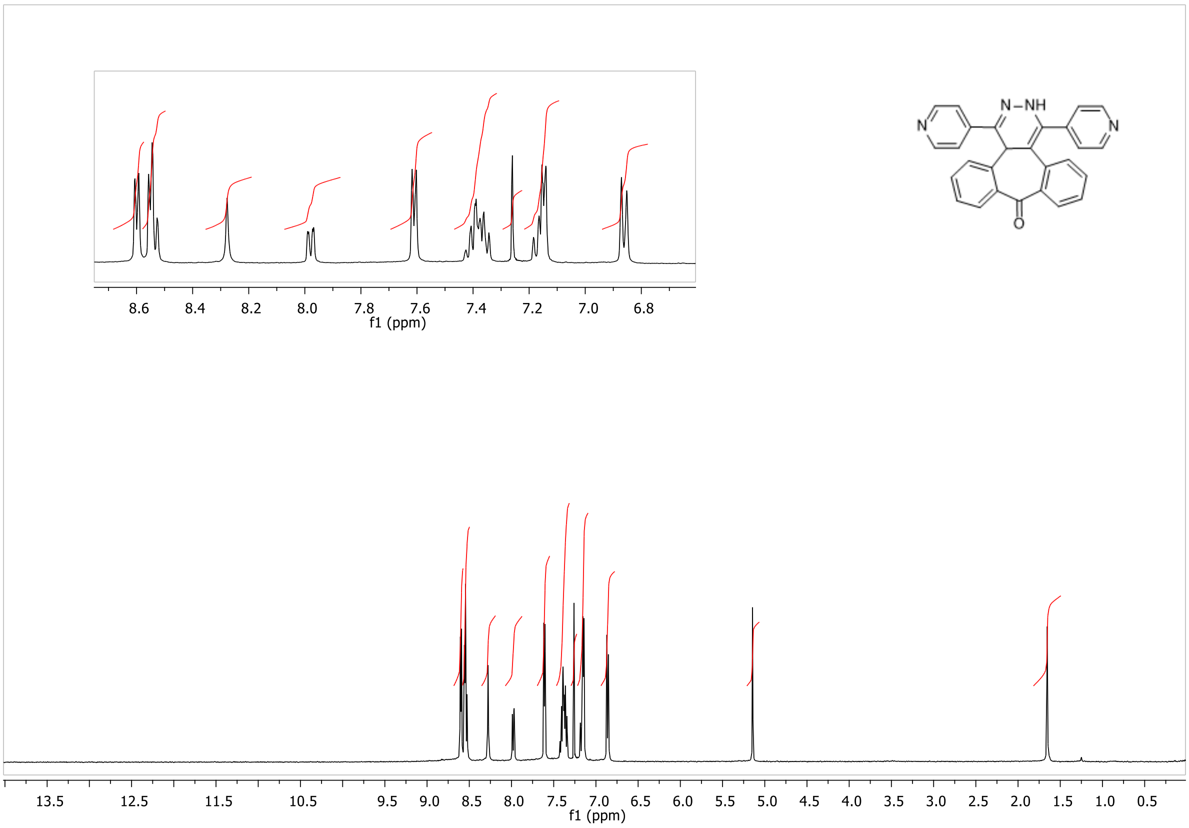

Fig. S5. ^{1}H-NMR spectrum of **3c** (400 MHz, $CDCl_3$).

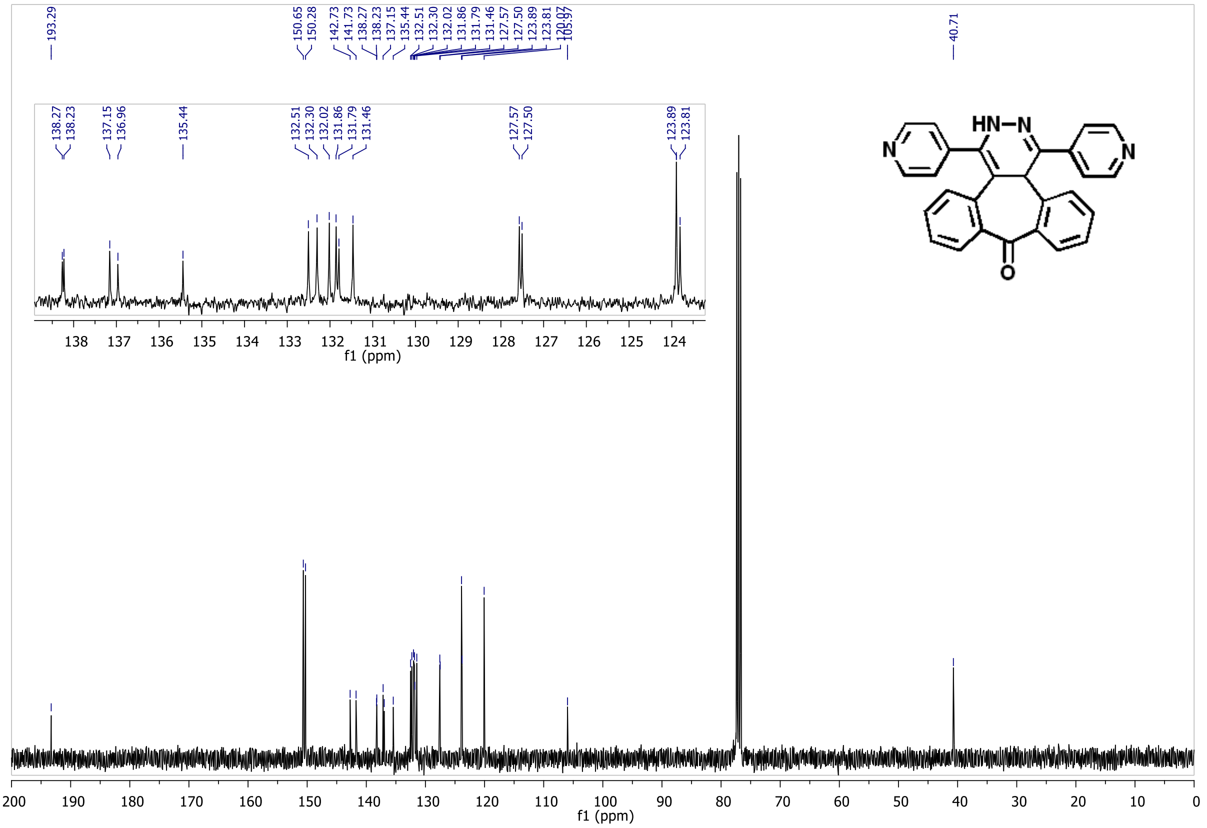

Fig. S6. ^{13}C-NMR spectrum of **3c** (100 MHz, $CDCl_3$).

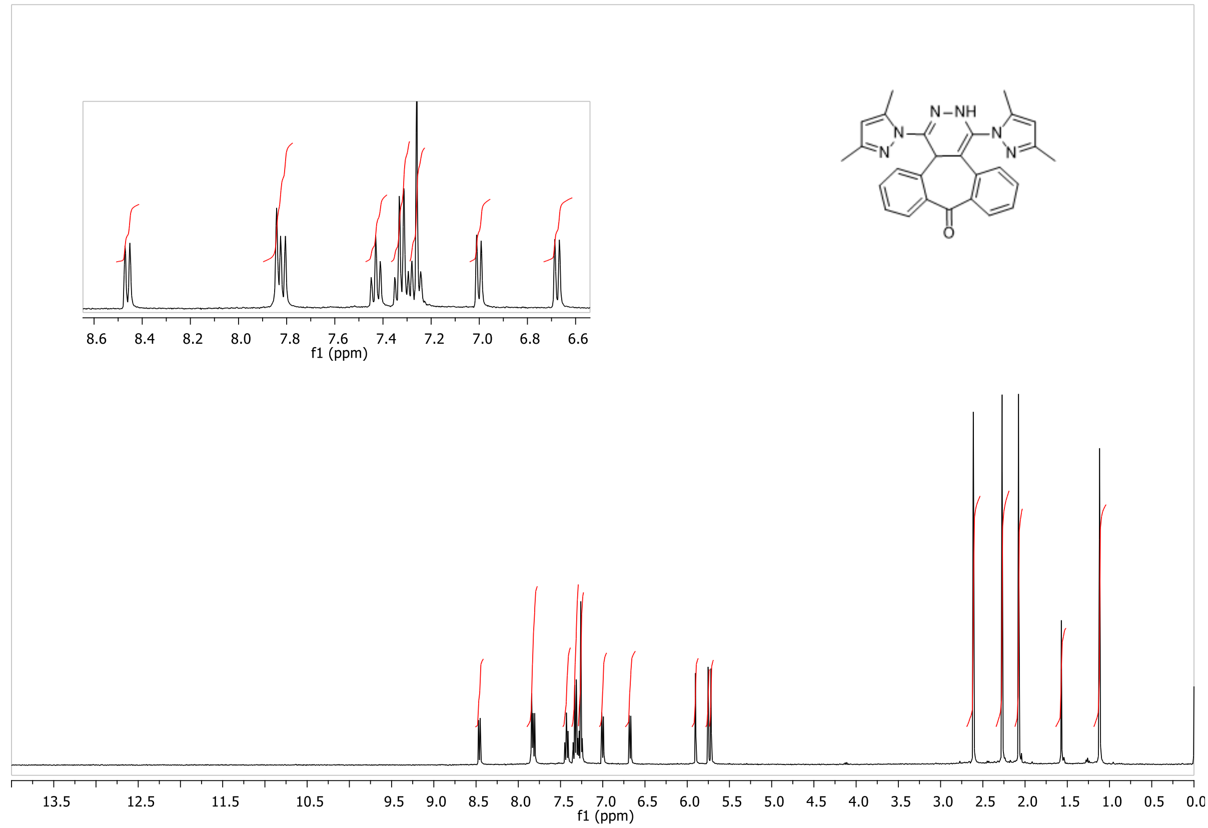

Fig. S7. ^{1}H-NMR spectrum of **3d** (400 MHz, $CDCl_3$).

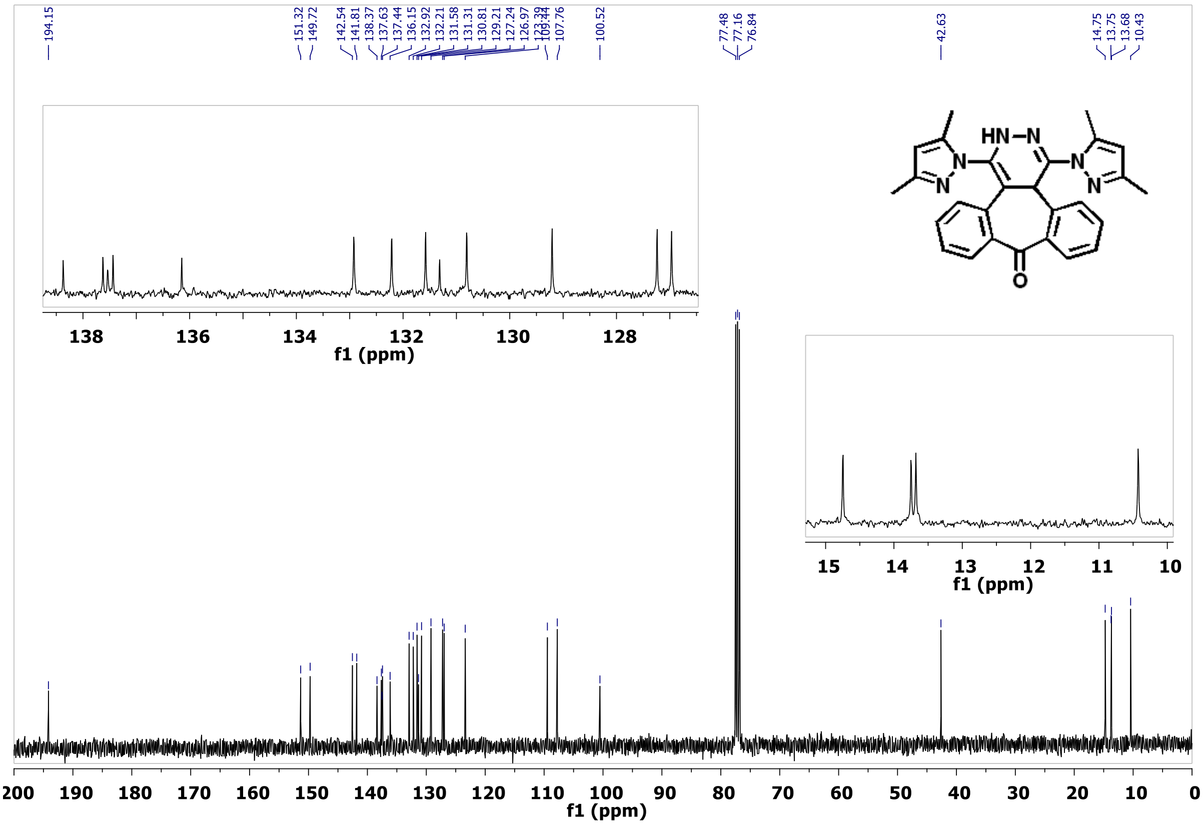

Fig. S8. ^{13}C-NMR spectrum of **3d** (100 MHz, $CDCl_3$).

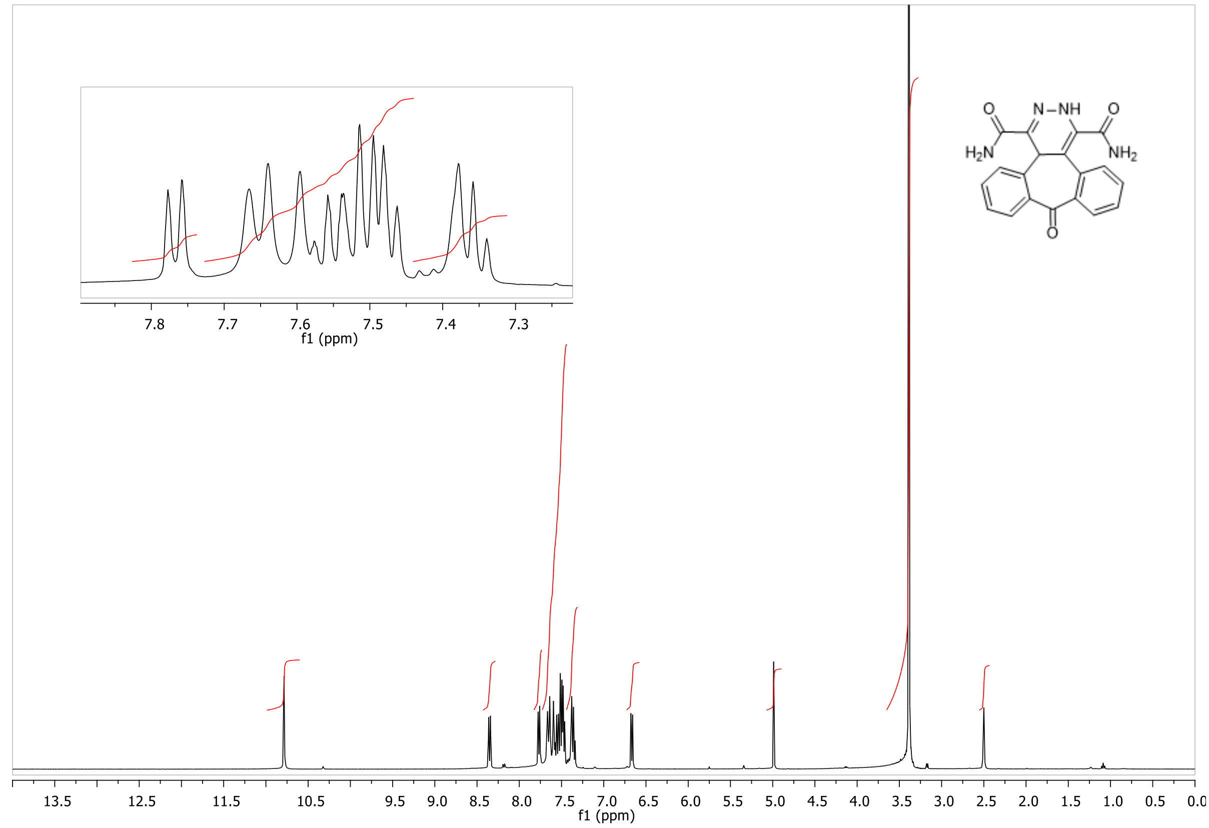

Fig. S9. ^{1}H-NMR spectrum of **3e** (400 MHz, DMSO-d_6).

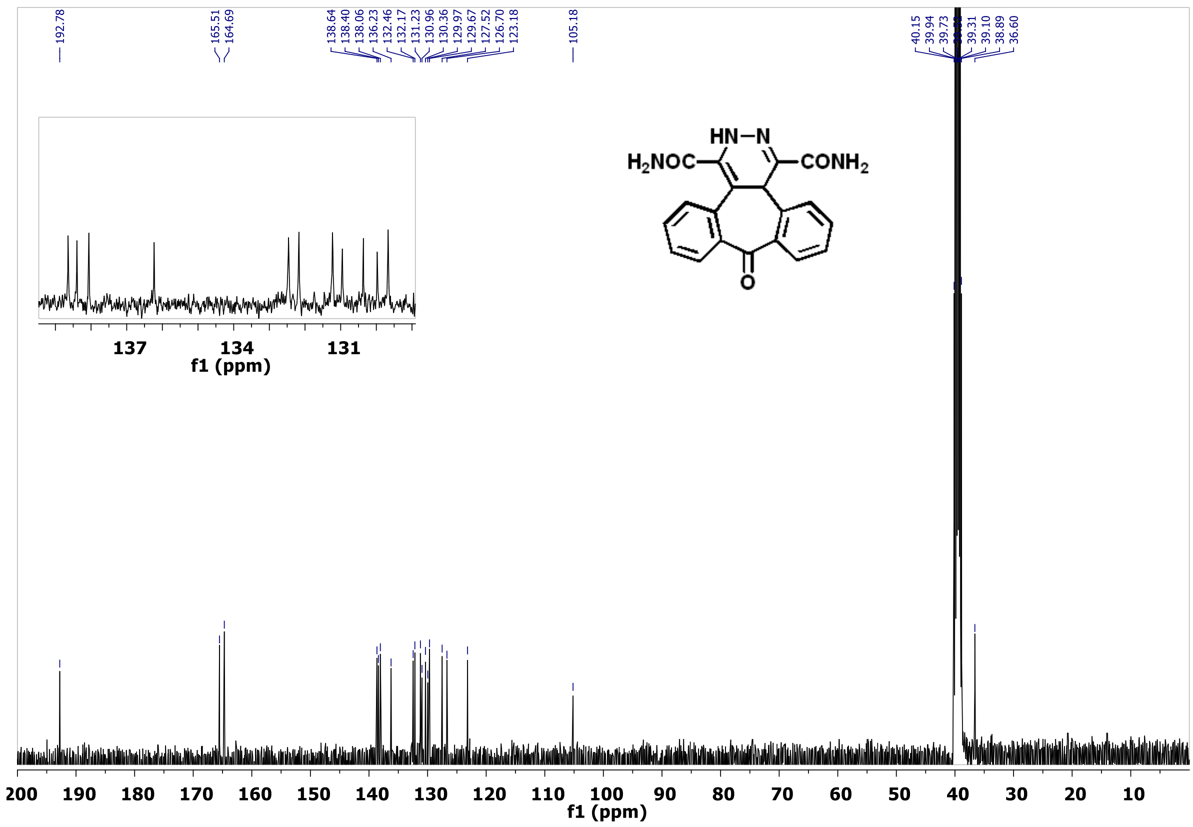

Fig. S10. ^{13}C-NMR spectrum of **3e** (100 MHz, DMSO-d_6).

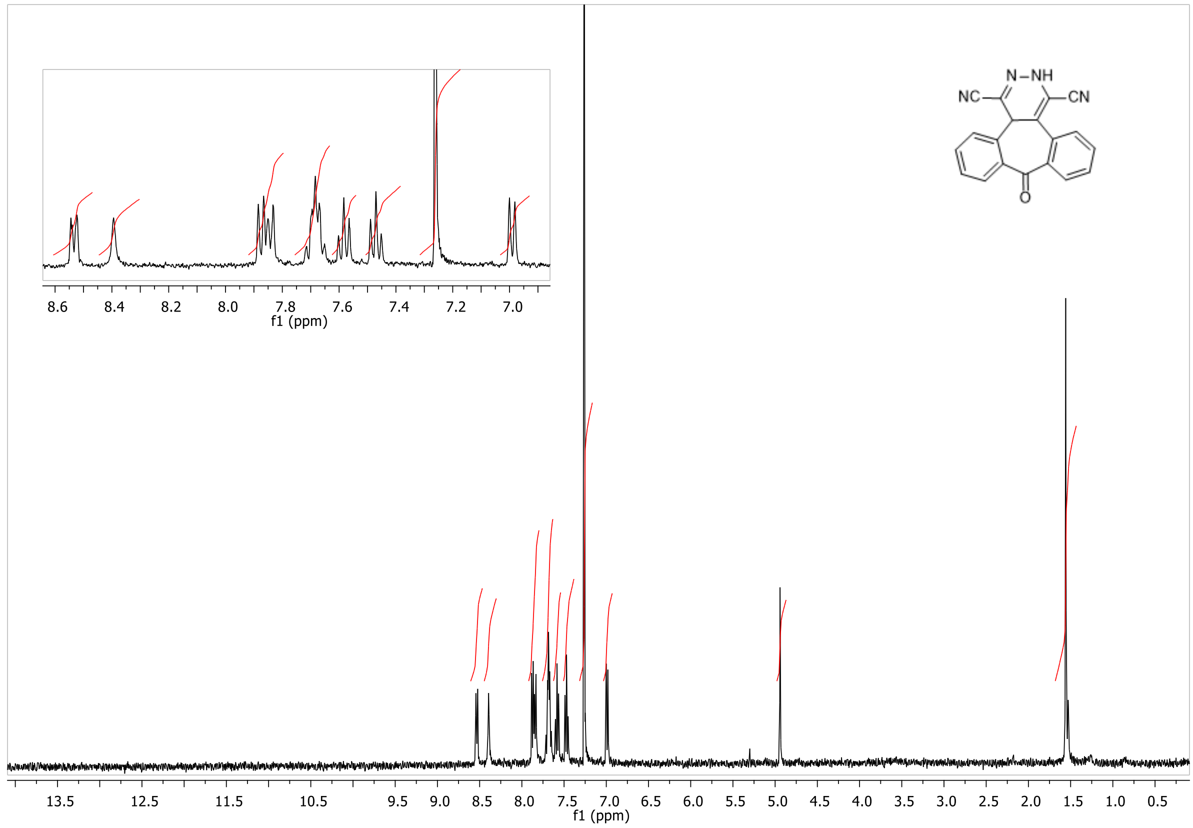

Fig. S11. ^{1}H-NMR spectrum of **3f** (400 MHz, $CDCl_3$).

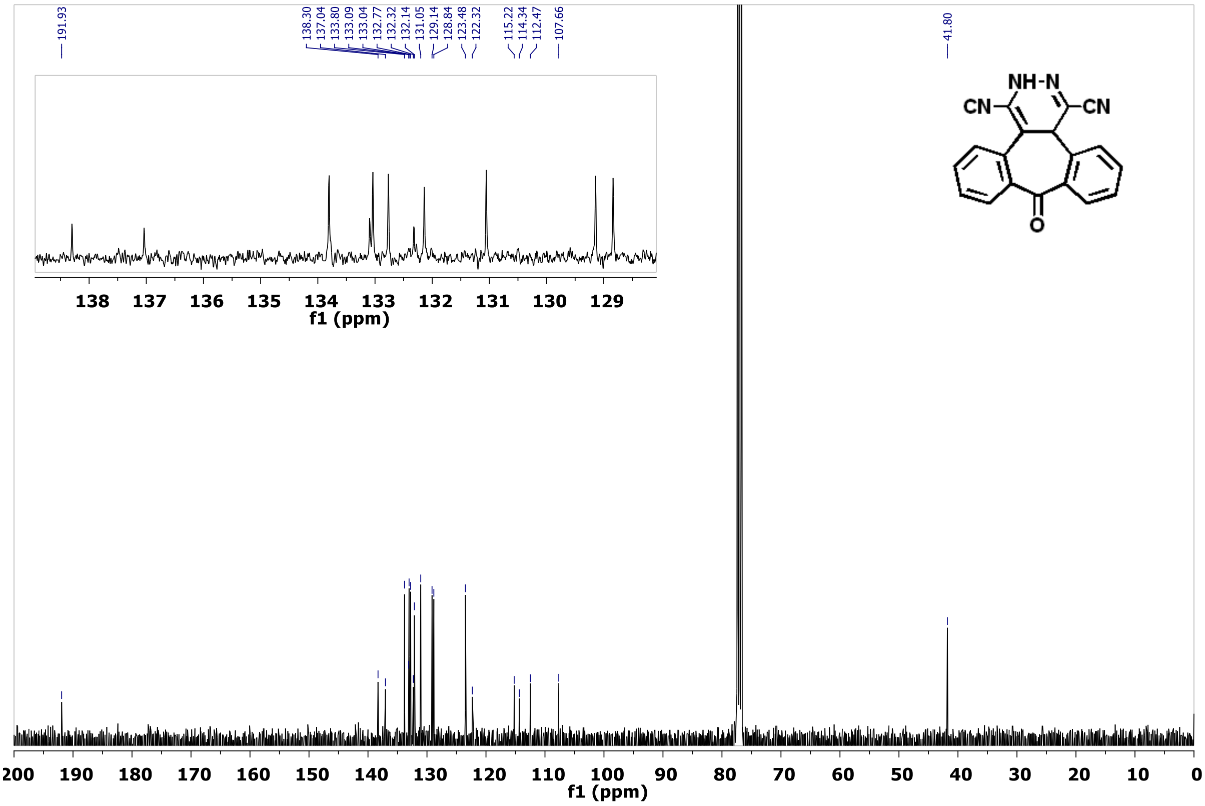

Fig. S12. ^{13}C-NMR spectrum of **3f** (100 MHz, $CDCl_3$).

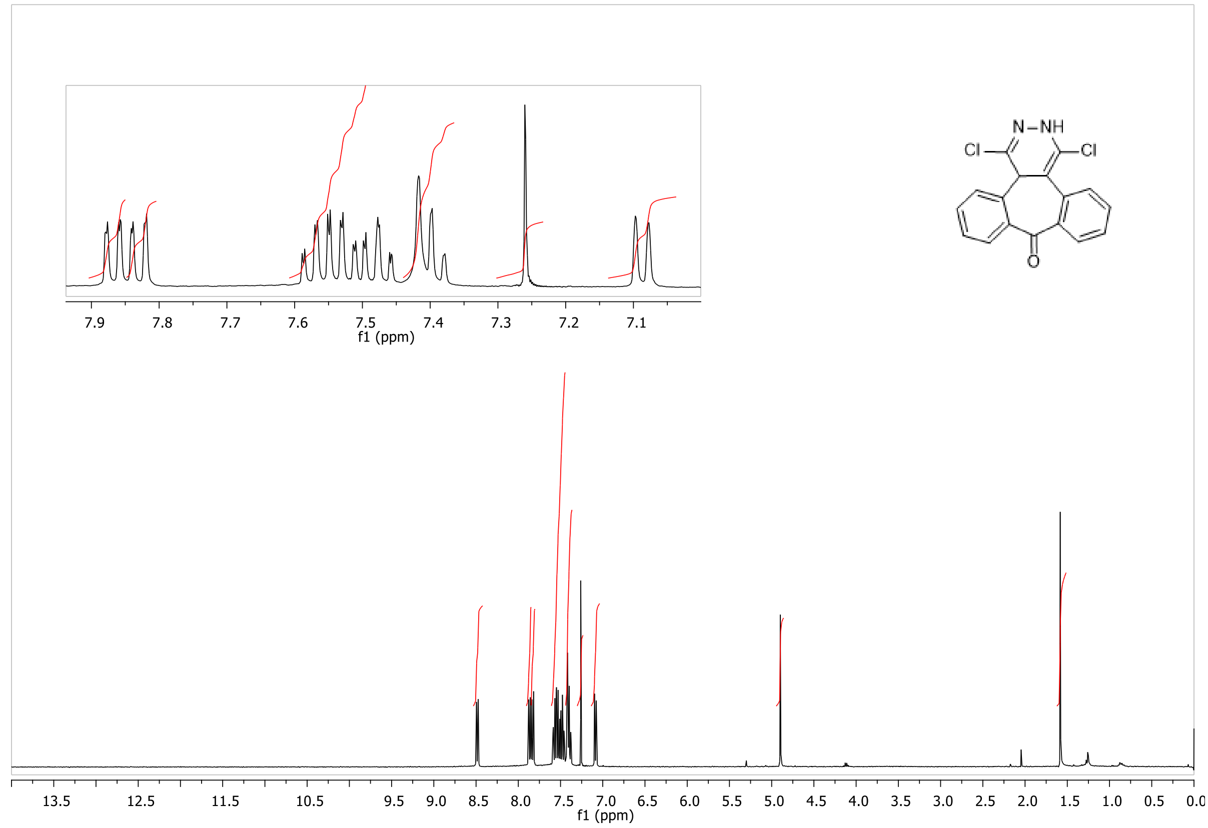

Fig. S13. ^{1}H-NMR spectrum of **3k** (400 MHz, $CDCl_3$).

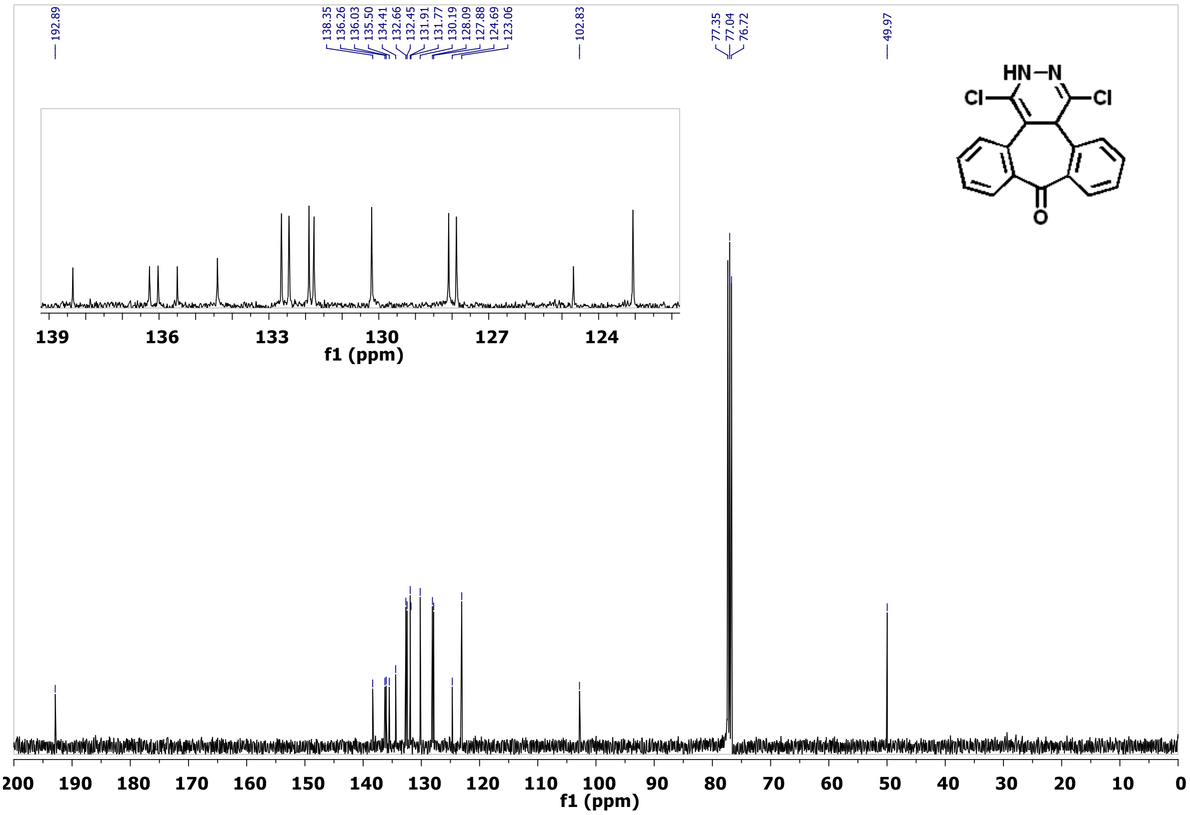

Fig. S14. ^{13}C-NMR spectrum of **3k** (100 MHz, $CDCl_3$).

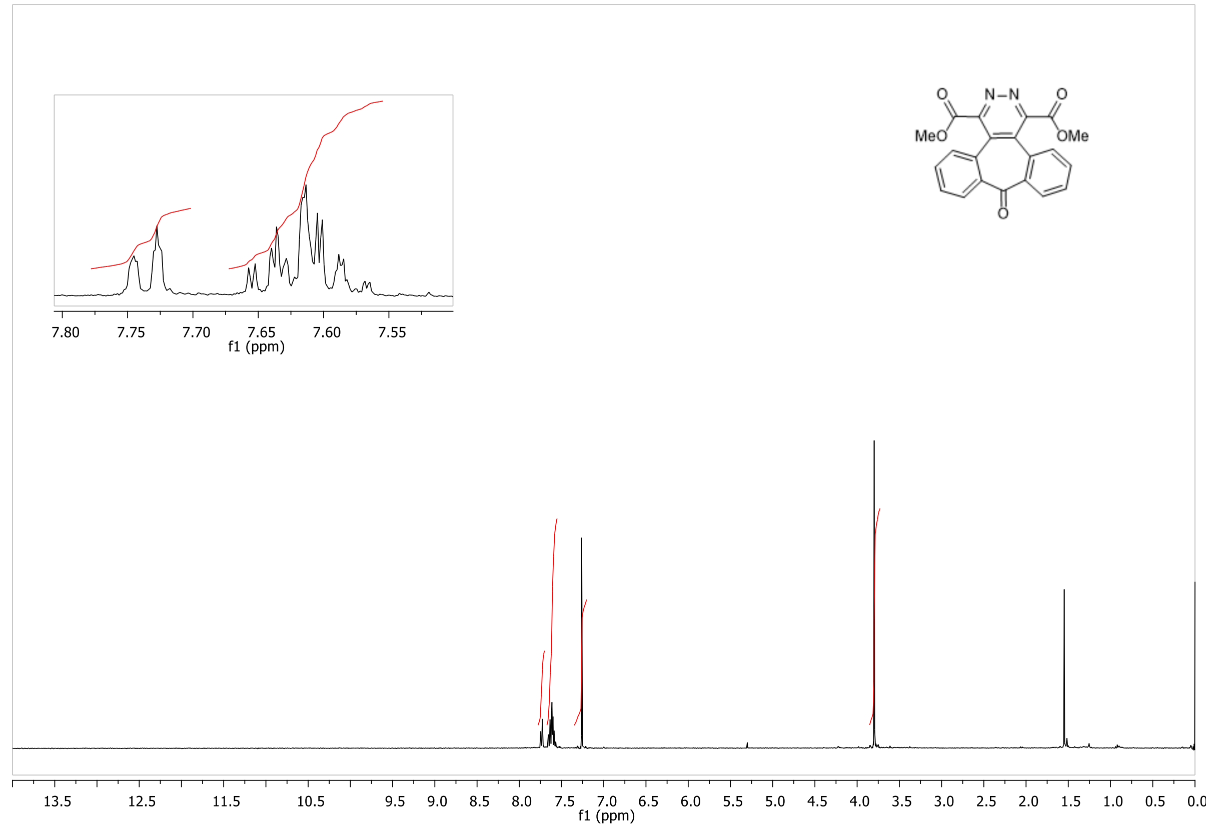

Fig. S15. ^{1}H-NMR spectrum of **4a** (400 MHz, $CDCl_3$).

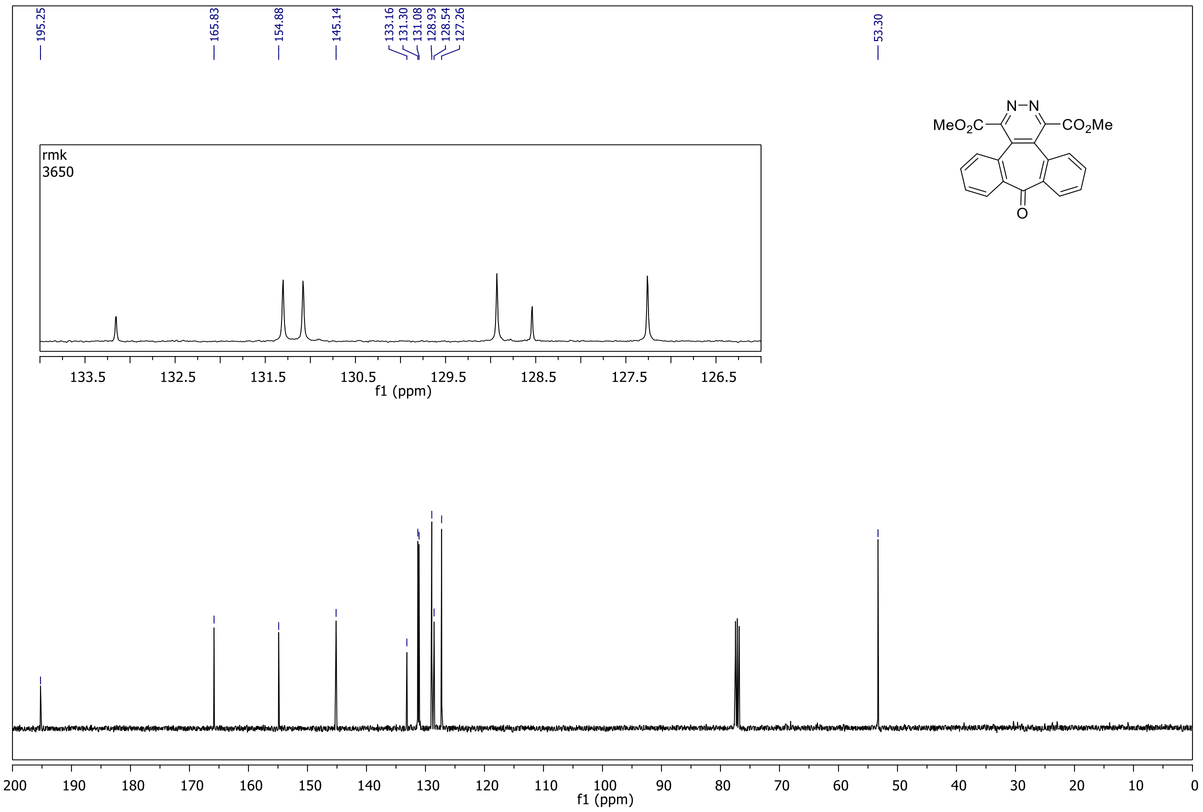

Fig. S16. ^{13}C-NMR spectrum of **4a** (100 MHz, $CDCl_3$).

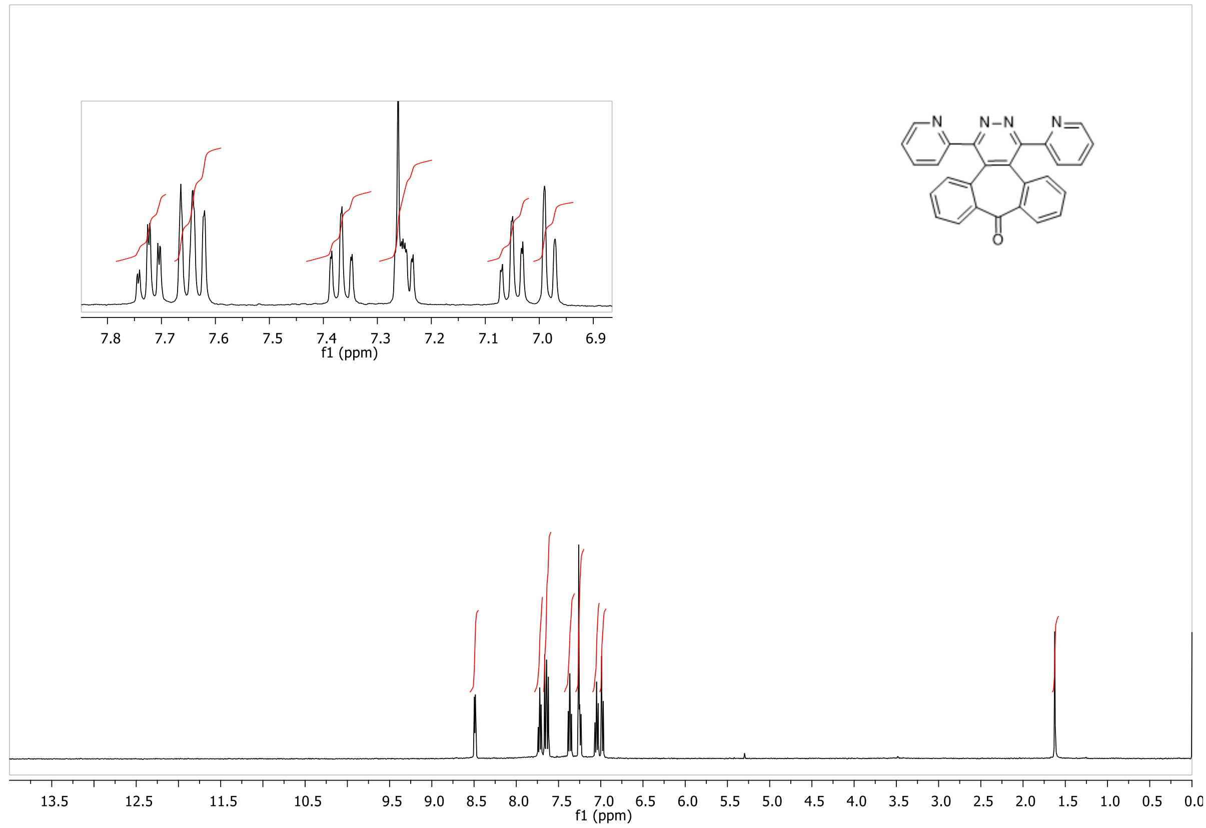

Fig. S17. ^{1}H-NMR spectrum of **4b** (400 MHz, $CDCl_3$).

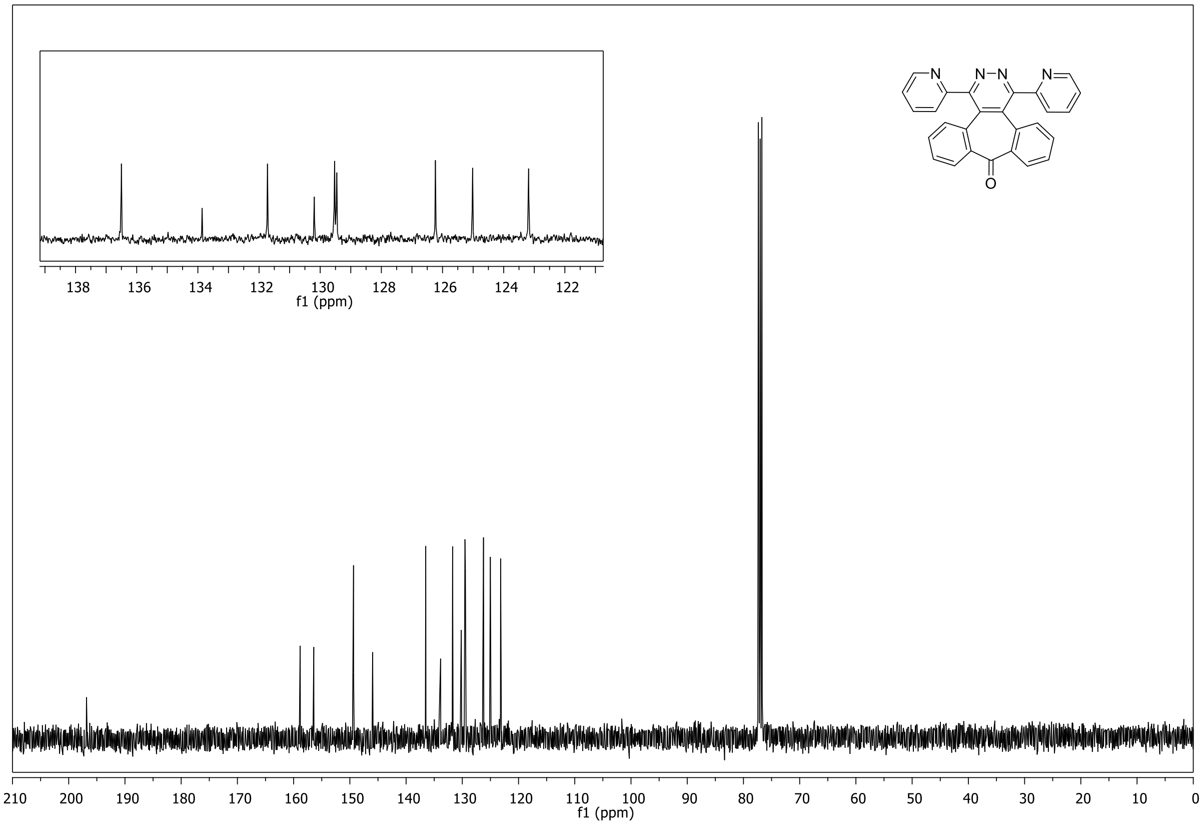

Fig. S18. ^{13}C-NMR spectrum of **4b** (100 MHz, $CDCl_3$).

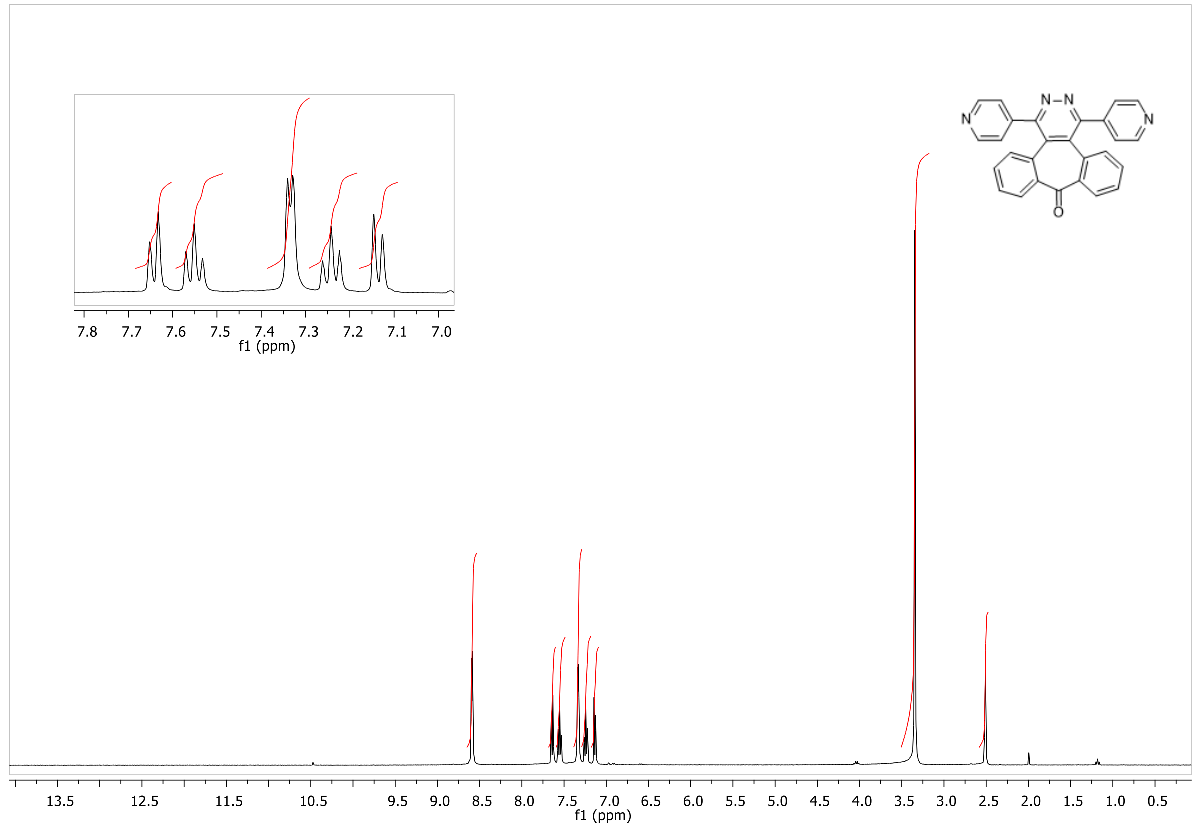

Fig. S19. ^{1}H-NMR spectrum of **4c** (400 MHz, DMSO-d_6).

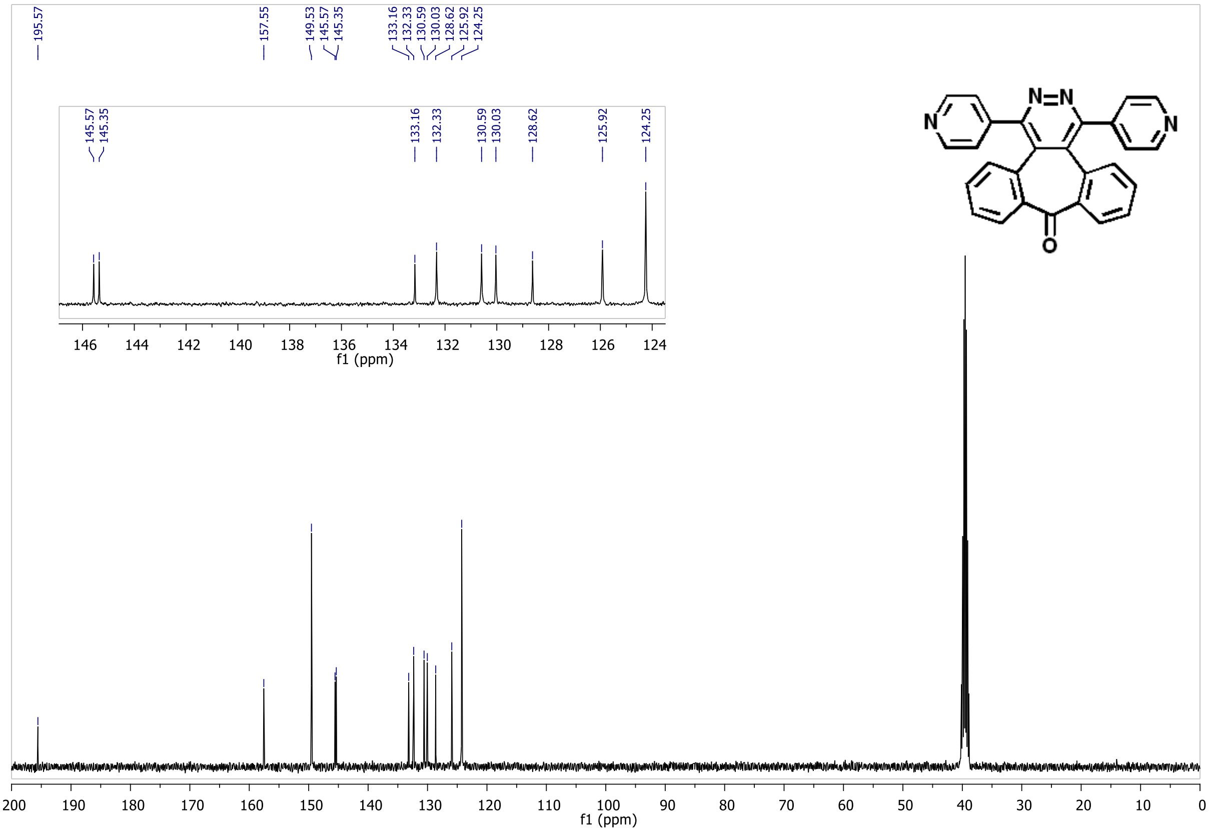

Fig. S20. ^{13}C-NMR spectrum of **4c** (100 MHz, DMSO-d_6).

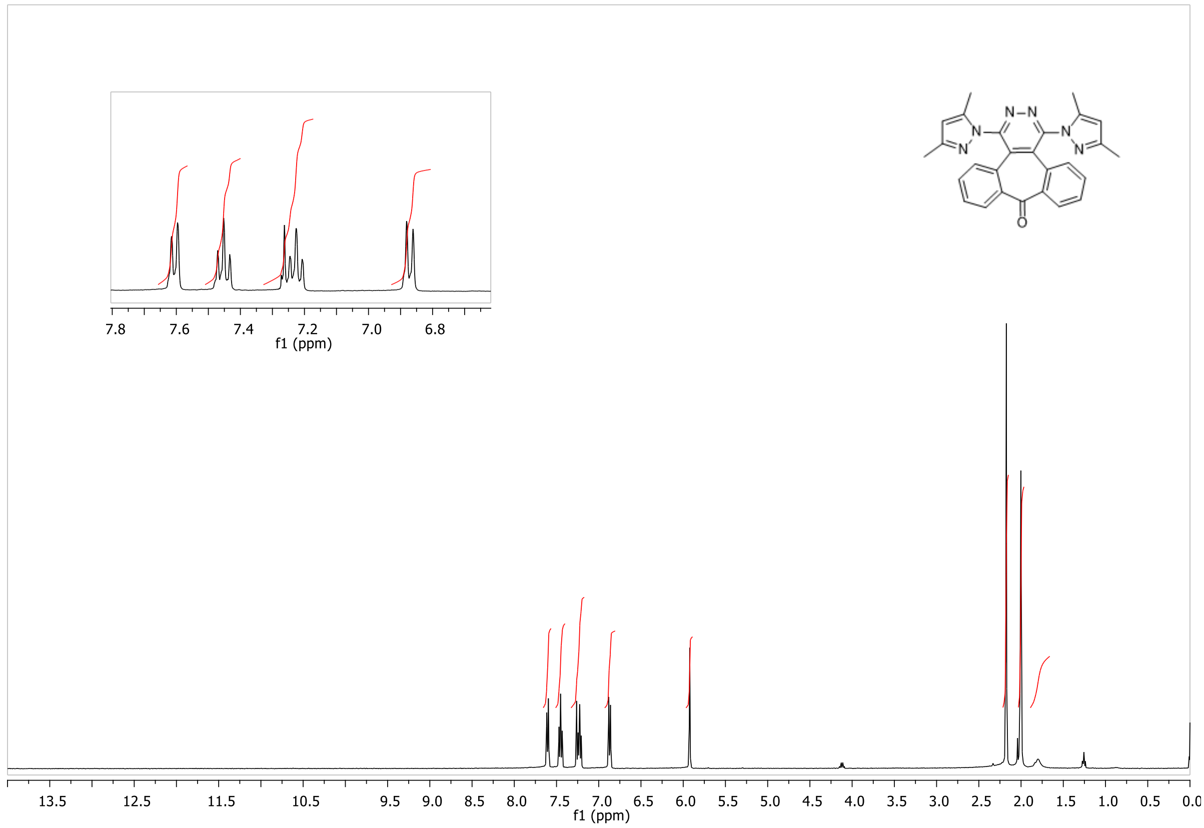

Fig. S21. ^{1}H-NMR spectrum of **4d** (400 MHz, $CDCl_3$).

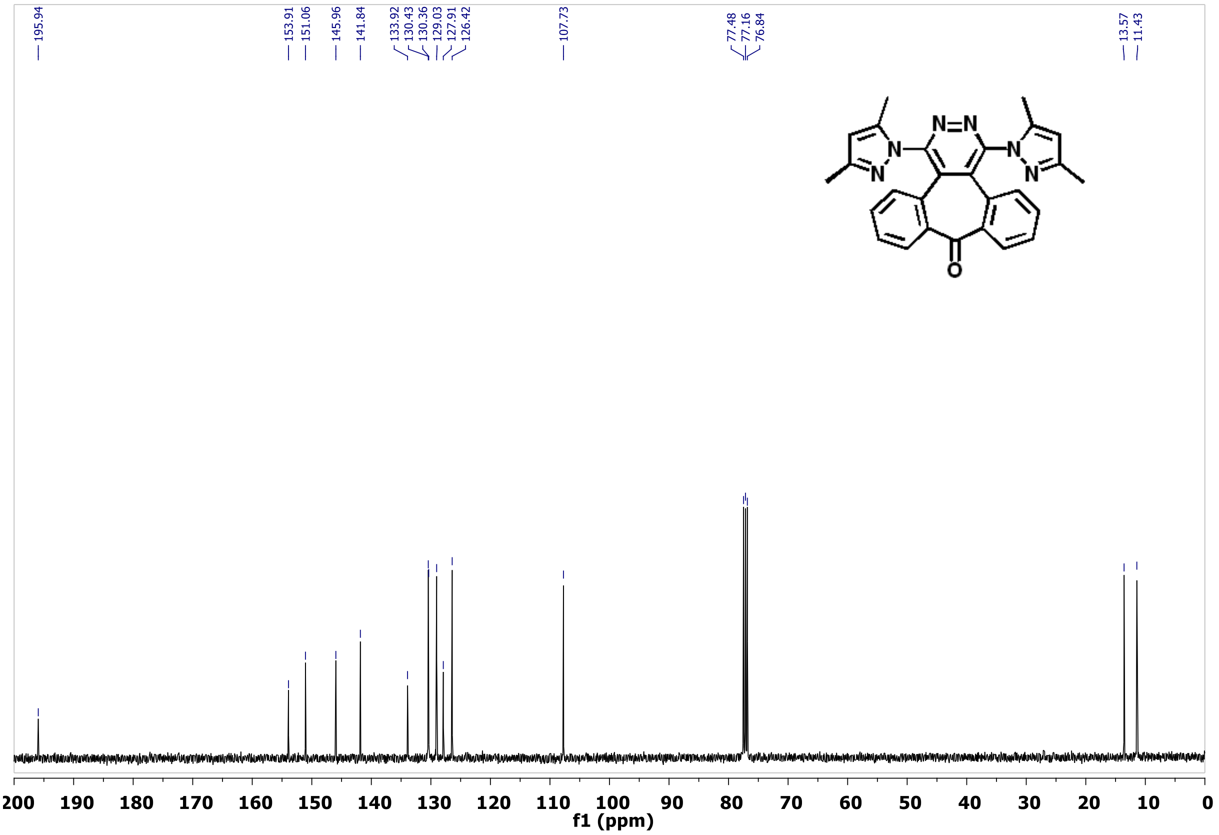

Fig. S22. ^{13}C-NMR spectrum of **4d** (100 MHz, $CDCl_3$).

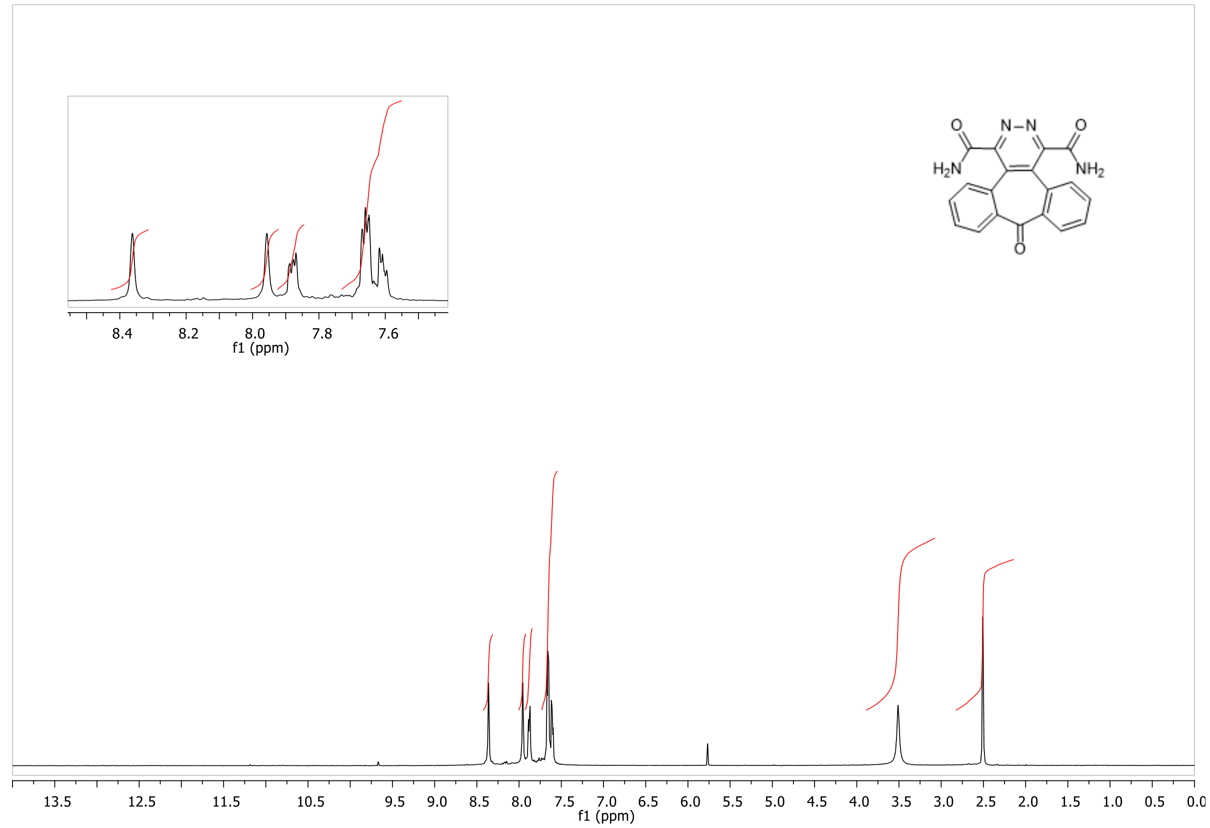

Fig. S23. ^{1}H-NMR spectrum of **4e** (400 MHz, DMSO-d_6).

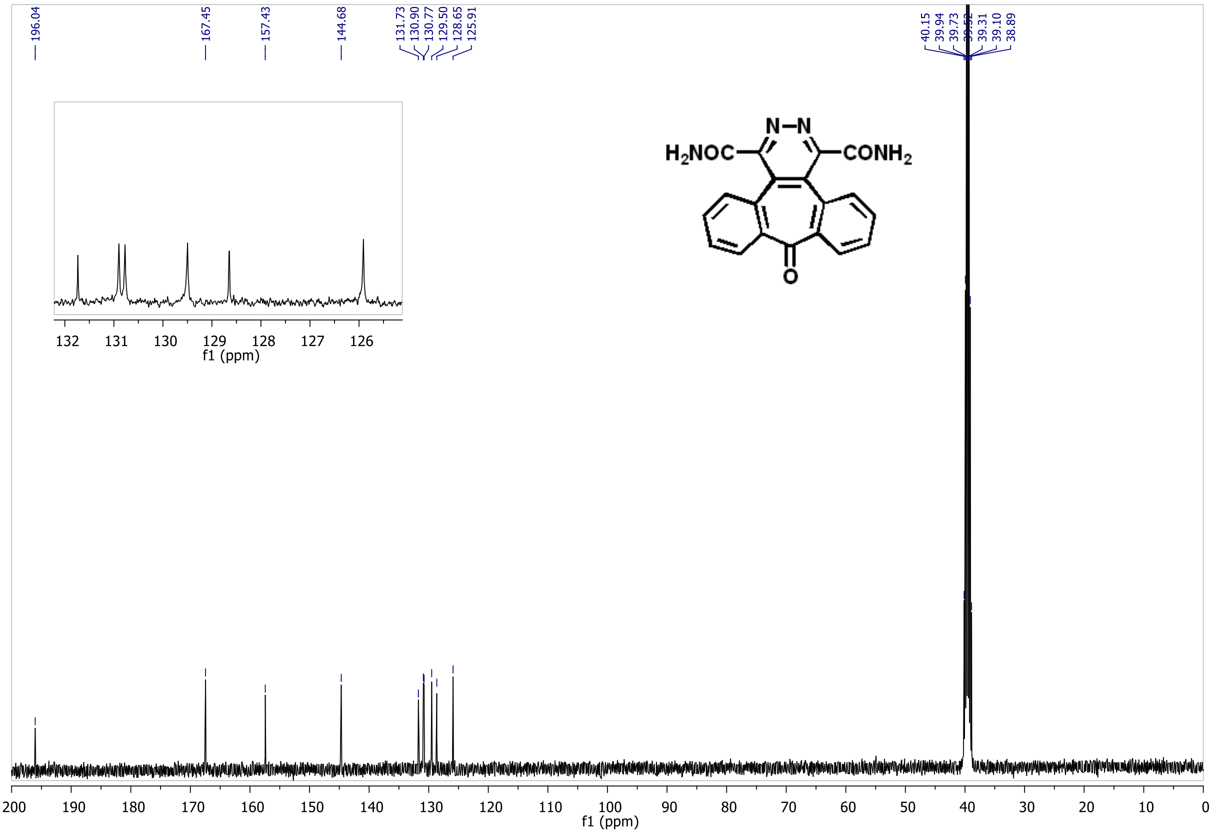

Fig. S24. ^{13}C-NMR spectrum of **4e** (100 MHz, DMSO-*d*6).

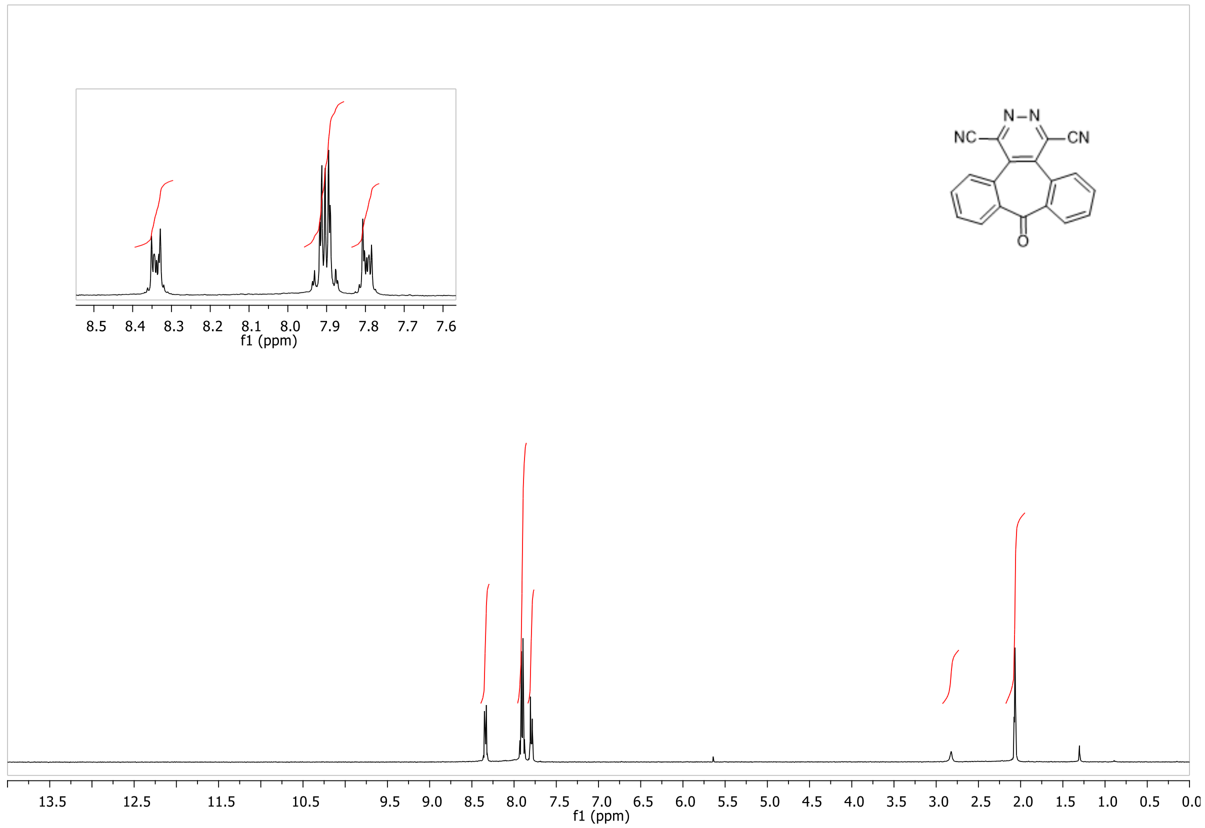

Fig. S25. ^{1}H-NMR spectrum of **4f** (400 MHz, DMSO-*d*6).

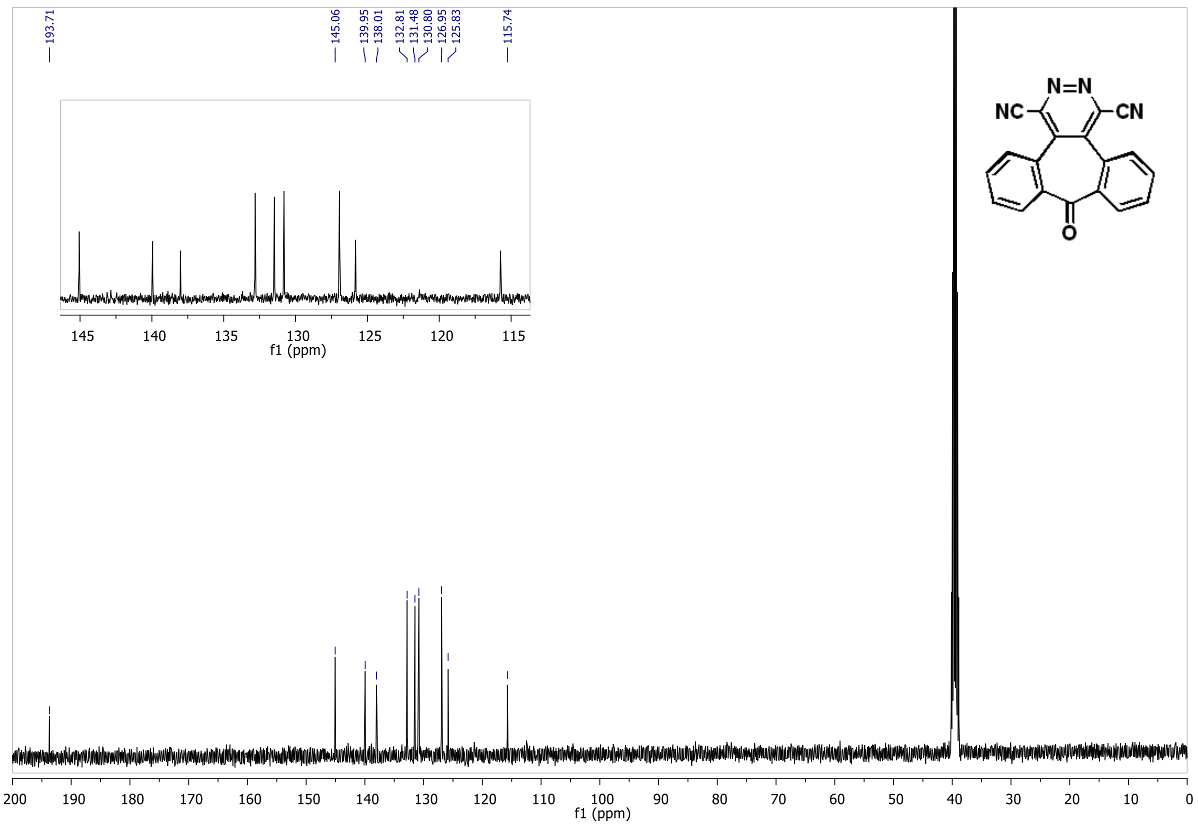

Fig. S26. ^{13}C-NMR spectrum of **4f** (100 MHz, DMSO-d_6).

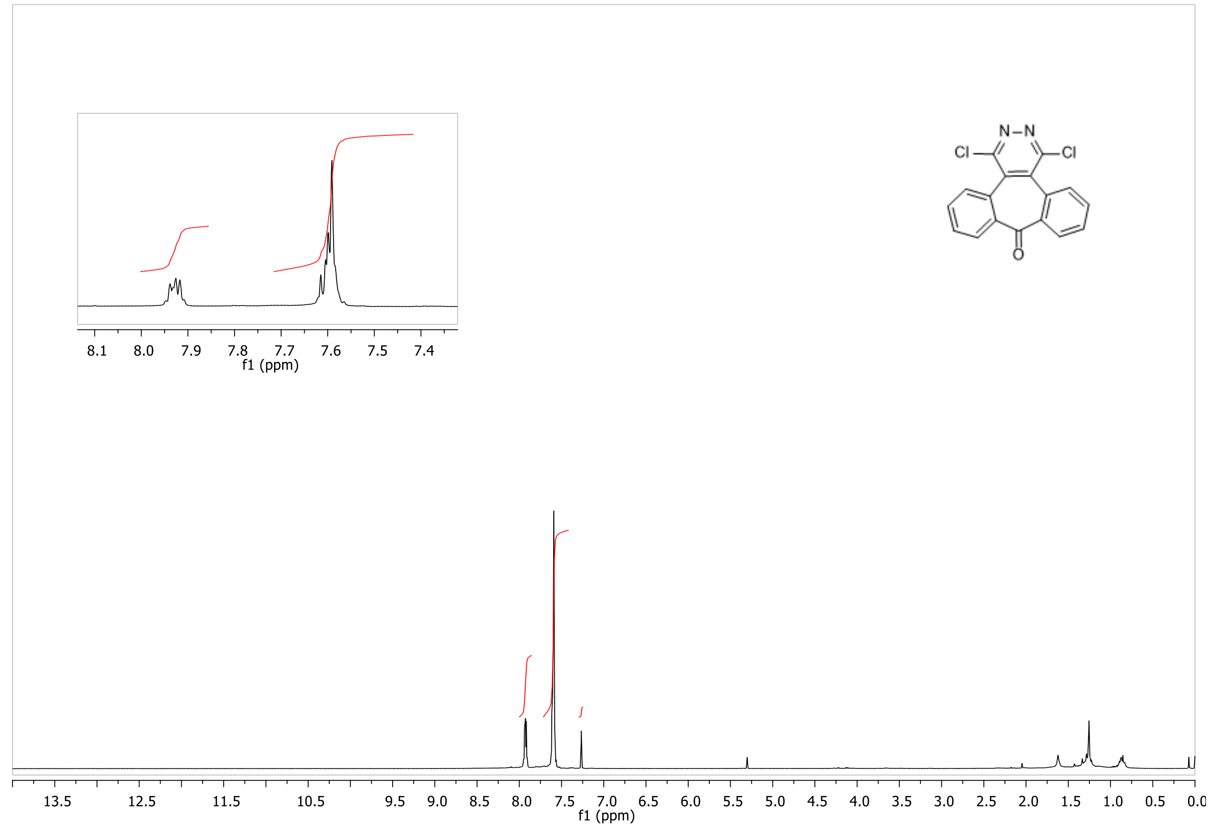

Fig. S27. ^{1}H-NMR spectrum of **4k** (400 MHz, $CDCl_3$).

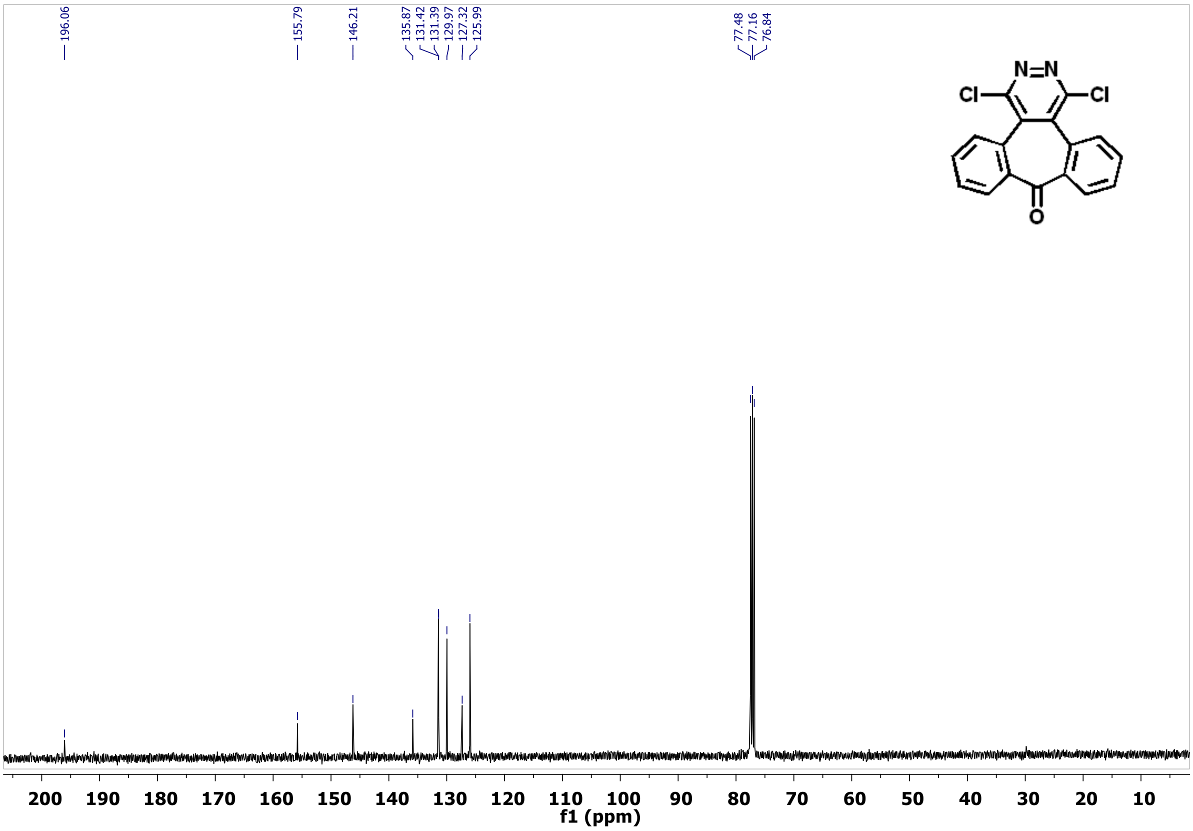

Fig. S28. ^{13}C-NMR spectrum of **4k** (100 MHz, $CDCl_3$).

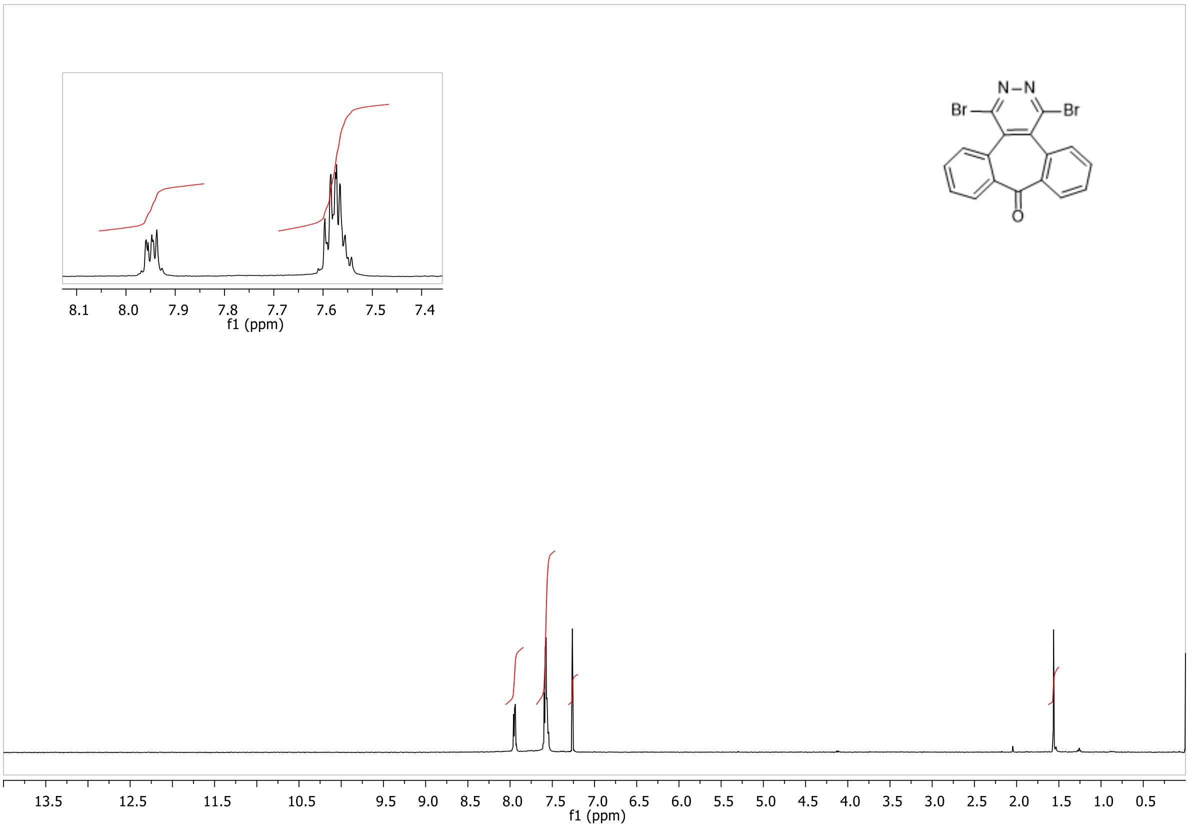

Fig. S29. ^{1}H-NMR spectrum of **4l** (400 MHz, $CDCl_3$).

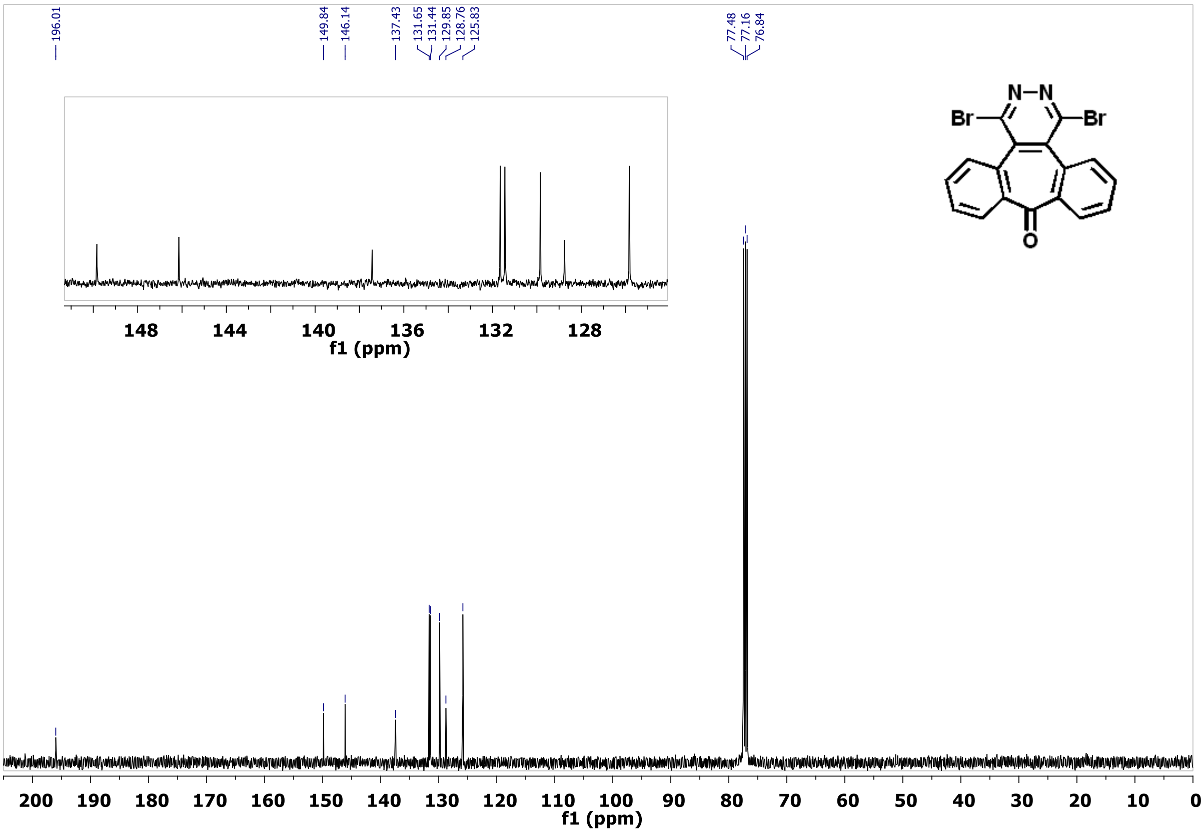

Fig. S30. ^{13}C-NMR spectrum of **4l** (100 MHz, $CDCl_3$).

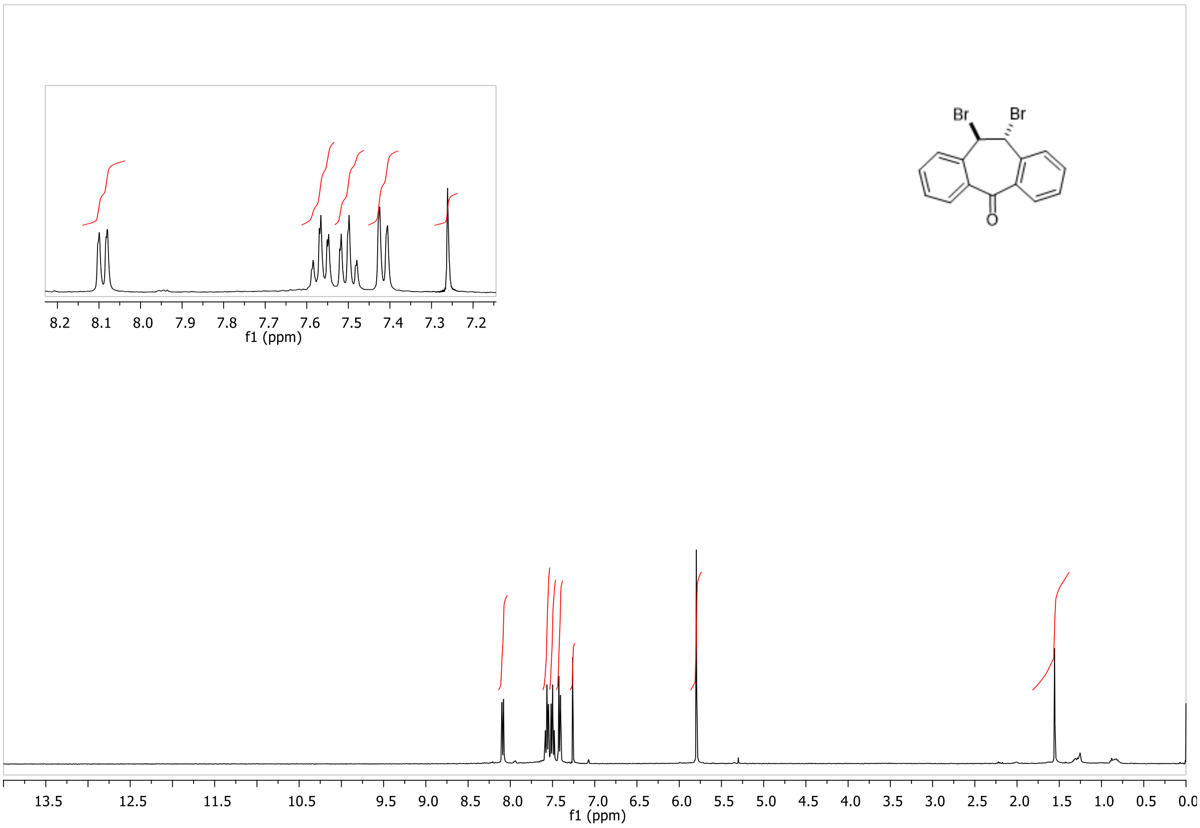

Fig. S31. ^{1}H-NMR spectrum of **5l** (400 MHz, $CDCl_3$).

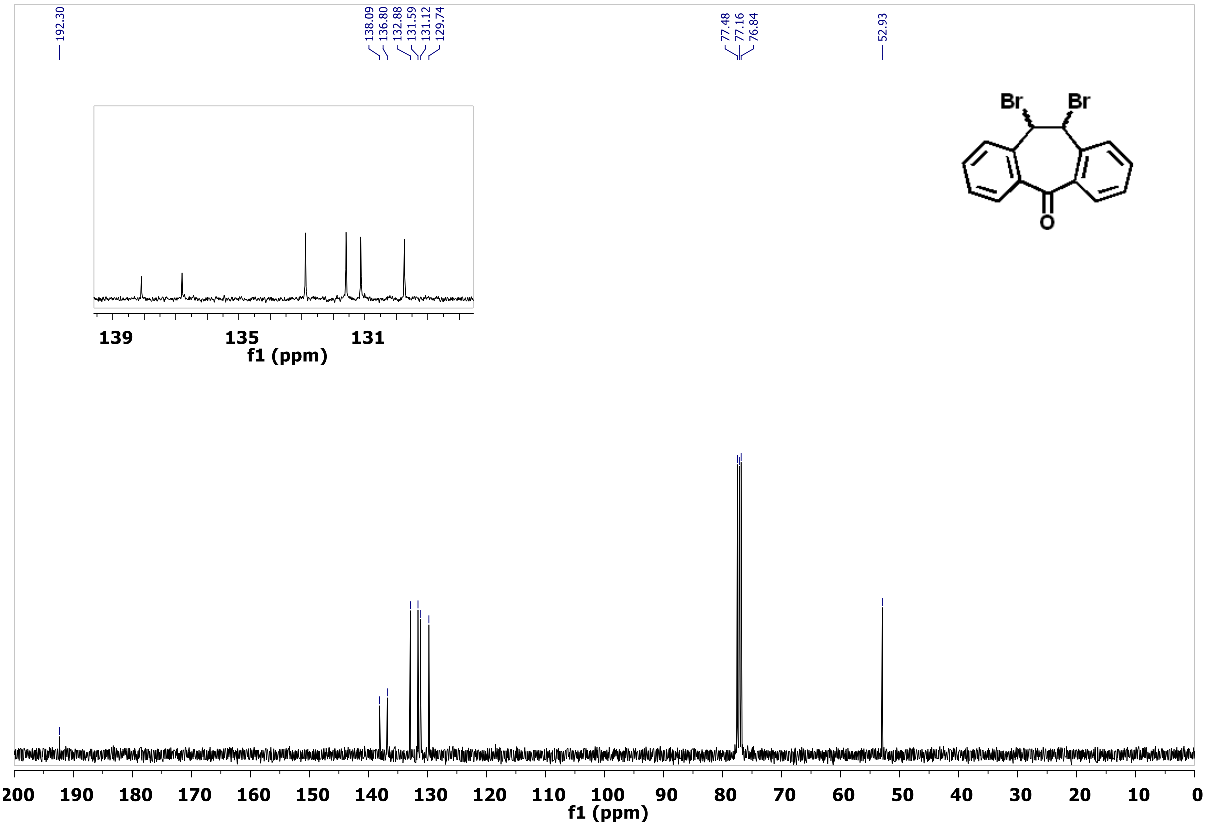

Fig. S32. ^{13}C-NMR spectrum of **5l** (100 MHz, $CDCl_3$).

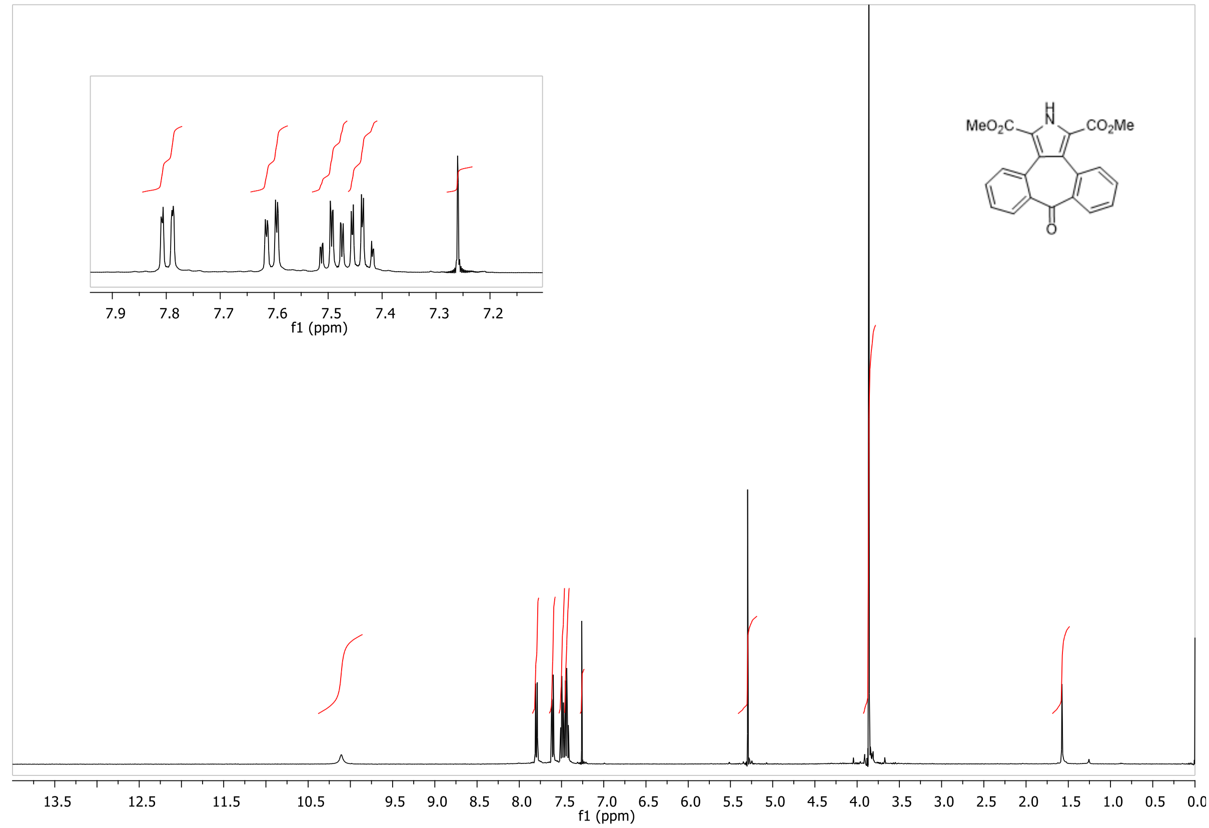

Fig. S33. ^{1}H-NMR spectrum of **10aa** (400 MHz, $CDCl_3$).

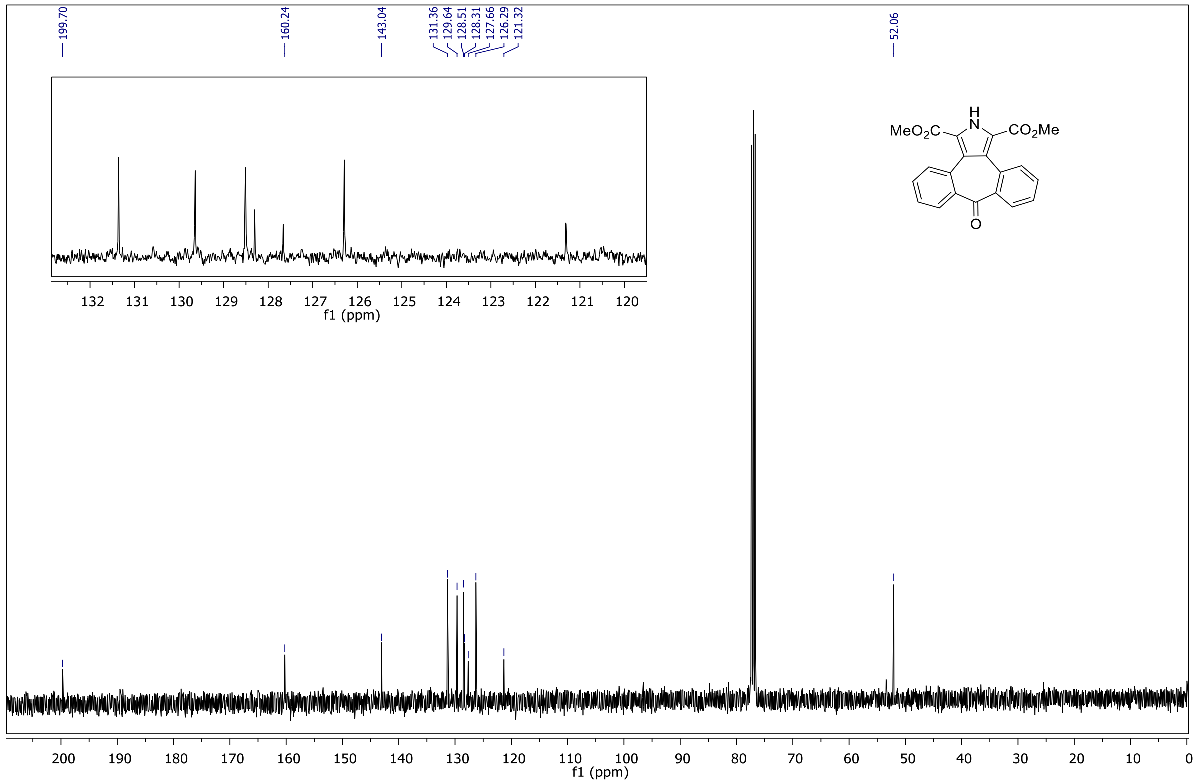

Fig. S34. ^{13}C-NMR spectrum of **10aa** (100 MHz, $CDCl_3$).

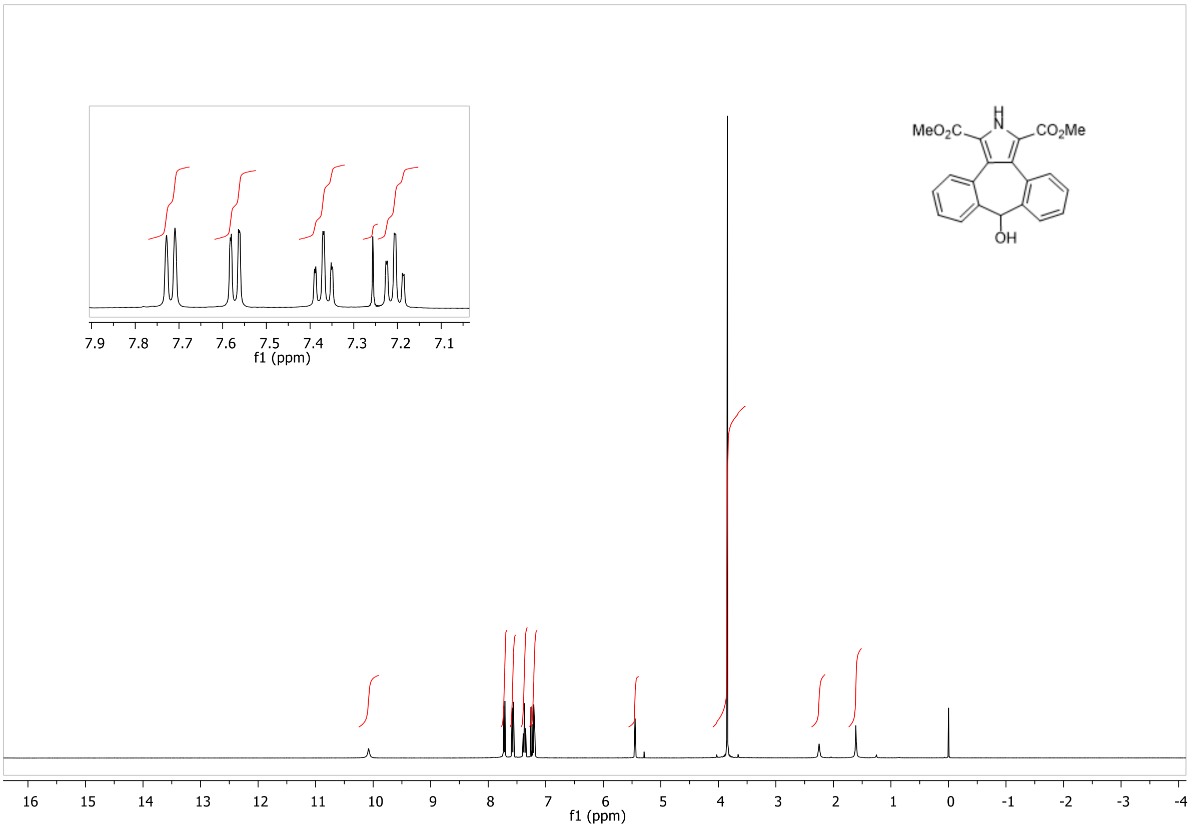

Fig. S35. ^{1}H-NMR spectrum of **10ab** (400 MHz, $CDCl_3$).

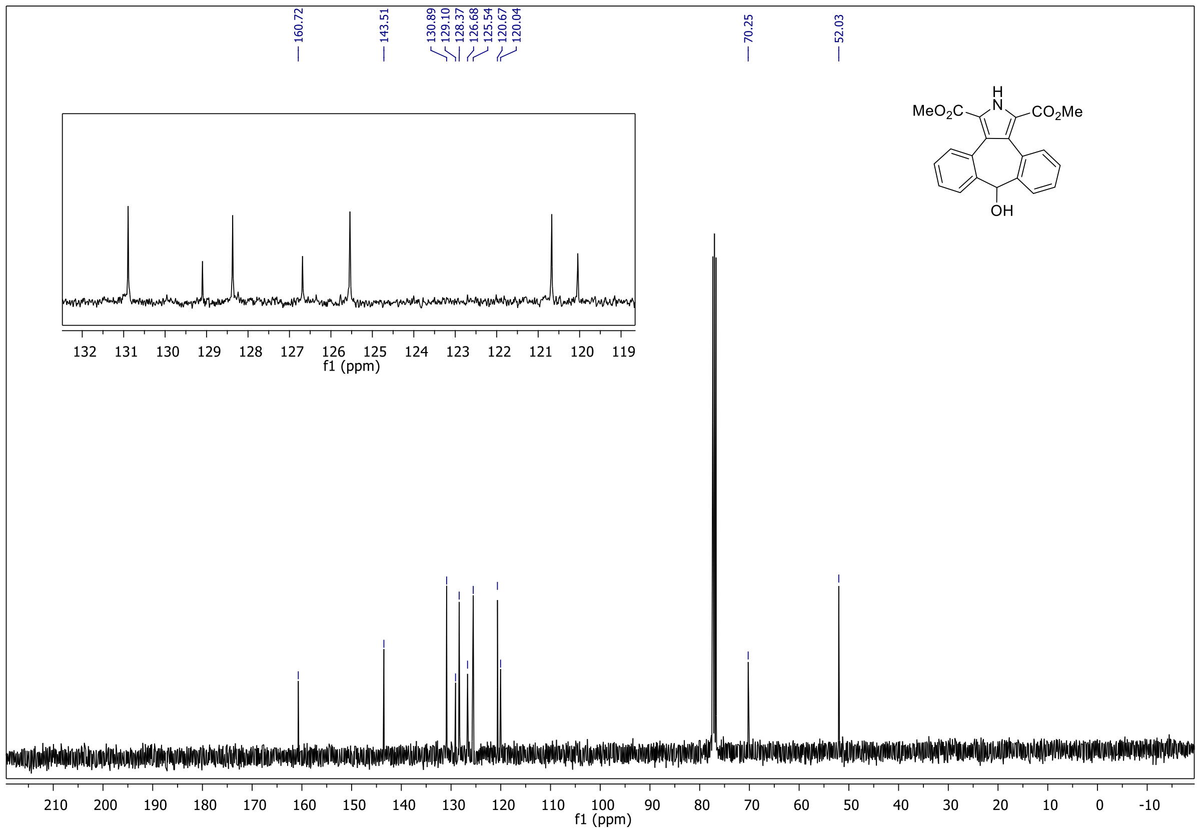

Fig. S36. ^{13}C-NMR spectrum of **10ab** (100 MHz, $CDCl_3$).

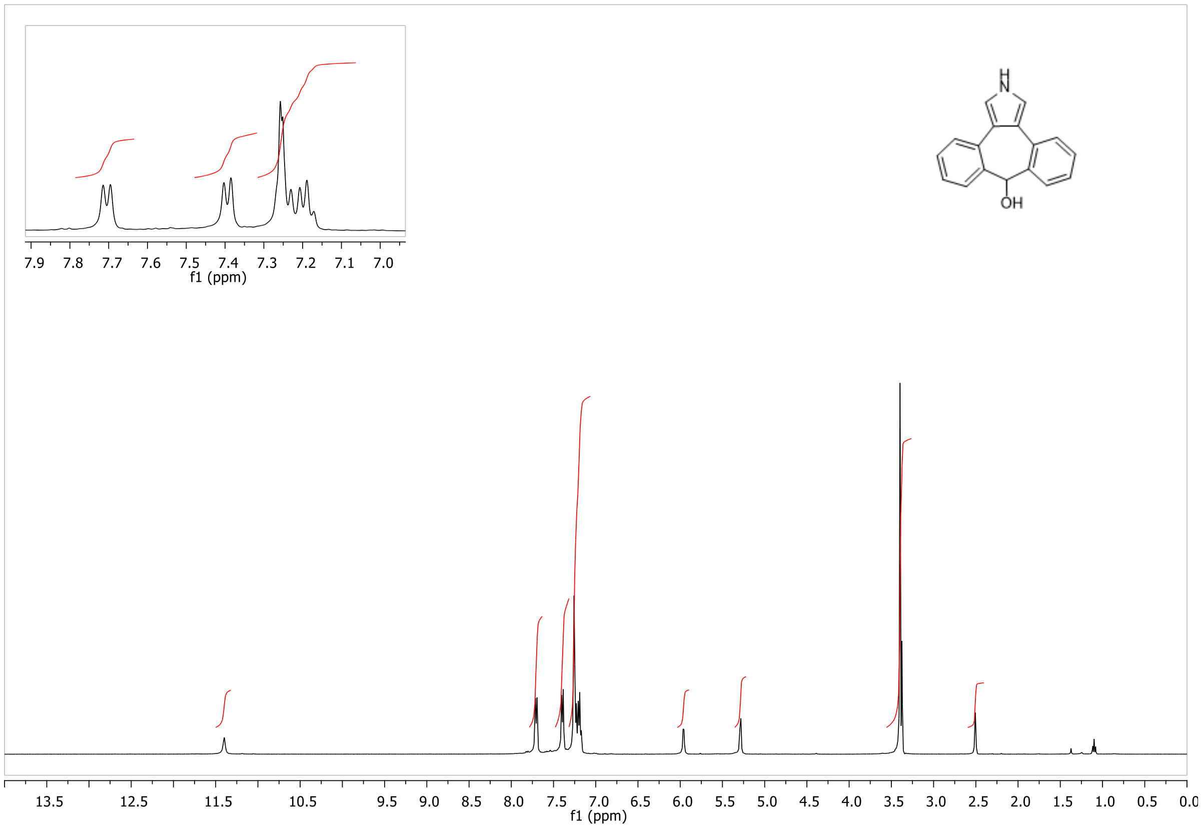

Fig. S37. ^{1}H-NMR spectrum of **10ac** (400 MHz, DMSO-d_6).

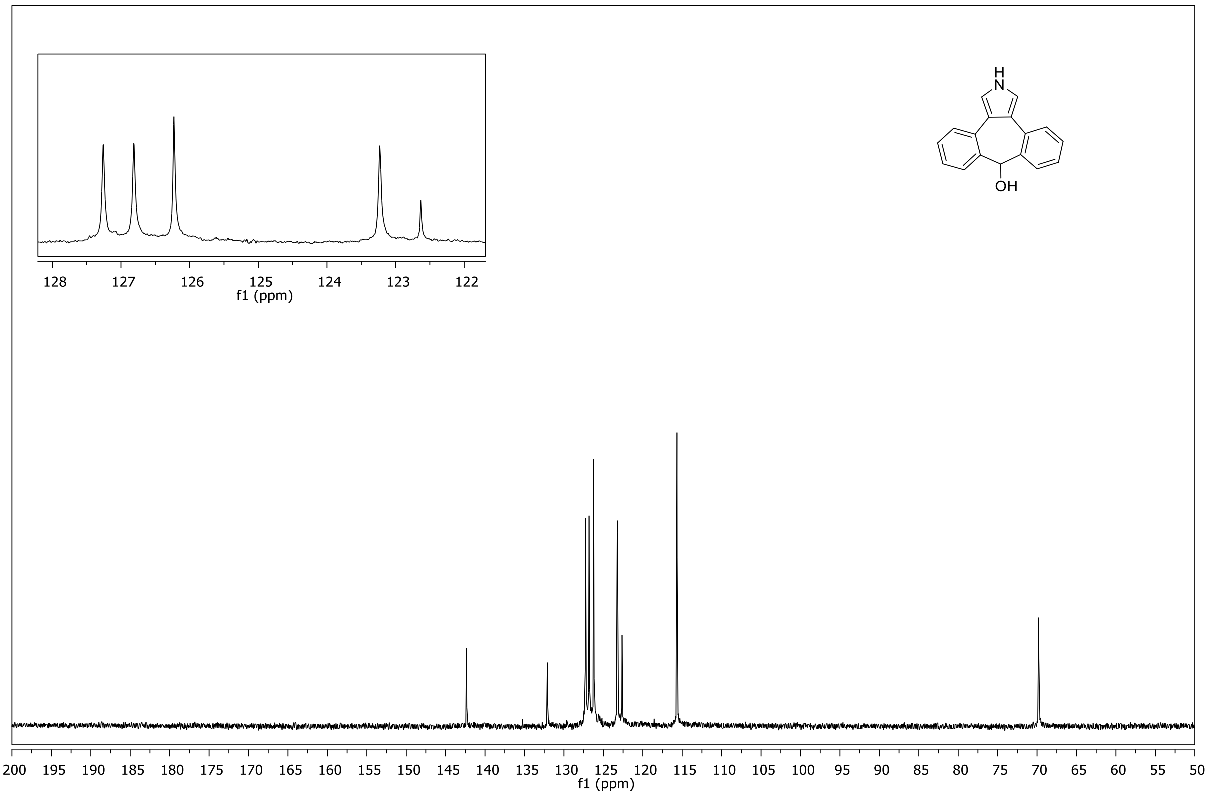

Fig. S38. ^{13}C-NMR spectrum of **10ac** (100 MHz, DMSO-*d*$_6$).

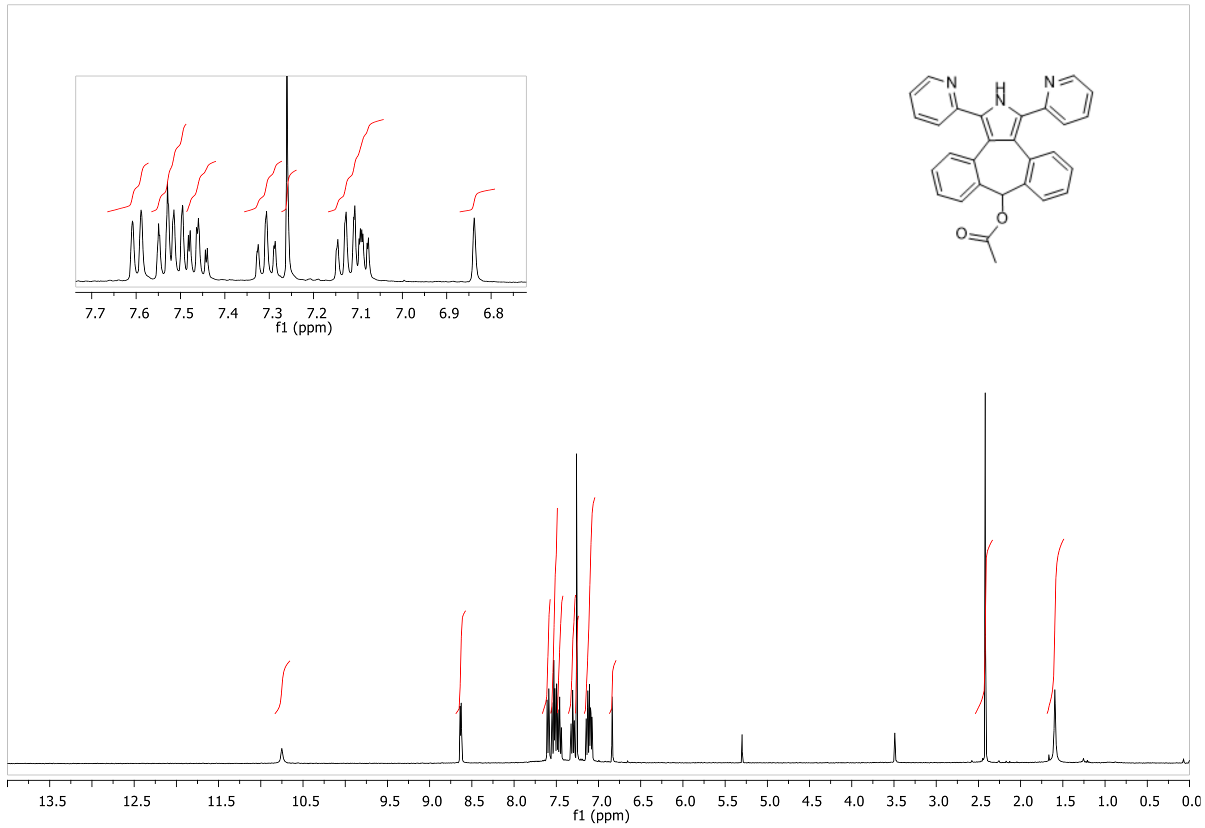

Fig. S39. ^{1}H-NMR spectrum of **10ba** (400 MHz, $CDCl_3$).

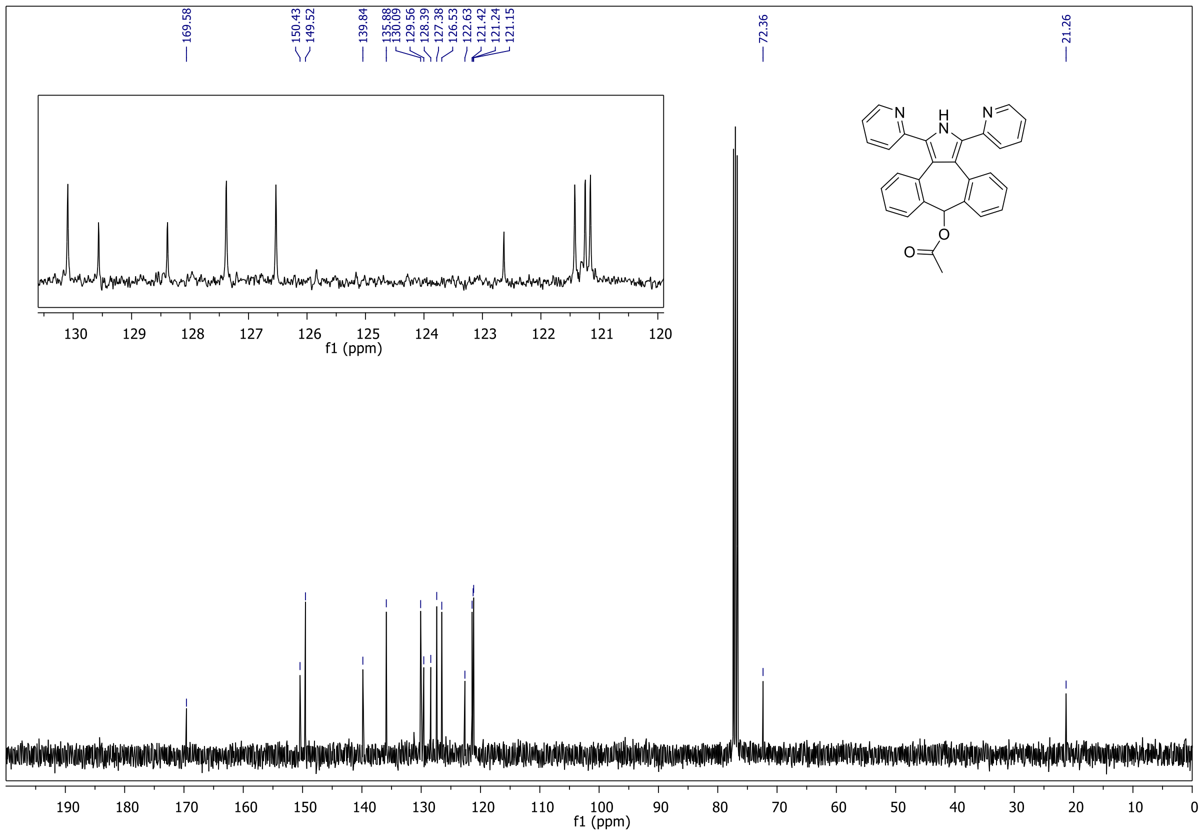

Fig. S40. ^{13}C-NMR spectrum of **10ba** (100 MHz, $CDCl_3$).

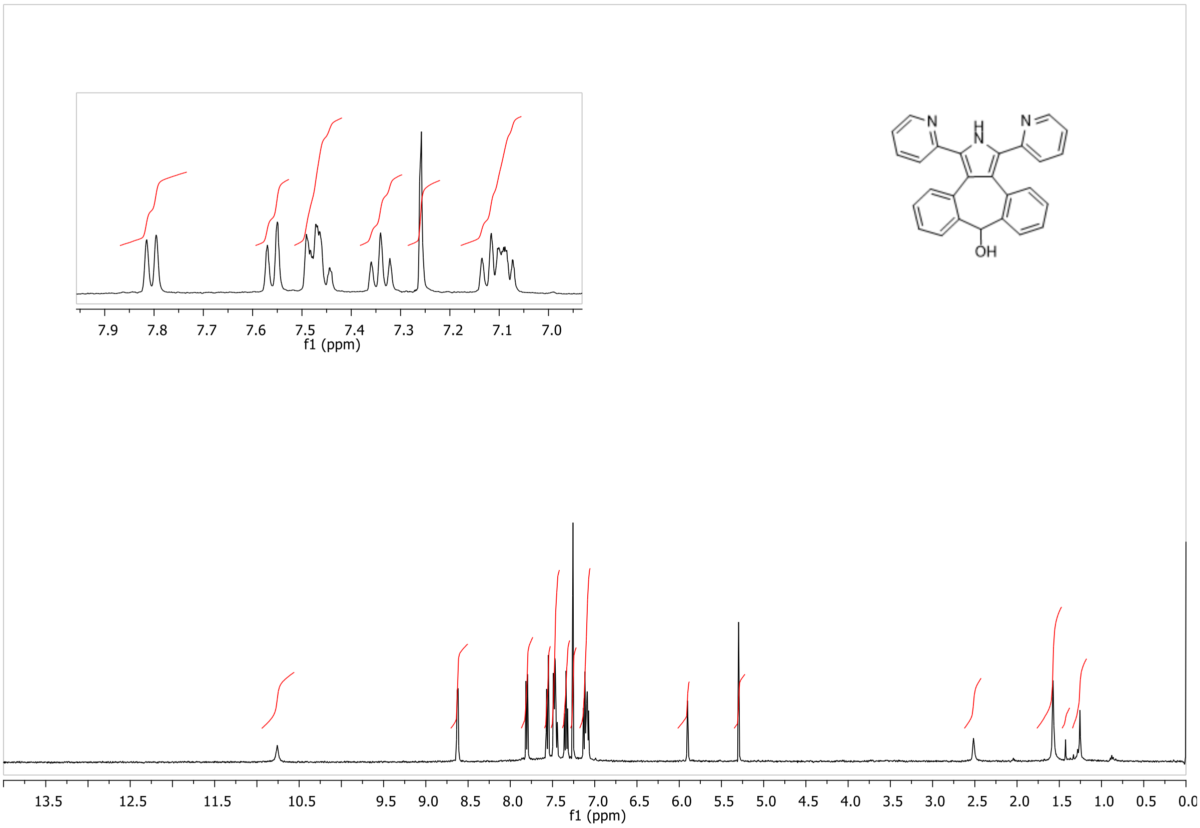

Fig. S41. ^{1}H-NMR spectrum of **10bb** (400 MHz, $CDCl_3$).

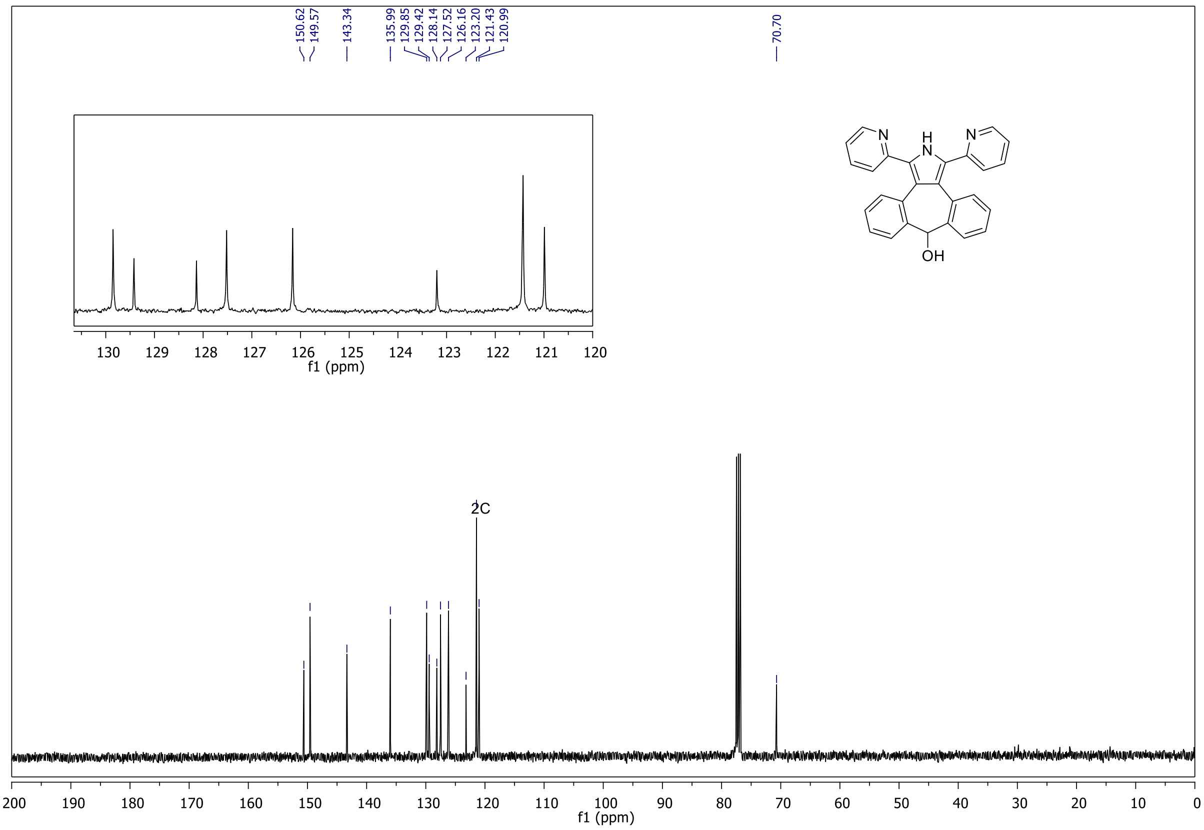

Fig. S42. ^{13}C-NMR spectrum of **10bb** (100 MHz, $CDCl_3$).

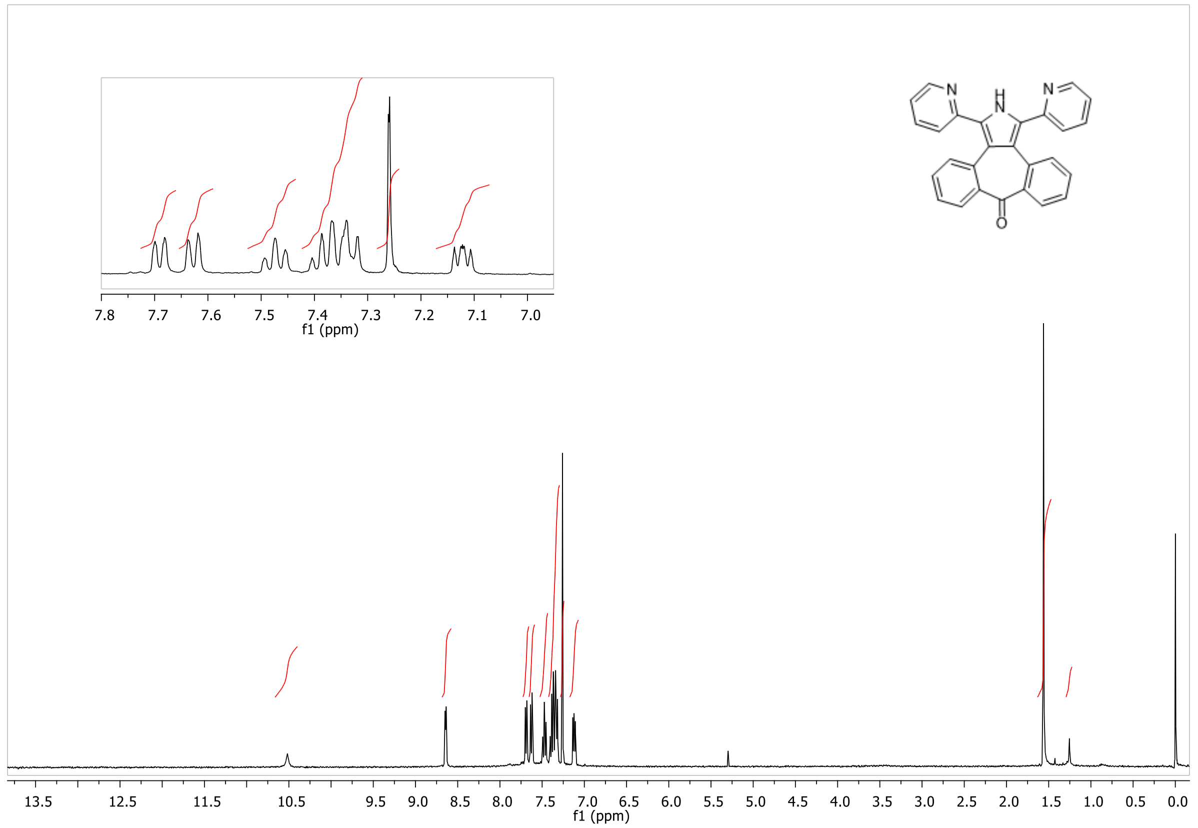

Fig. S43. ^{1}H-NMR spectrum of **10bc** (400 MHz, $CDCl_3$).

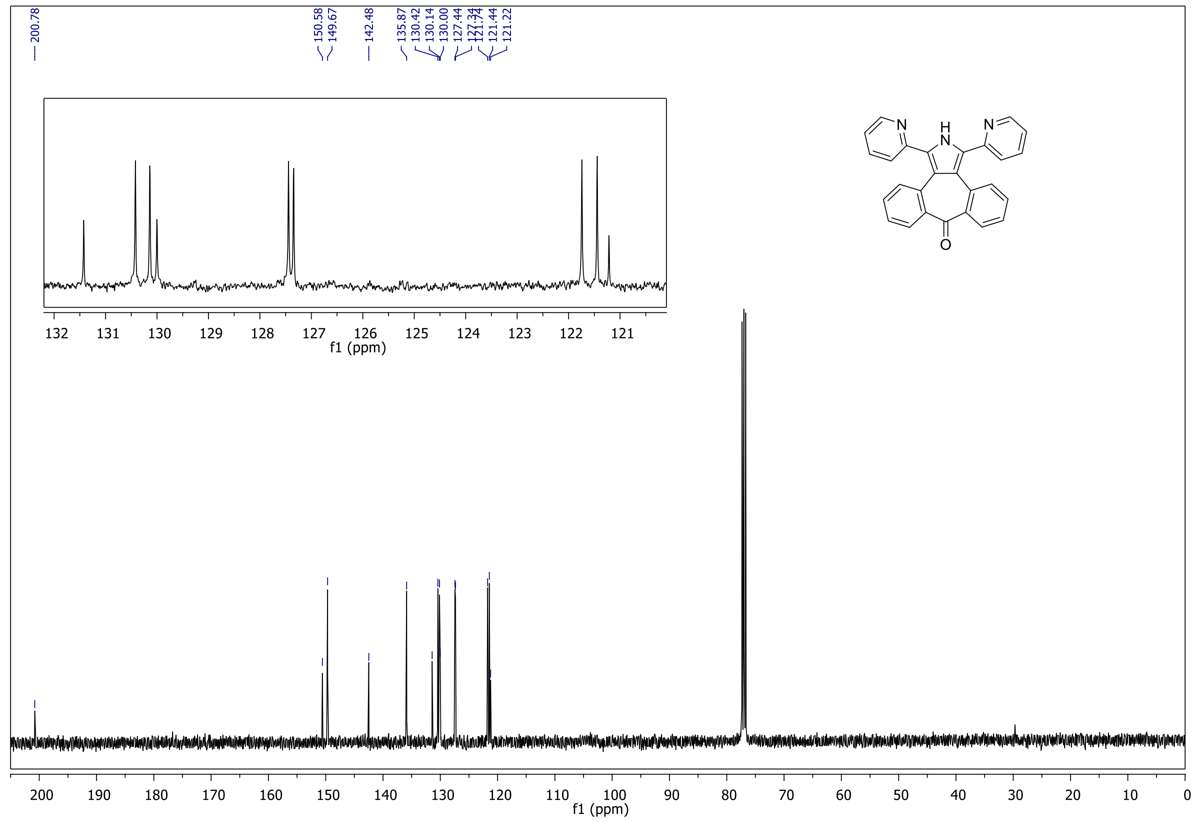

Fig. S44. ^{13}C-NMR spectrum of **10bc** (100 MHz, $CDCl_3$).

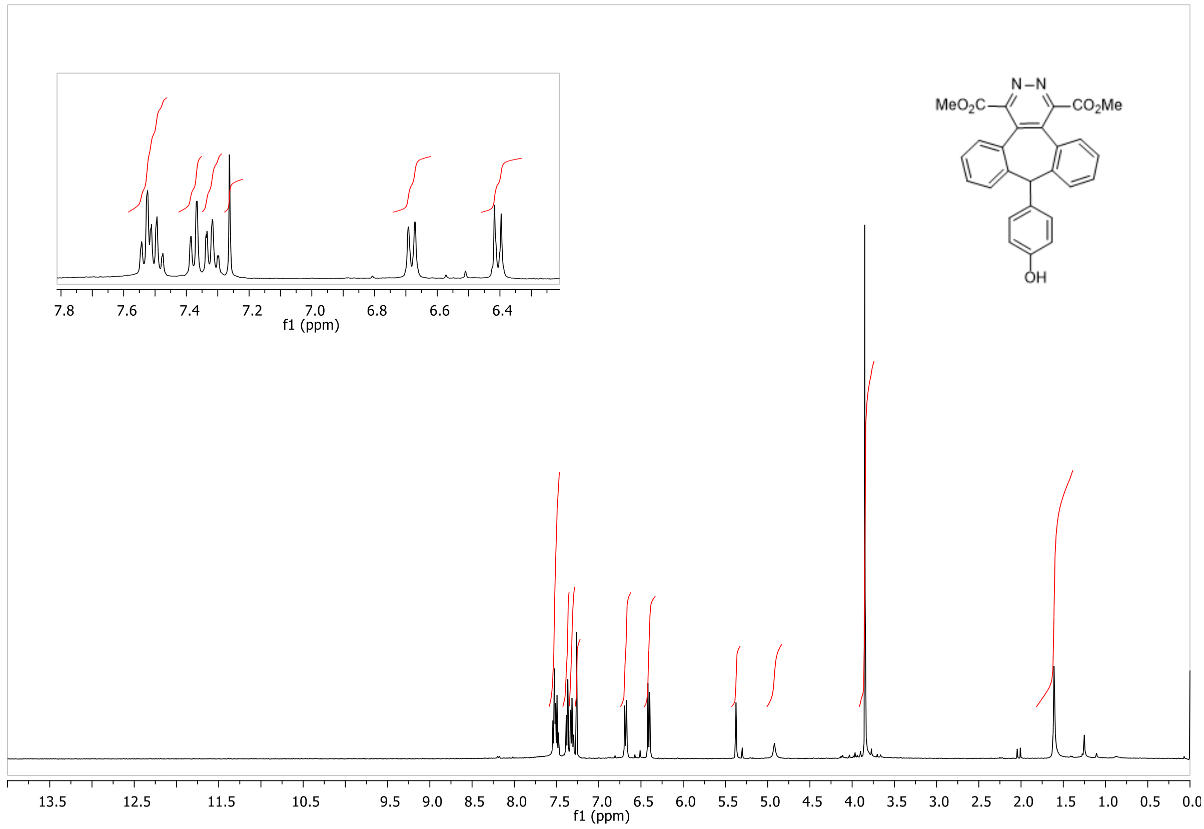

Fig. S45. ^{1}H-NMR spectrum of **13a** (400 MHz, $CDCl_3$).

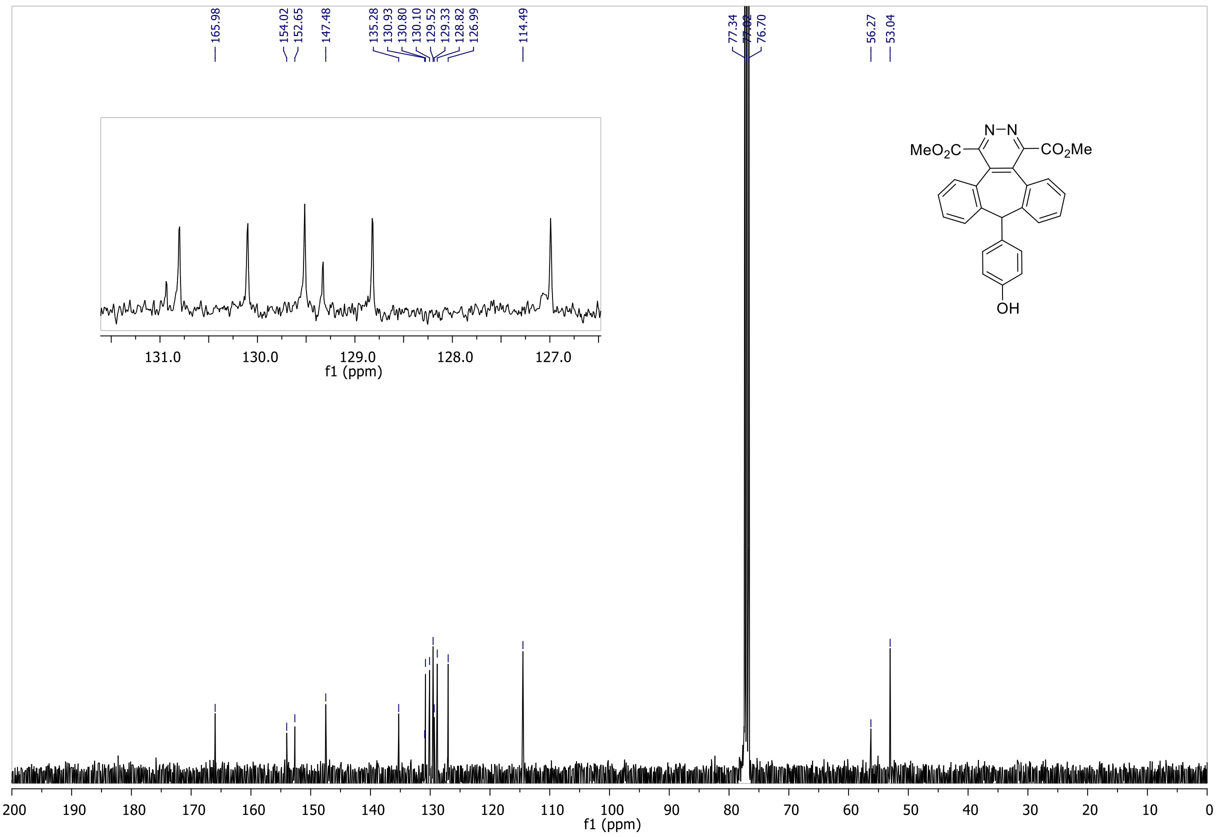

Fig. S46. ^{13}C-NMR spectrum of **13a** (100 MHz, $CDCl_3$).

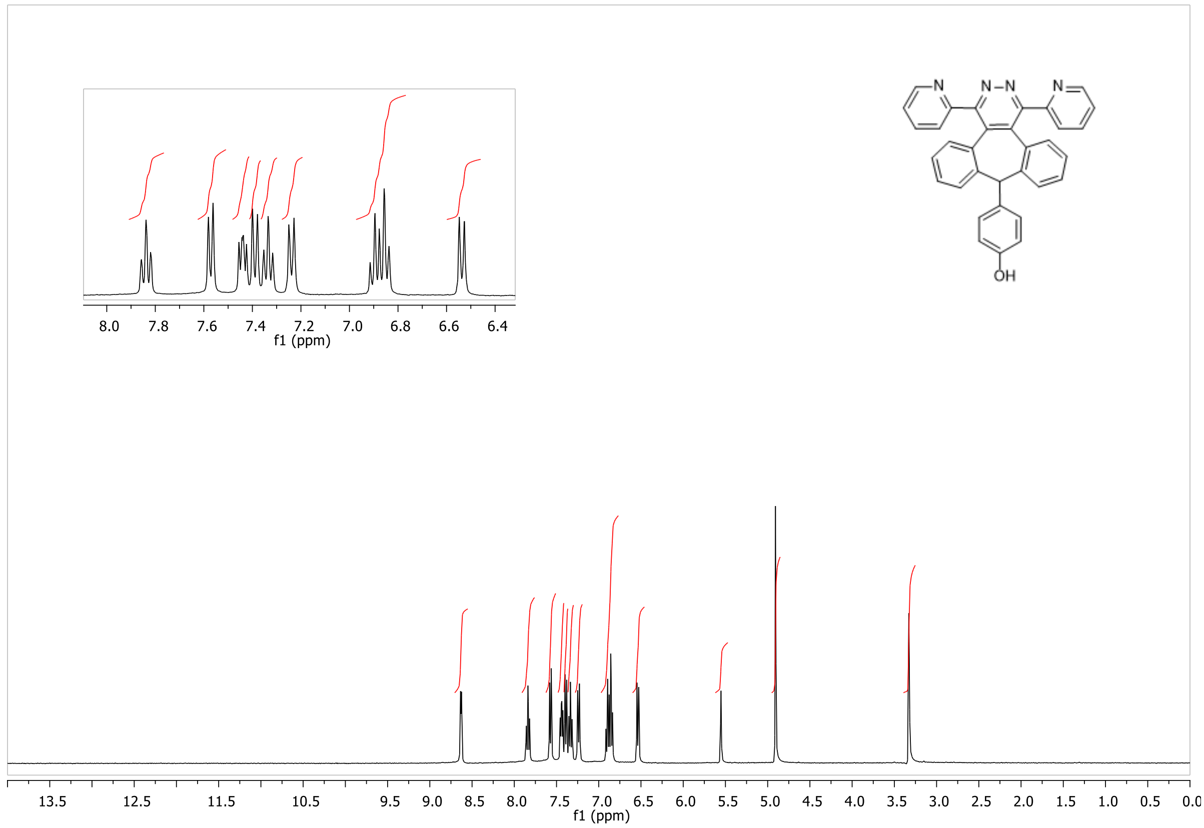

Fig. S47. ^{1}H-NMR spectrum of **13b** (400 MHz, MeOD).

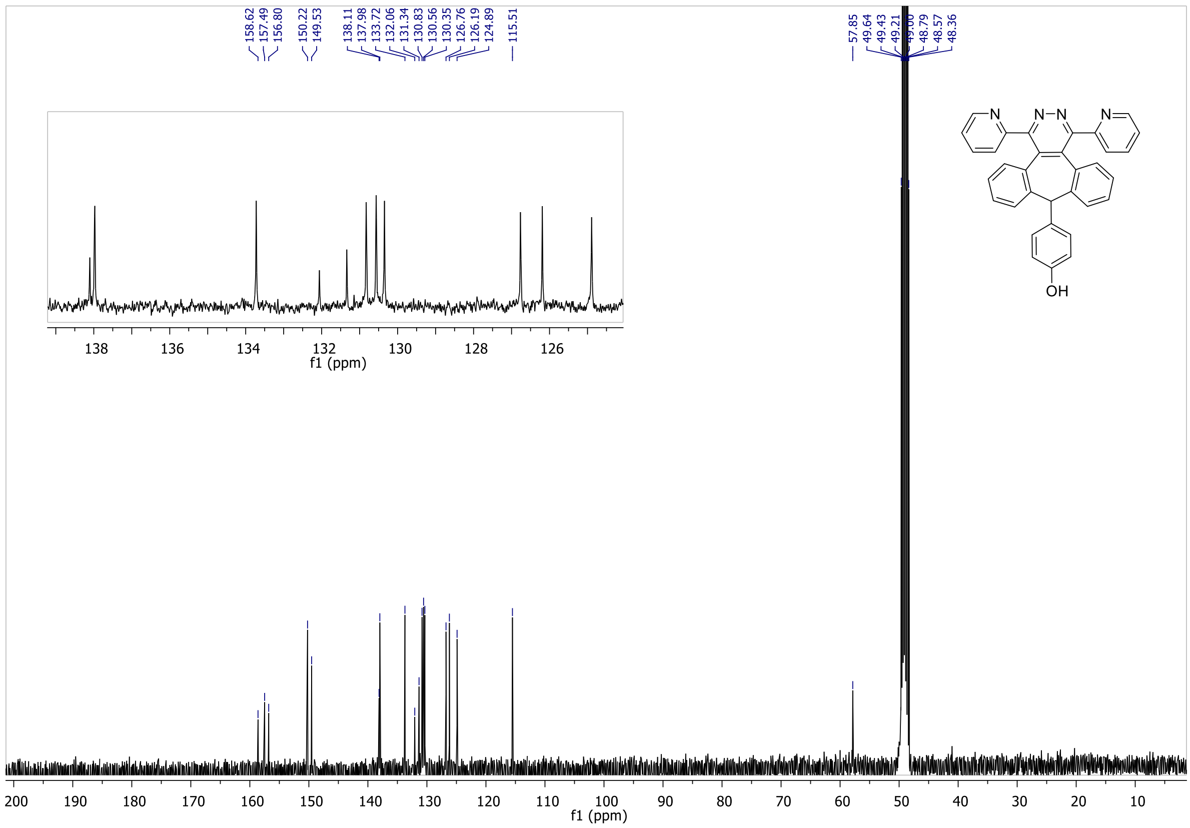

Fig. S48. ^{13}C-NMR spectrum of **13b** (100 MHz, MeOD).

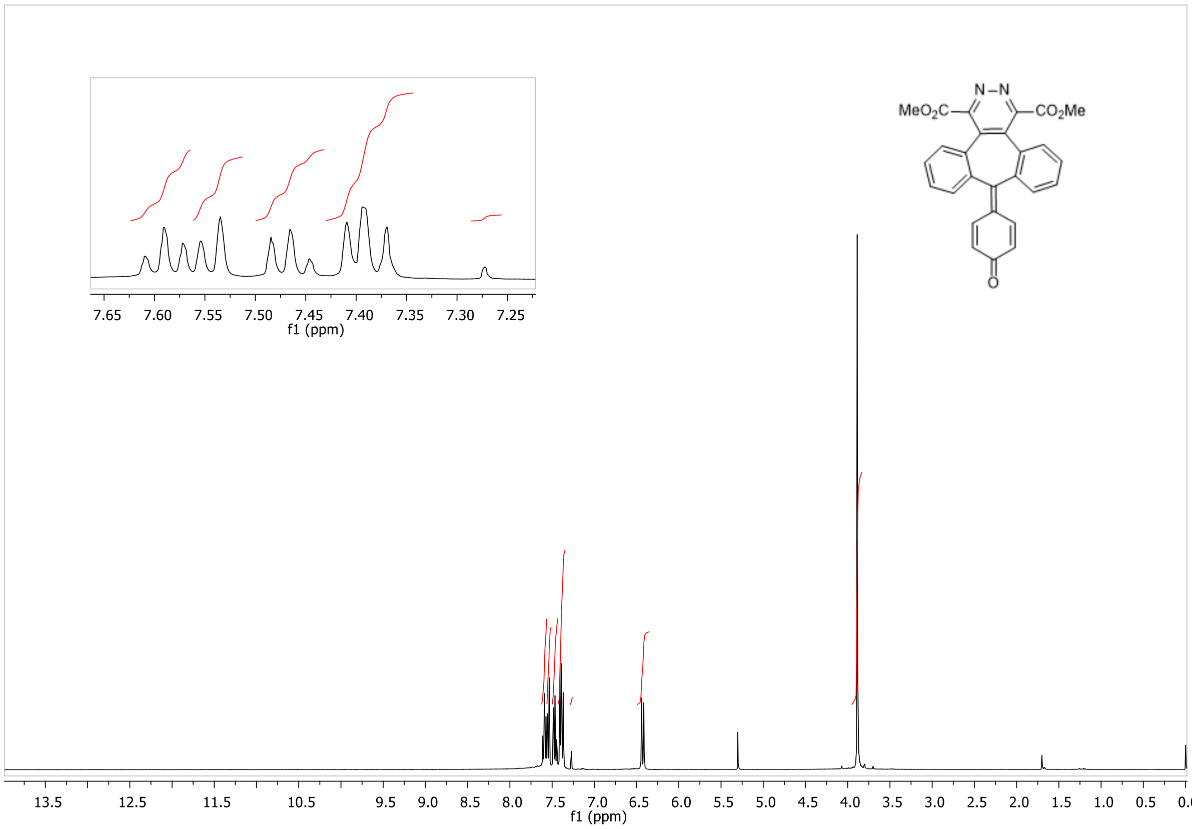

Fig. S49. ^{1}H-NMR spectrum of **14a** (400 MHz, $CDCl_3$).

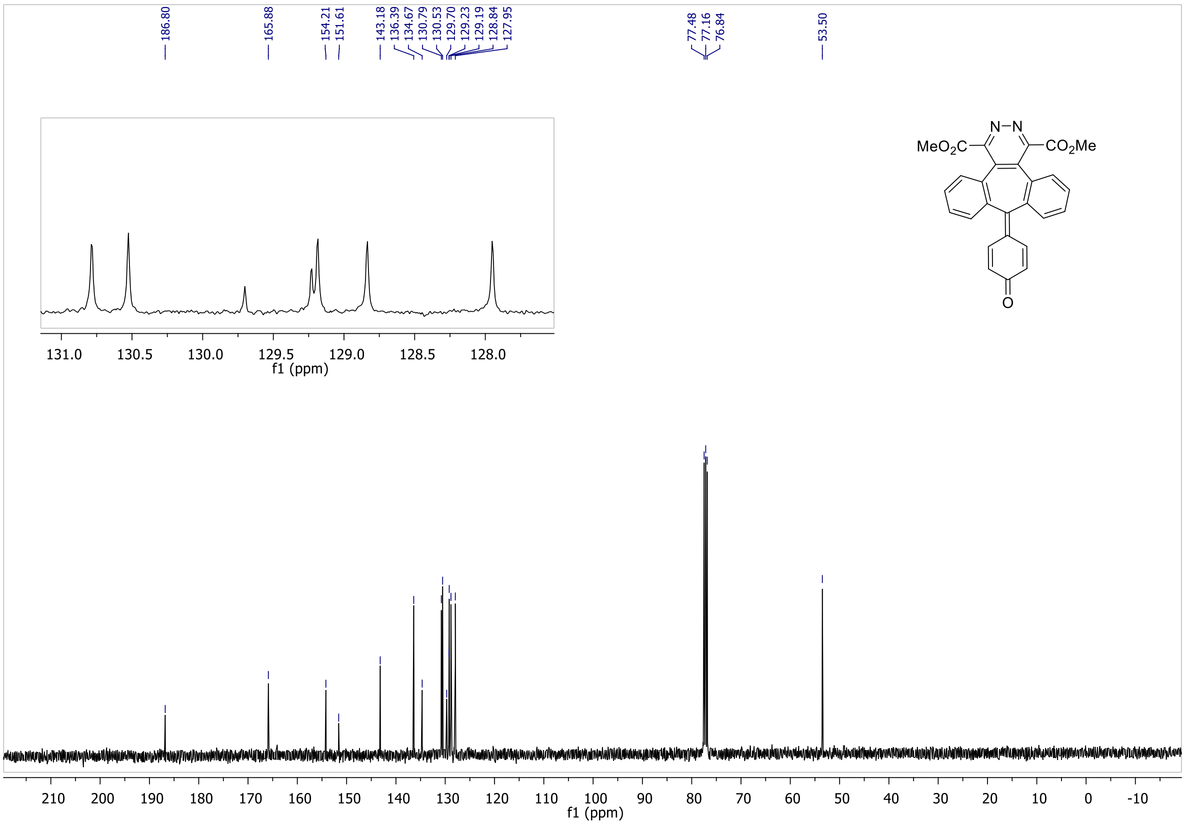

Fig. S50. ^{13}C-NMR spectrum of **14a** (100 MHz, CDCl3).

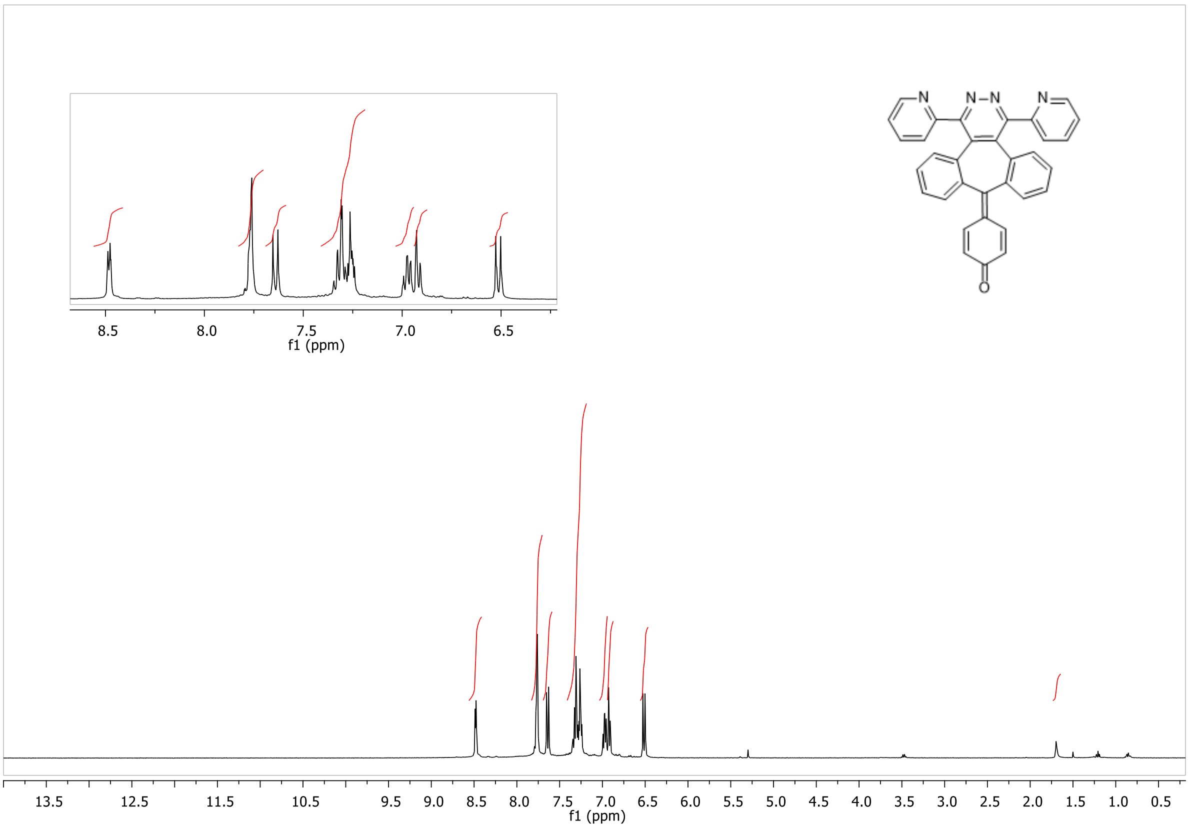

Fig. S51. ^{1}H-NMR spectrum of **14b** (400 MHz, CDCl3).

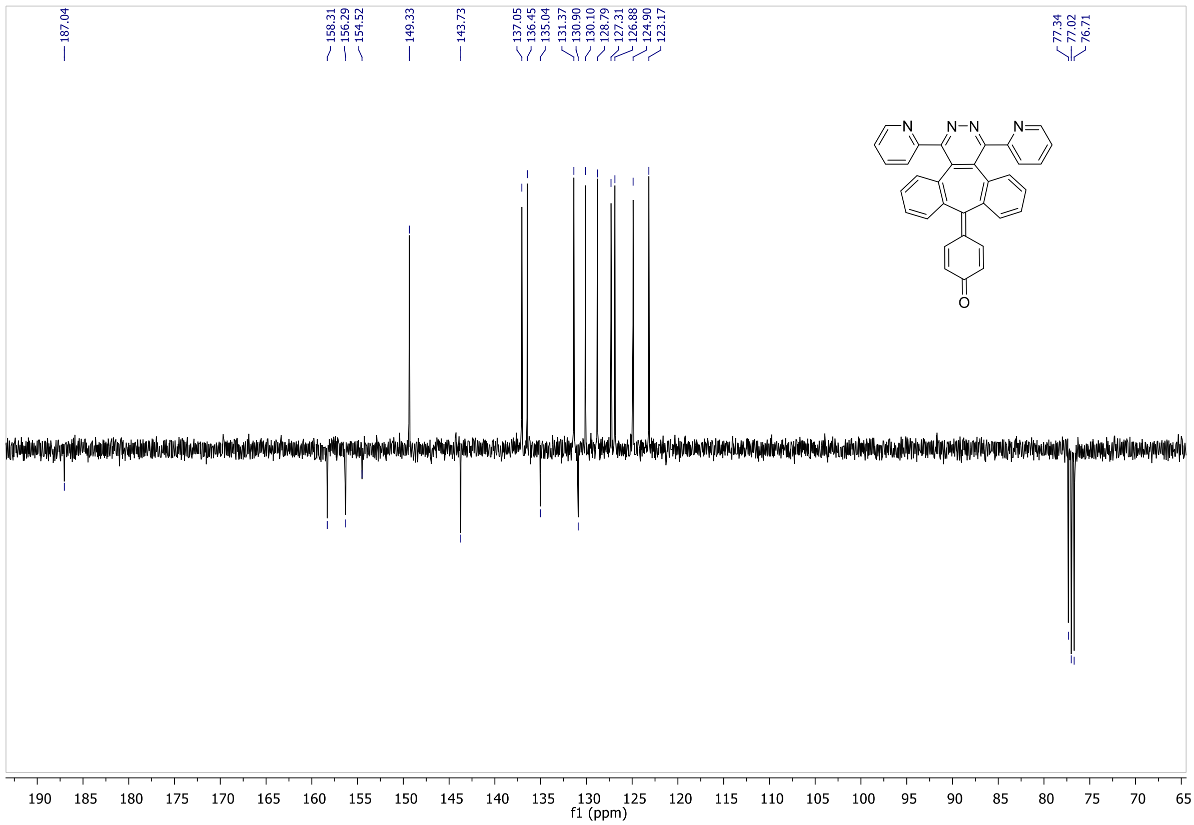

Fig. S52. ^{13}C-NMR spectrum of **14b** (100 MHz, $CDCl_3$).

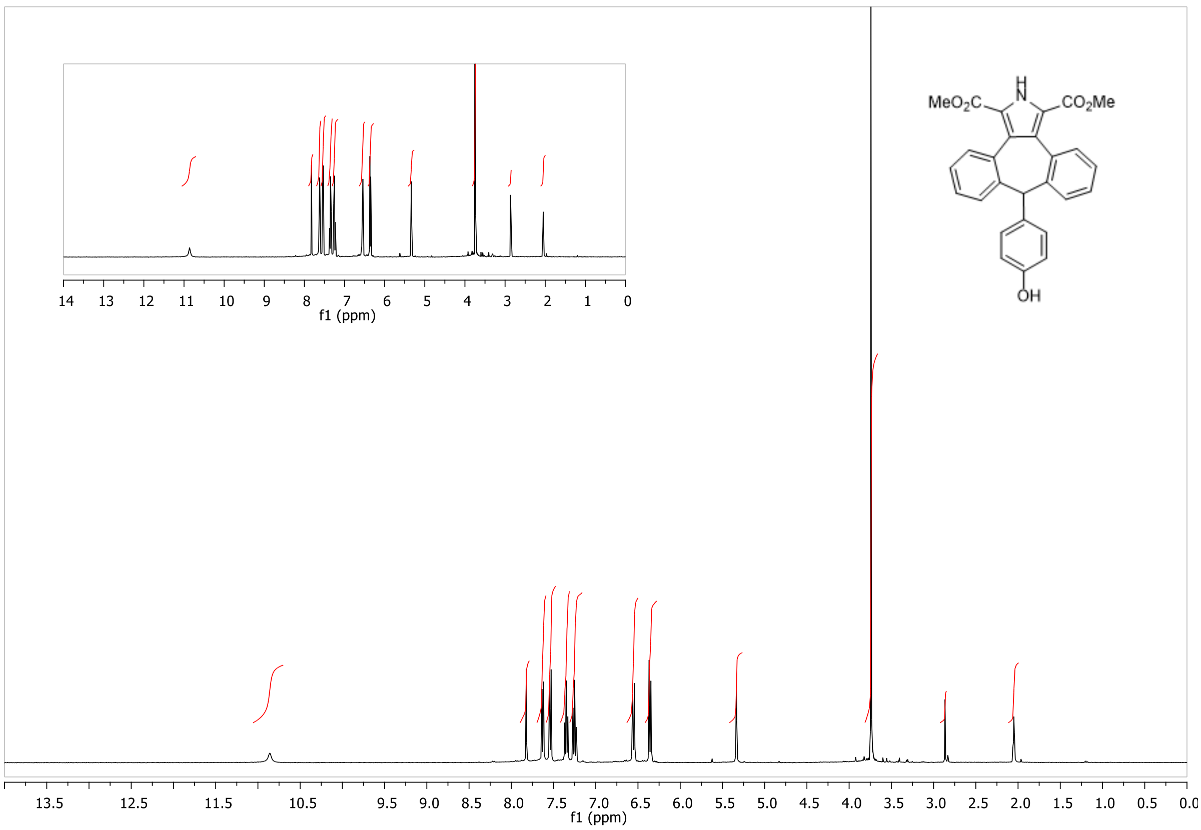

Fig. S53. ^{1}H-NMR spectrum of **15a** (400 MHz, aceton-d_6).

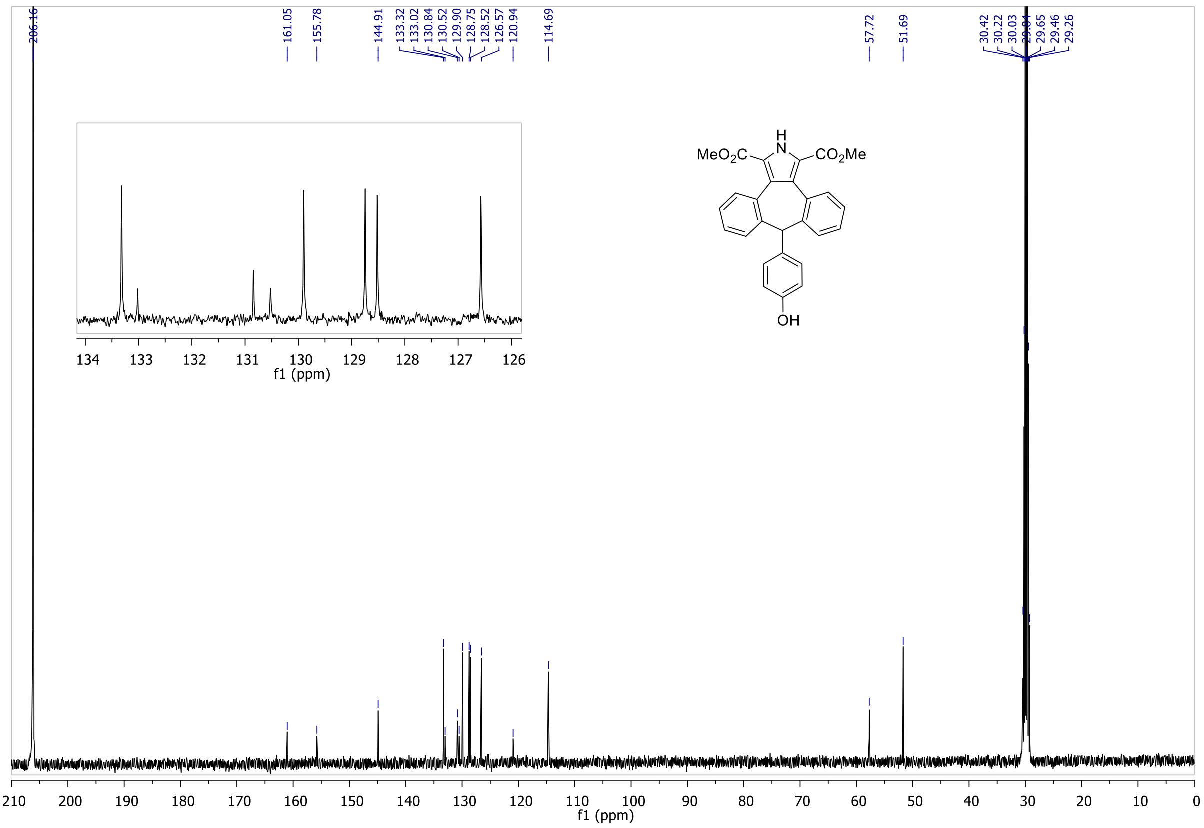

Fig. S54. ^{13}C-NMR spectrum of **15a** (100 MHz, aceton-d_6).

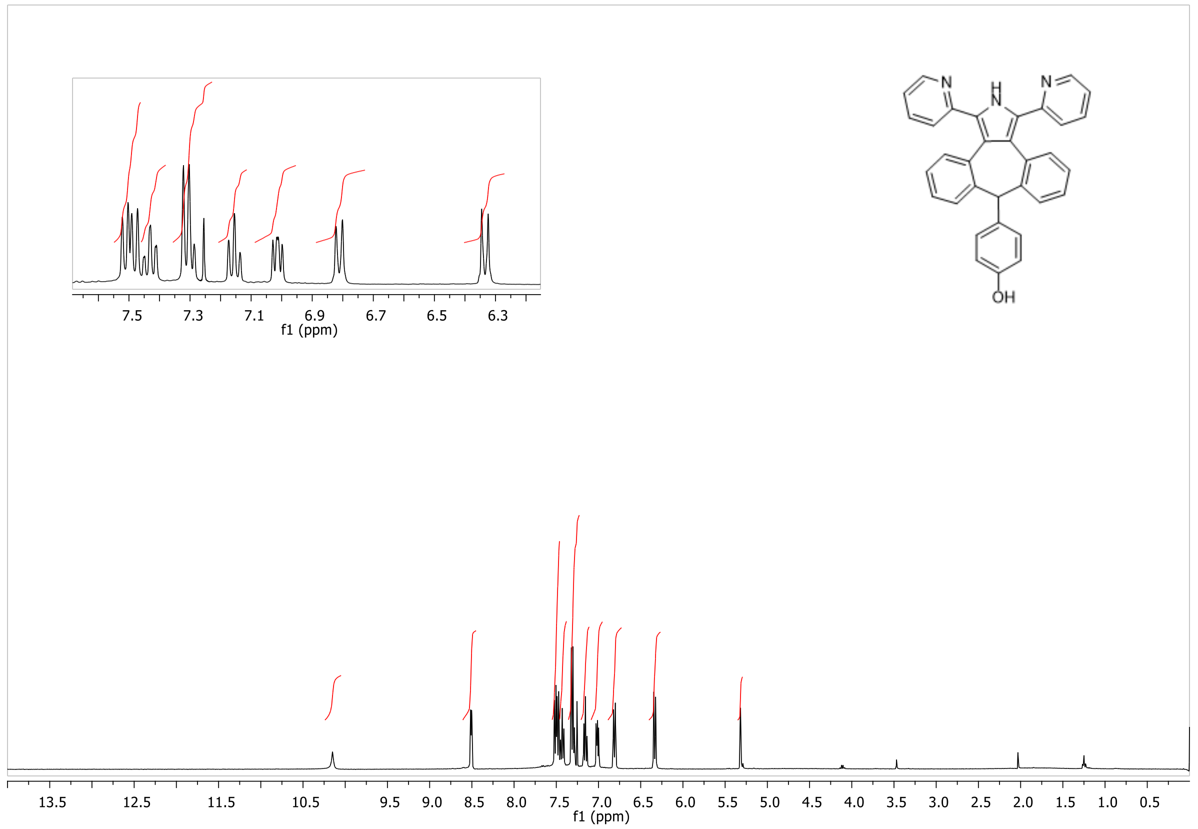

Fig. S55. ^{1}H-NMR spectrum of **15b** (400 MHz, $CDCl_3$).

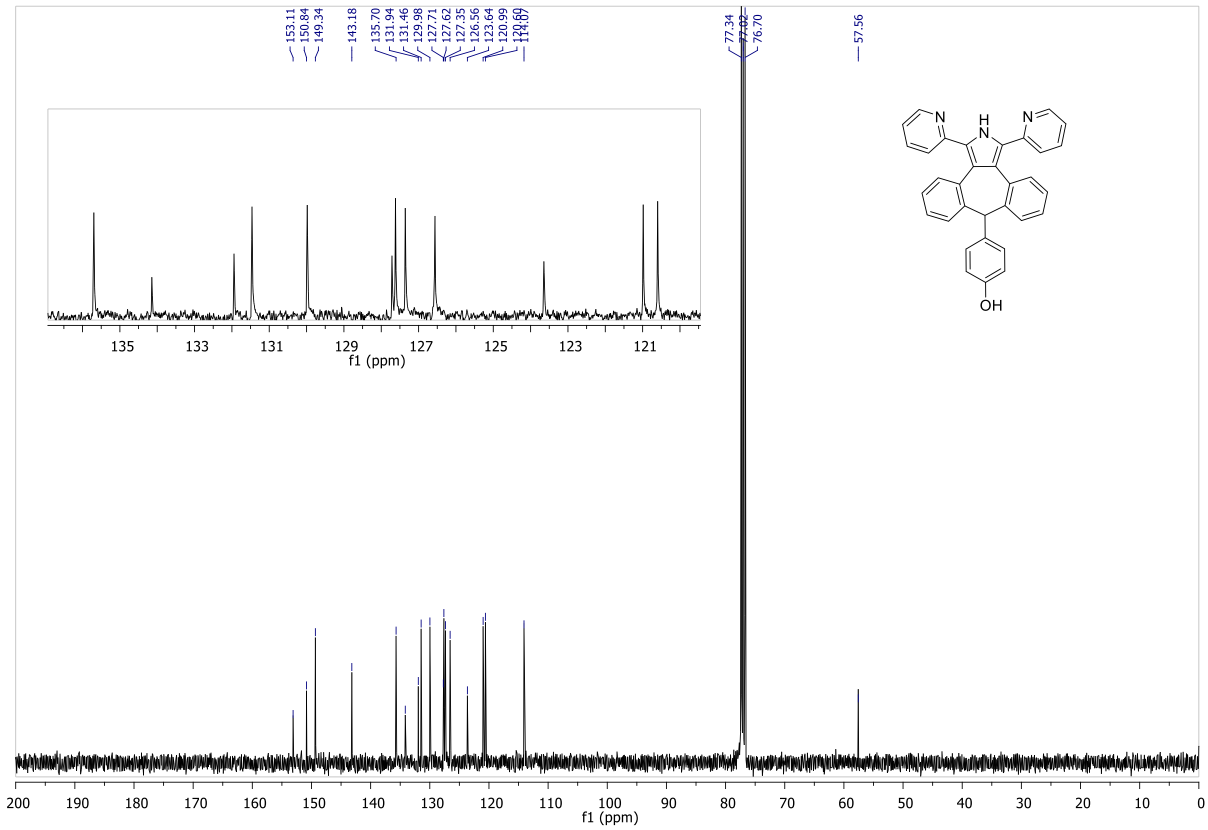

Fig. S56. ^{13}C-NMR spectrum of **15b** (100 MHz, $CDCl_3$).

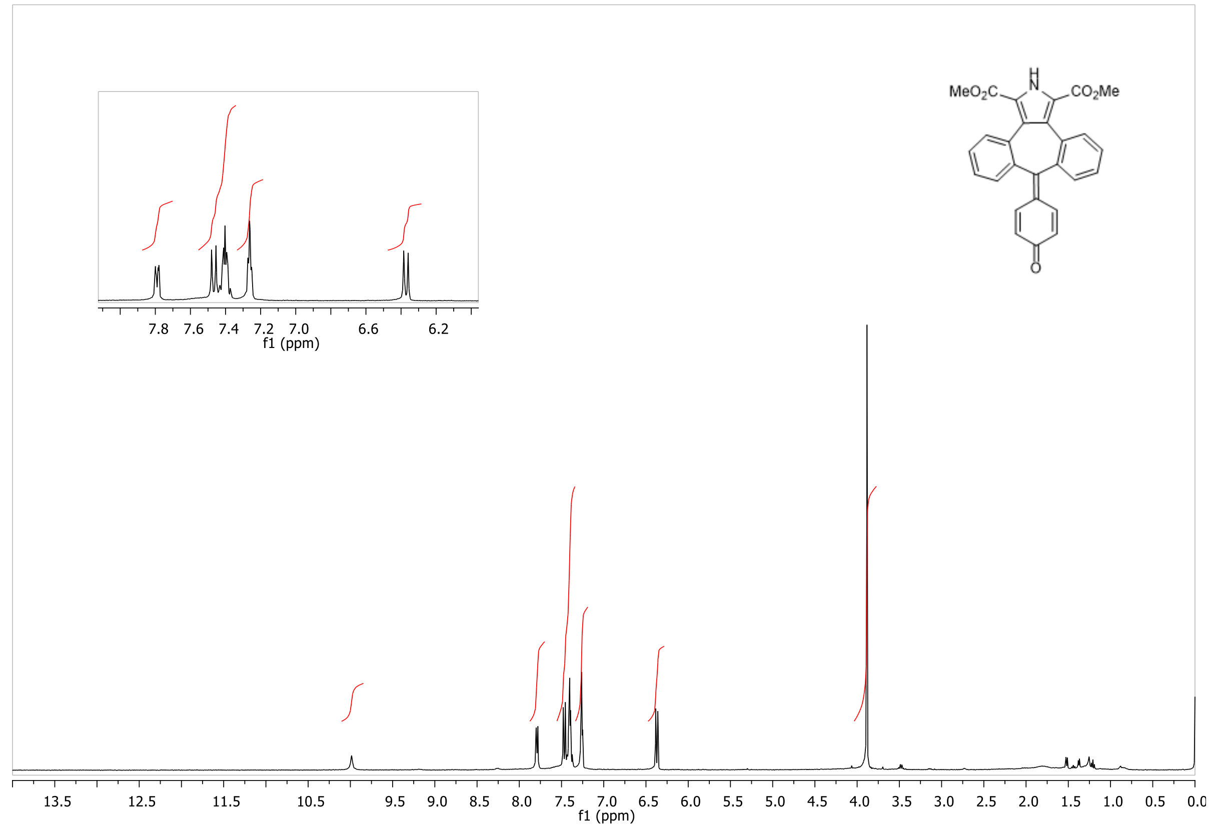

Fig. S57. ^{1}H-NMR spectrum of **16a** (400 MHz, $CDCl_3$).

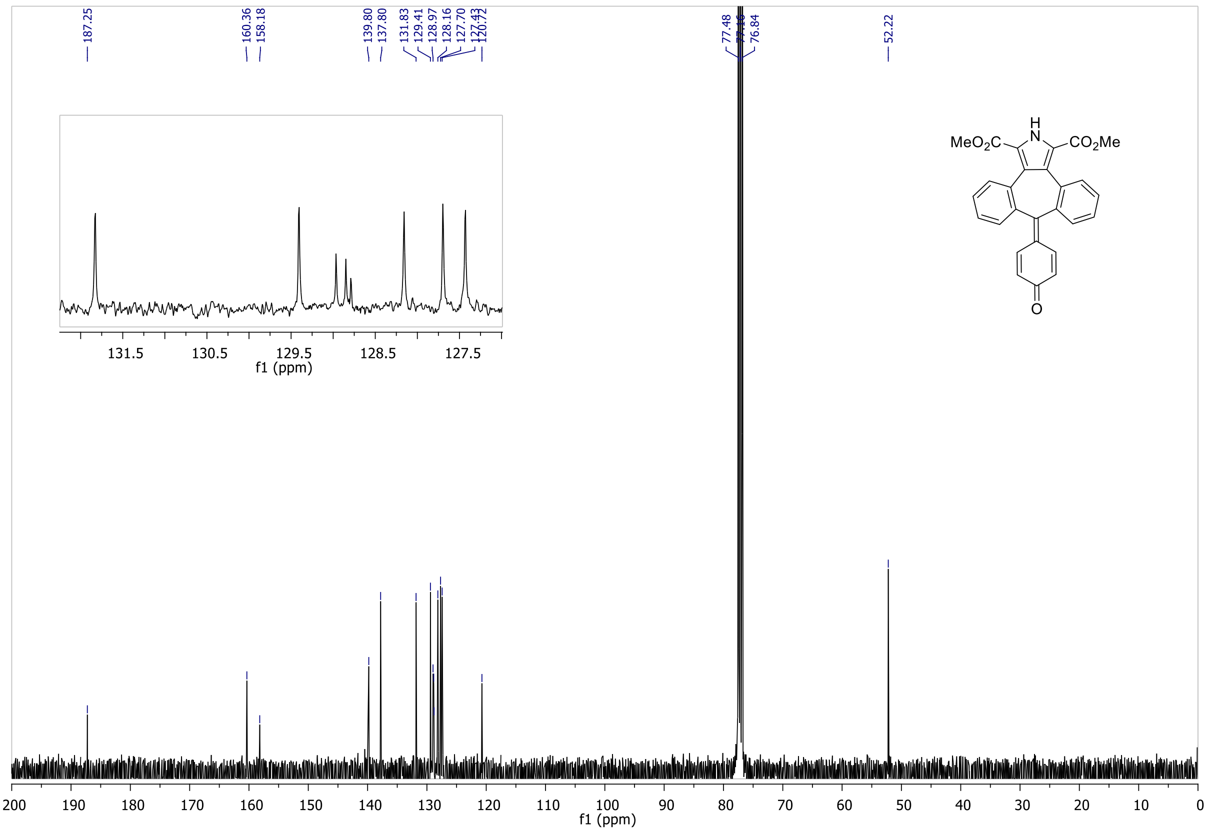

Fig. S58. 13C-NMR spectrum of **16a** (100 MHz, CDCl3).

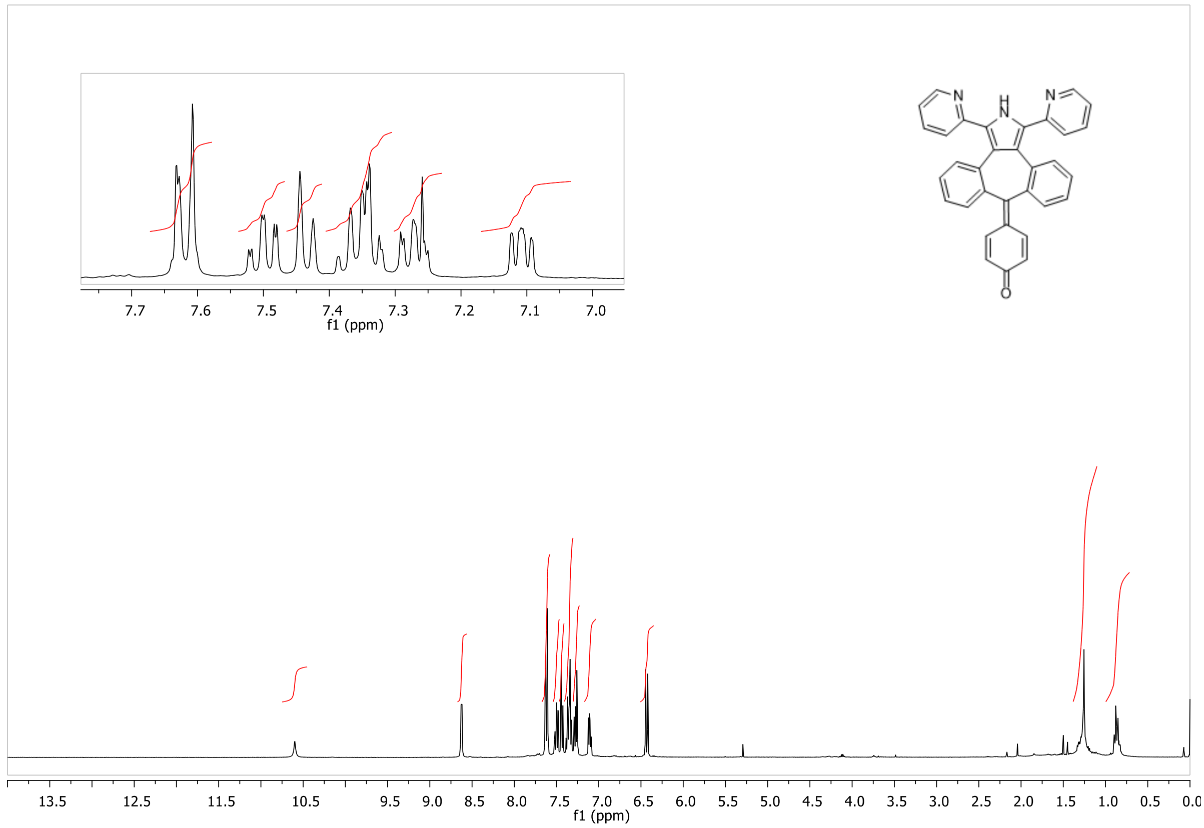

Fig. S59. 1H-NMR spectrum of **16b** (400 MHz, CDCl3).

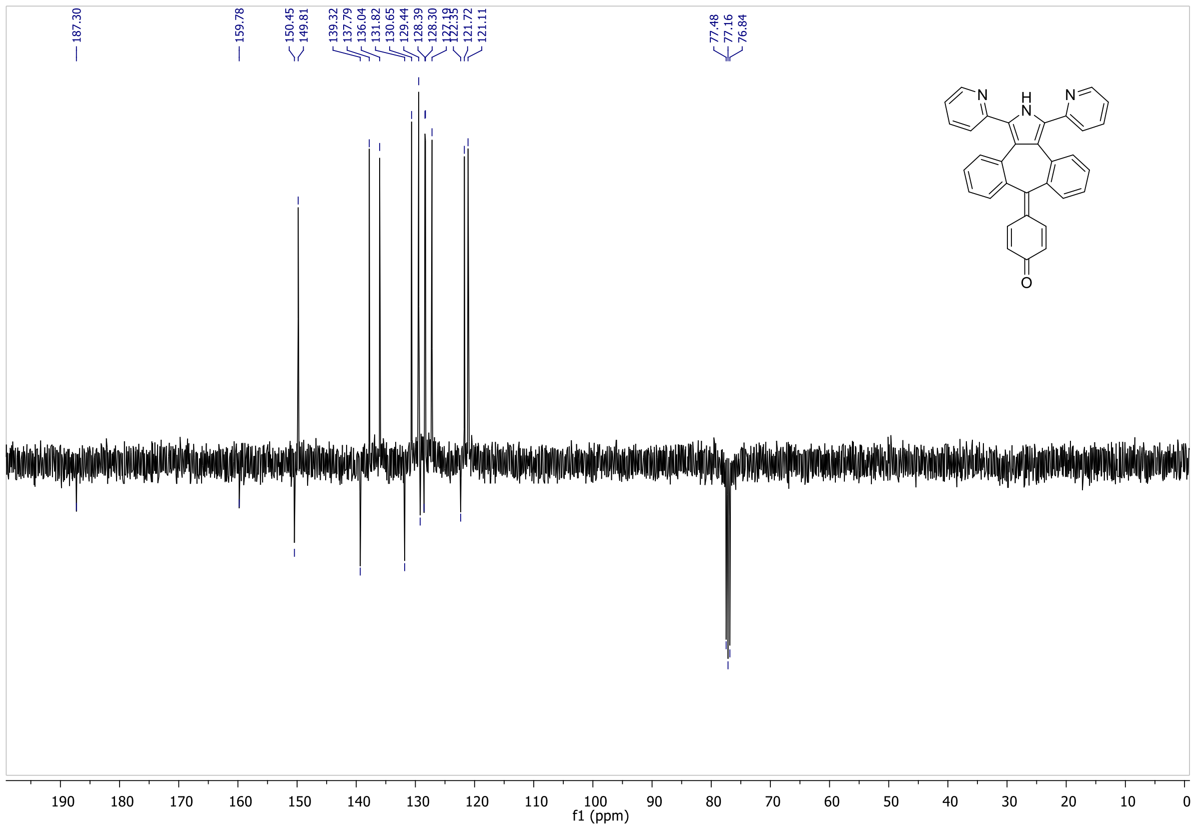

Fig. S60. ^{13}C-NMR spectrum of **16b** (100 MHz, $CDCl_3$).

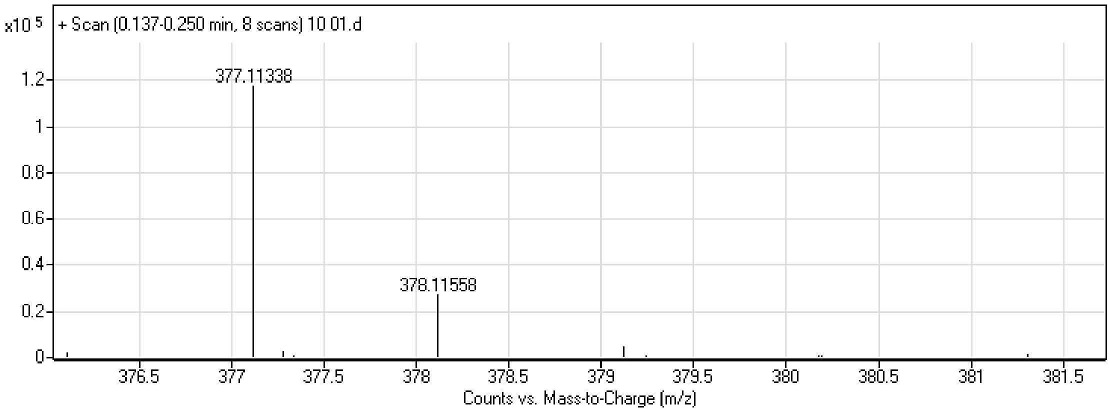

Fig. S61. HRMS spectrum of **3a**.

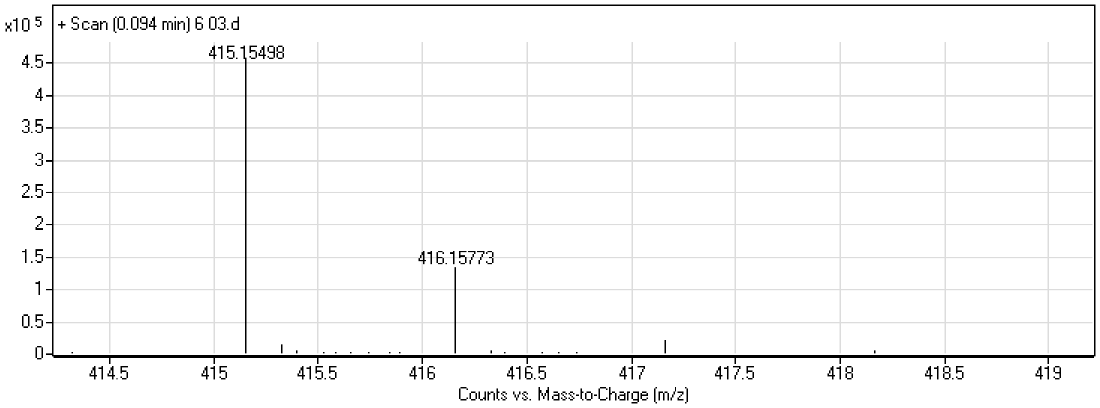

Fig. S62. HRMS spectrum of **3b**.

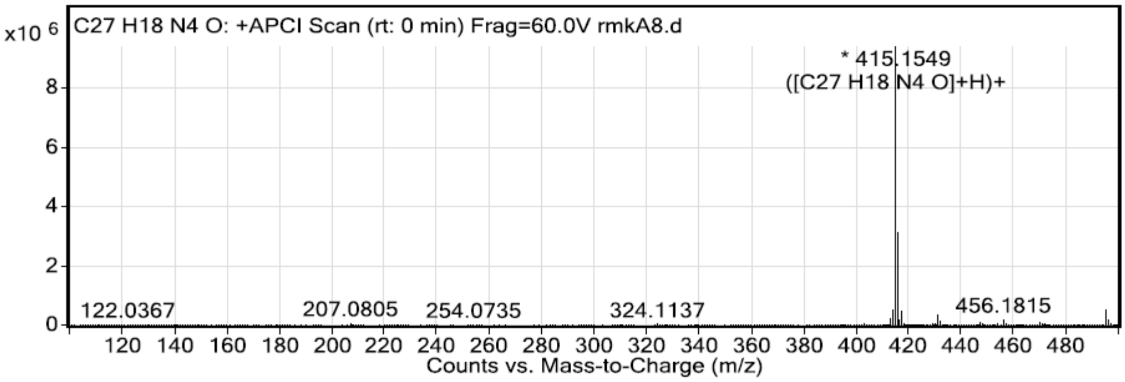

Fig. S63. HRMS spectrum of **3c**.

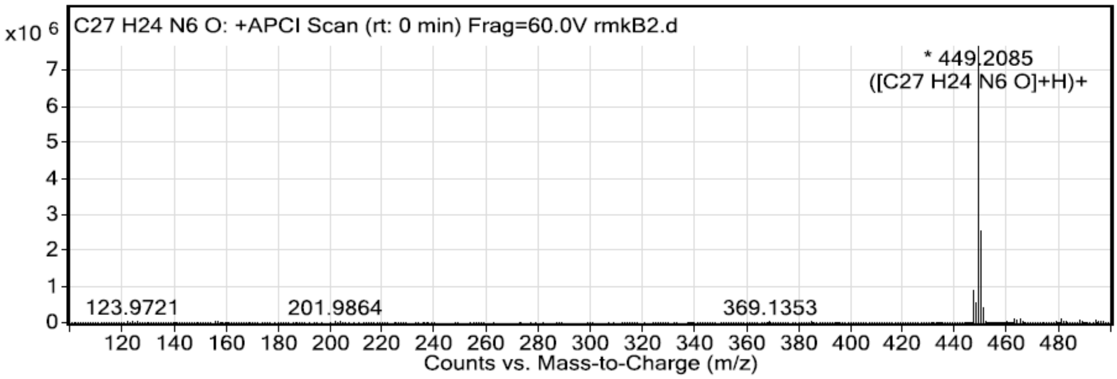

Fig. S64. HRMS spectrum of **3d**.

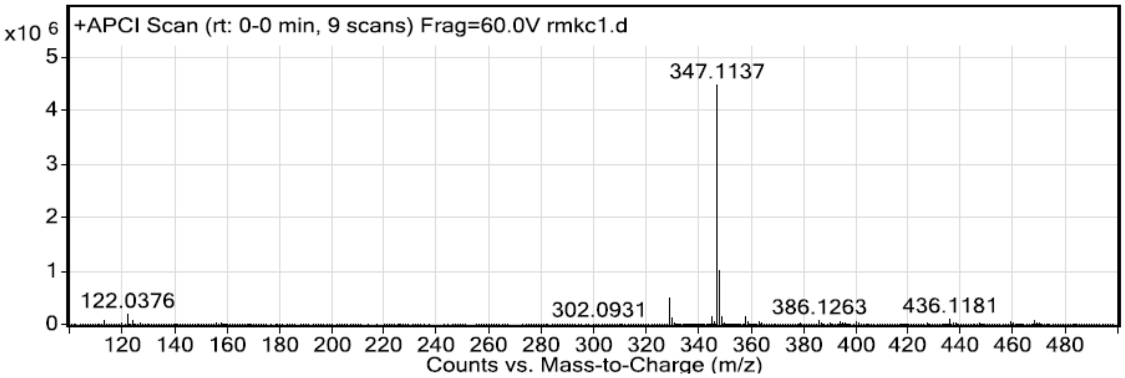

Fig. S65. HRMS spectrum of **3e**.

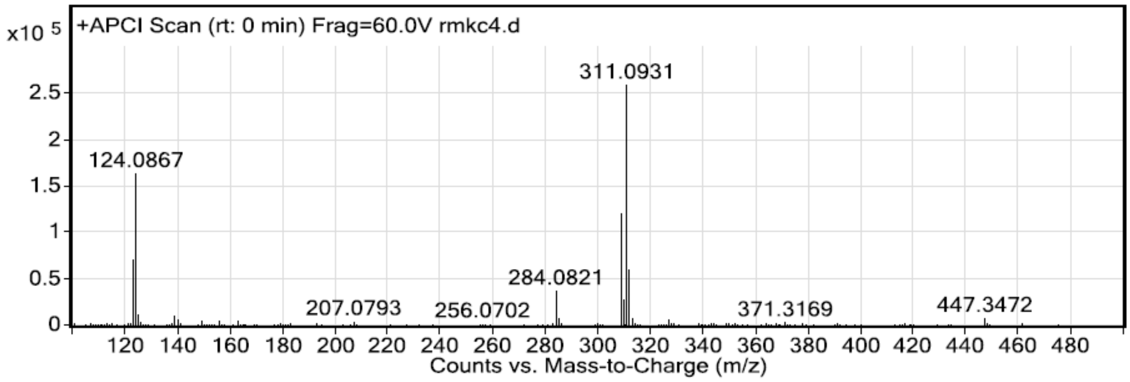

Fig. S66. HRMS spectrum of **3f**.

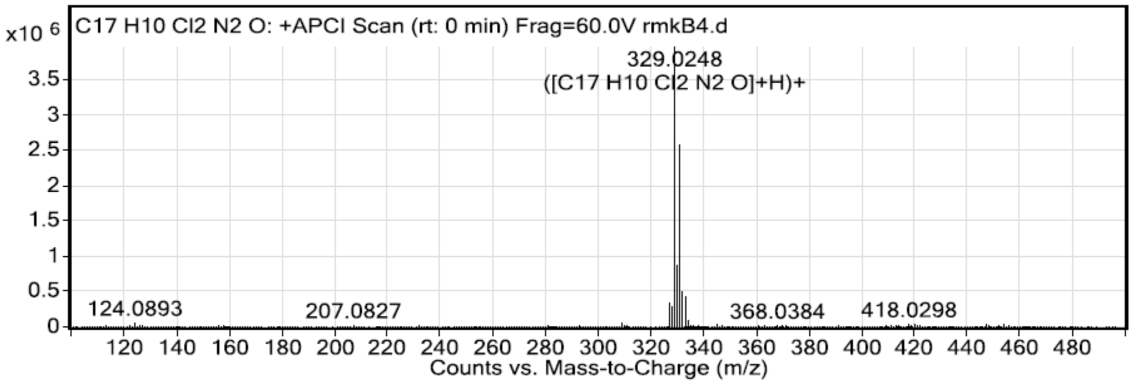

Fig. S67. HRMS spectrum of **3k**.

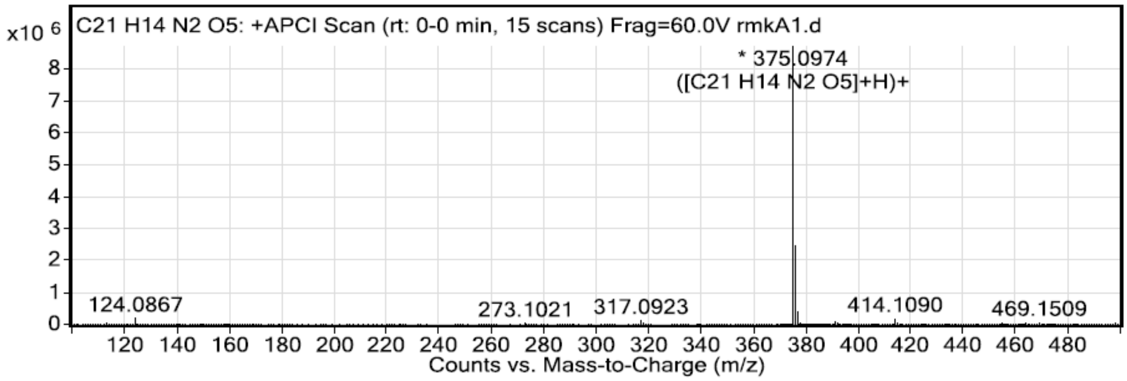

Fig. S68. HRMS spectrum of **4a**.

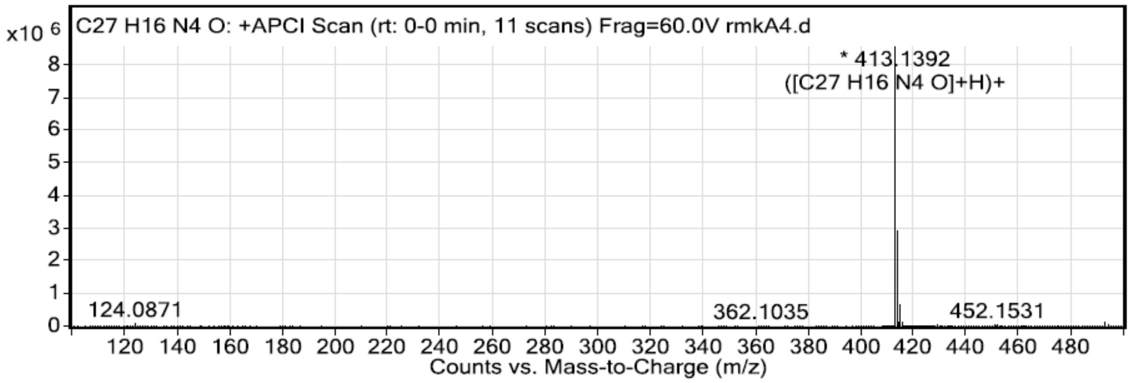

Fig. S69. HRMS spectrum of **4b**.

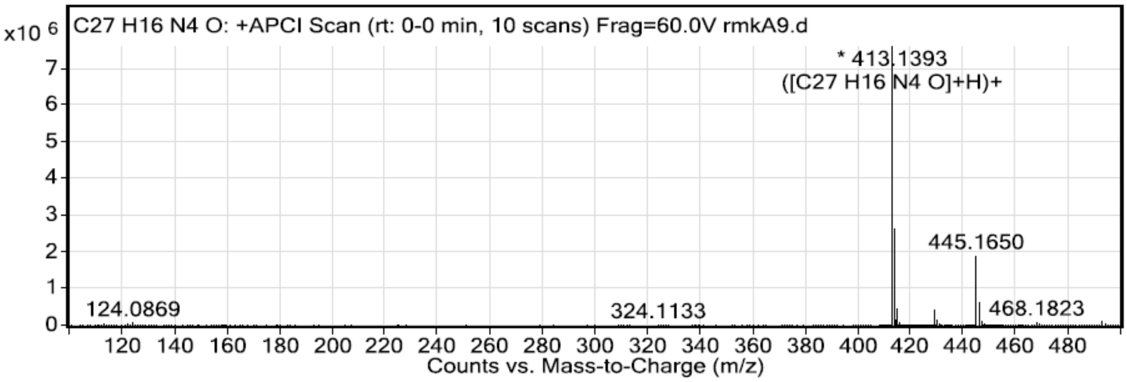

Fig. S70. HRMS spectrum of **4c**.

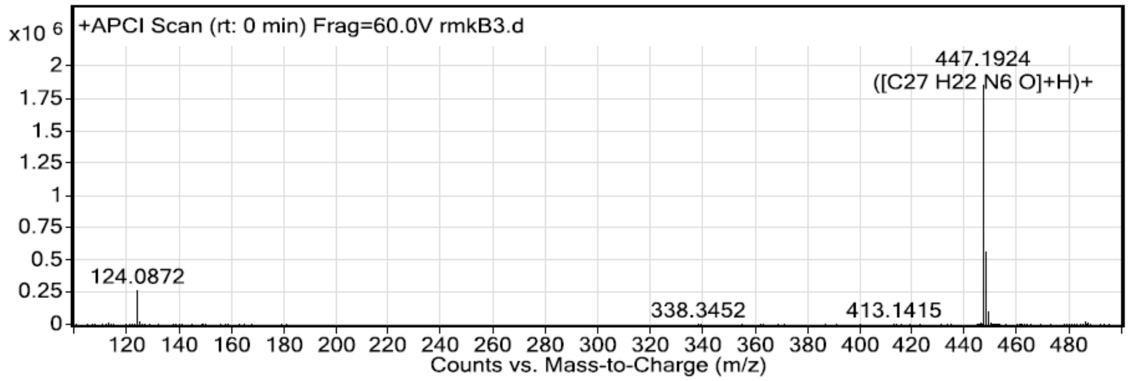

Fig. S71. HRMS spectrum of **4d**.

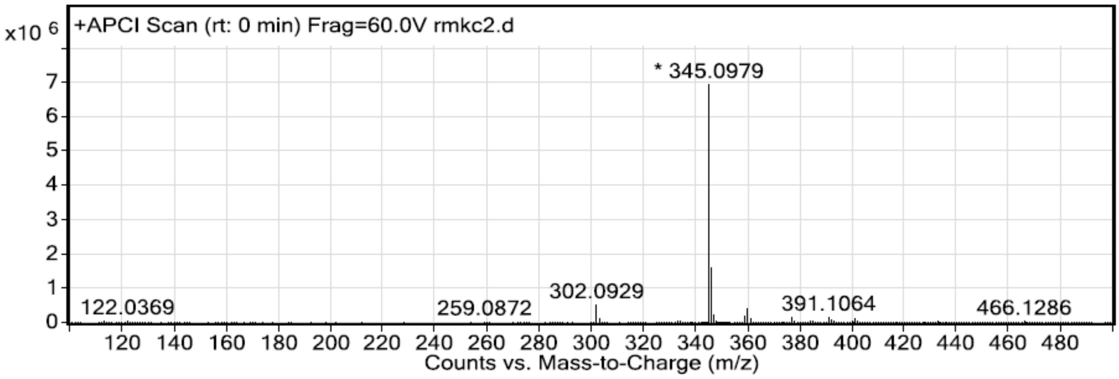

Fig. S72. HRMS spectrum of **4e**.

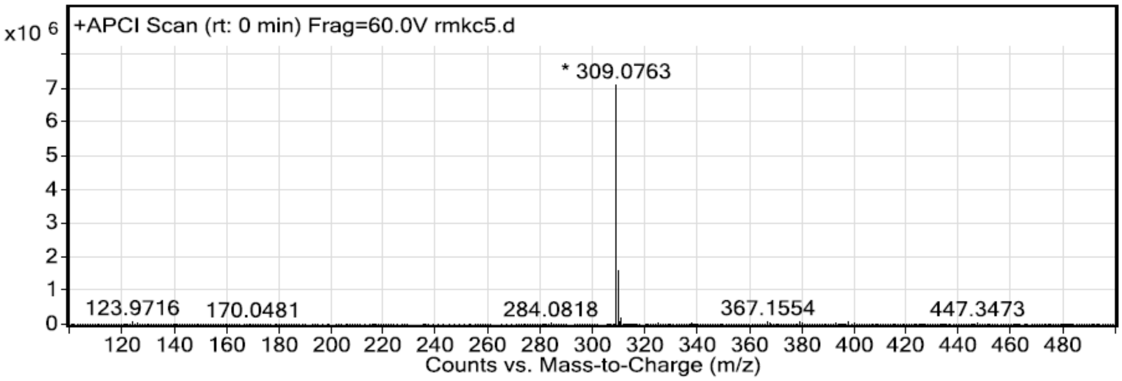

Fig. S73. HRMS spectrum of **4f**.

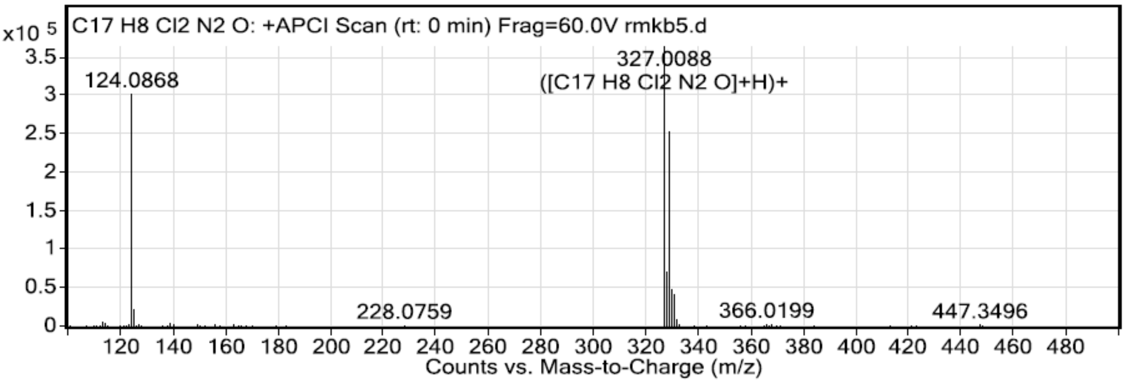

Fig. S74. HRMS spectrum of **4k**.

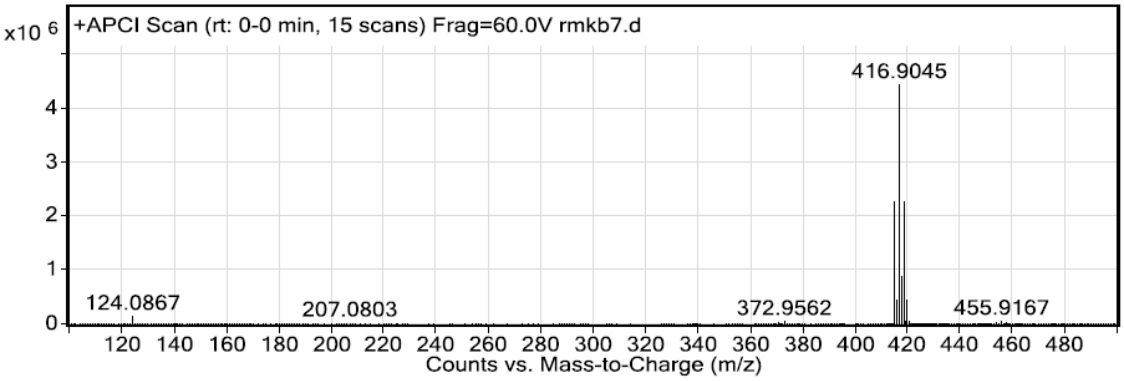

Fig. S75. HRMS spectrum of **4l**.

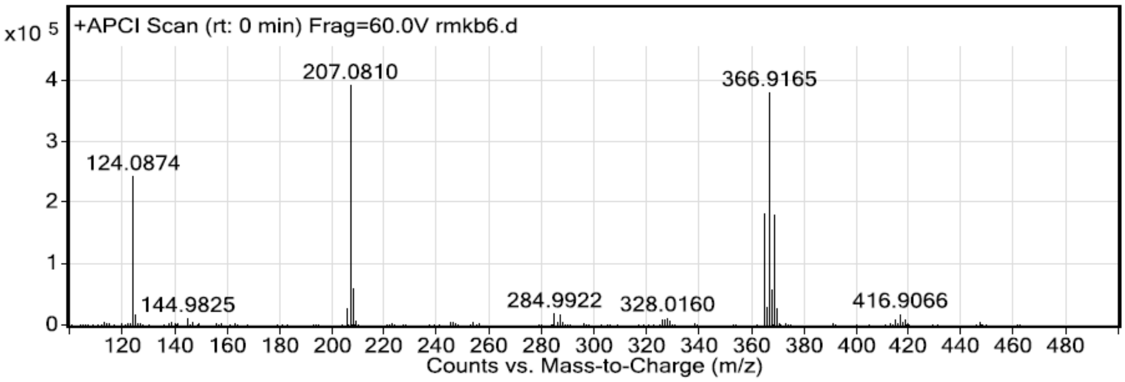

Fig. S76. HRMS spectrum of **5l**.

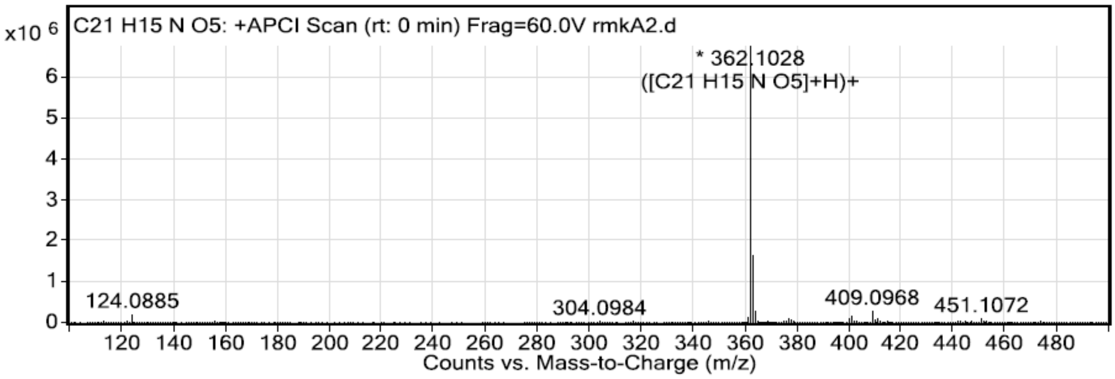

Fig. S77. HRMS spectrum of **10aa**.

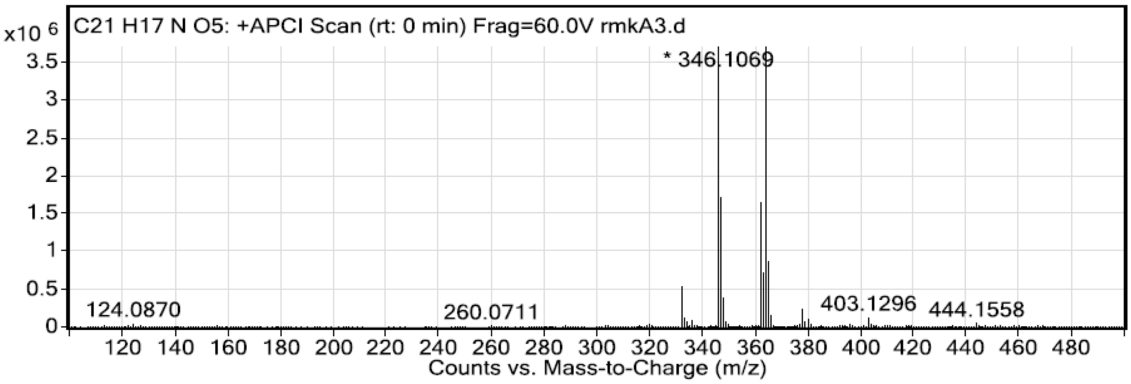

Peak List

m/z	z	Abund	Formula	Ion
332.0923	1	531838.31		
346.1069	1	6860042.5		
347.1101	1	1711142.75		
348.1179	1	377347.03		
362.1019	1	1651337.13		
363.1077	1	719684.31		
364.1171	1	3694491.75	C21 H17 N O5	(M+H)+
365.1208	1	864817.25	C21 H17 N O5	(M+H)+
366.1245	1	140218.19	C21 H17 N O5	(M+H)+

Fig. S78. HRMS spectrum of **10ab**.

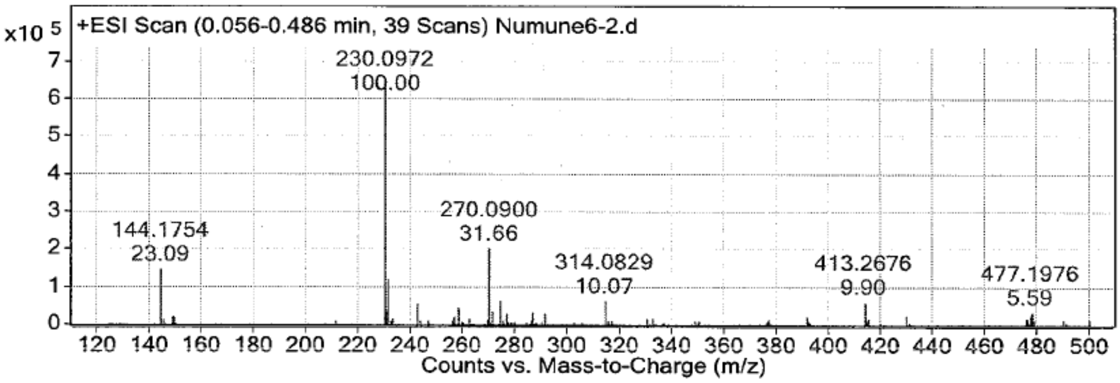

Fig. S79. HRMS spectrum of **10ac**.

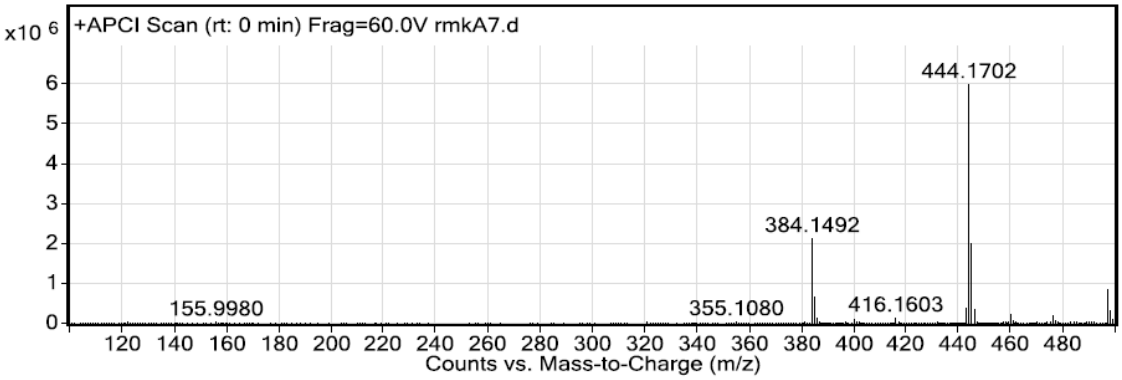

Fig. S80. HRMS spectrum of **10ba**.

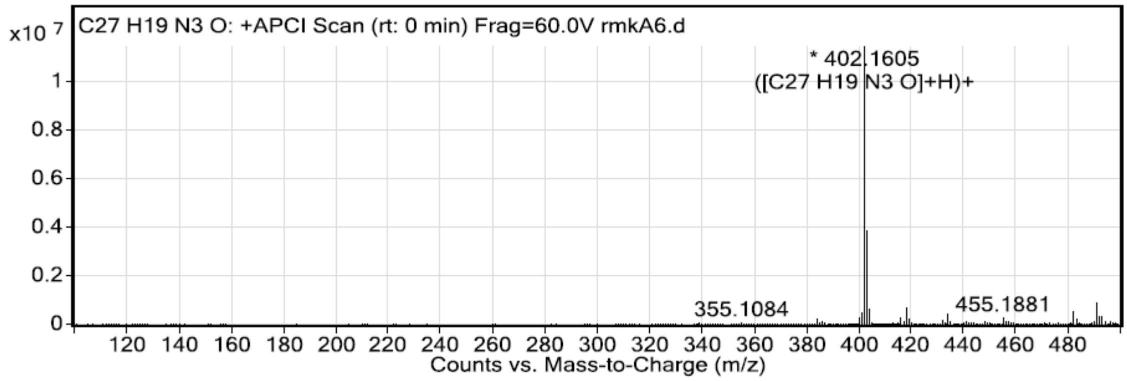

Fig. S81. HRMS spectrum of **10bb**.

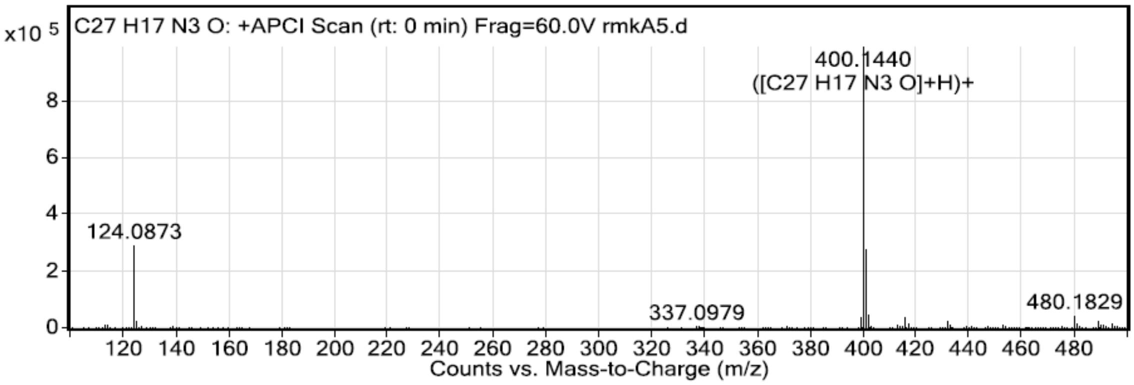

Fig. S82. HRMS spectrum of **10bc**.

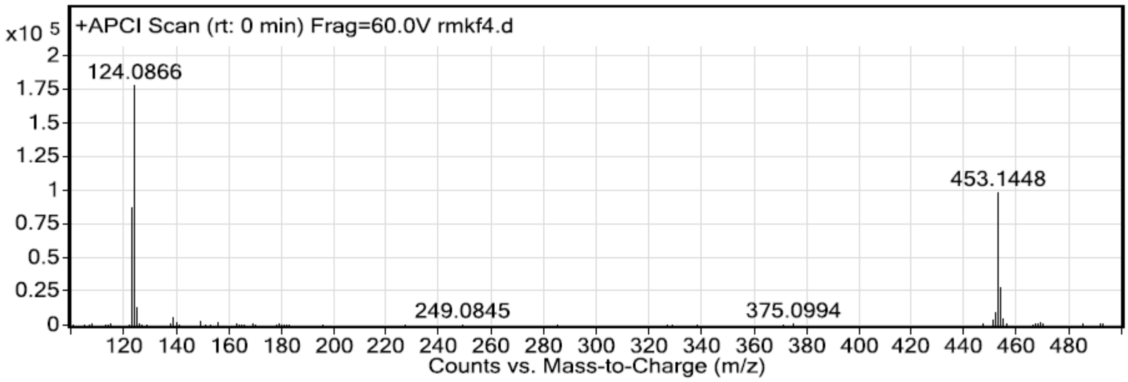

Fig. S83. HRMS spectrum of **13a**.

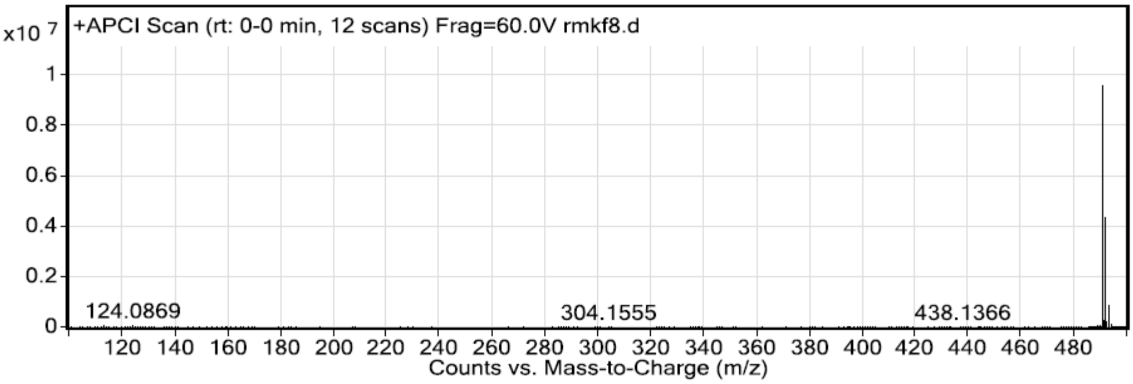

Peak List

m/z	z	Abund
491.1872	1	9567481
491.3647		254643.7
492.1893	1	4344499.5
492.3722	1	215148.05
493.1926	1	840233.56
494.1972	1	101482.65
571.2254	1	184560.77
580.1902	1	356810.16
581.1944	1	145989.55

Fig. S84. HRMS spectrum of **13b**.

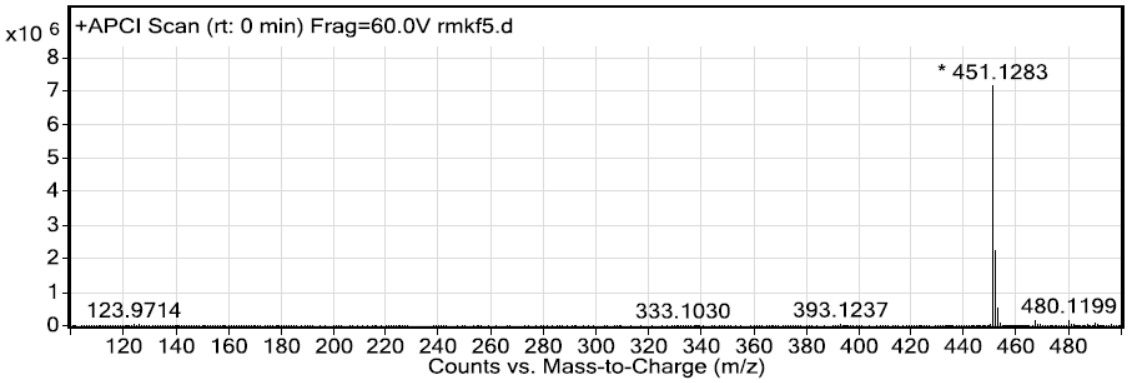

Fig. S85. HRMS spectrum of **14a**.

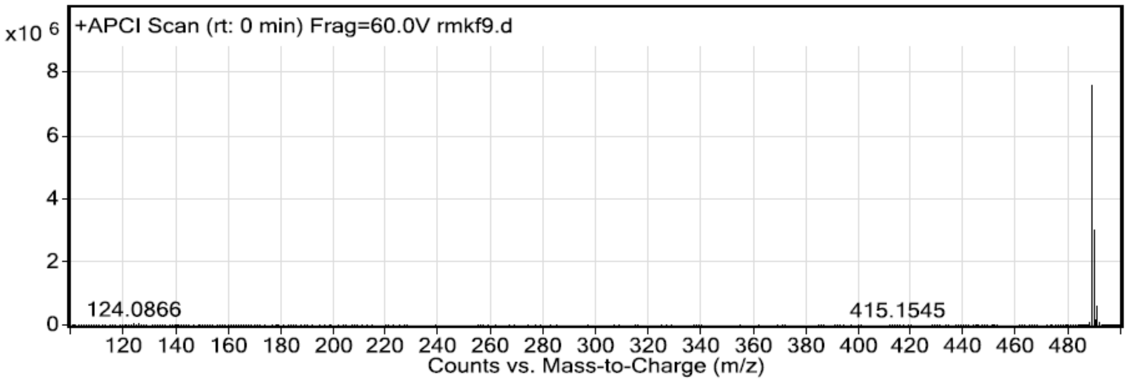

Peak List

m/z	z	Abund
489.17	1	7575265.5
490.1727	1	3012450
490.3567		160626.06
491.1771	1	591821.5
505.1662	1	218175.23
518.1615	1	268632.13
569.2088	1	178846.02
578.1732	1	910204.31
579.178	1	378927.06

Fig. S86. HRMS spectrum of **14b**.

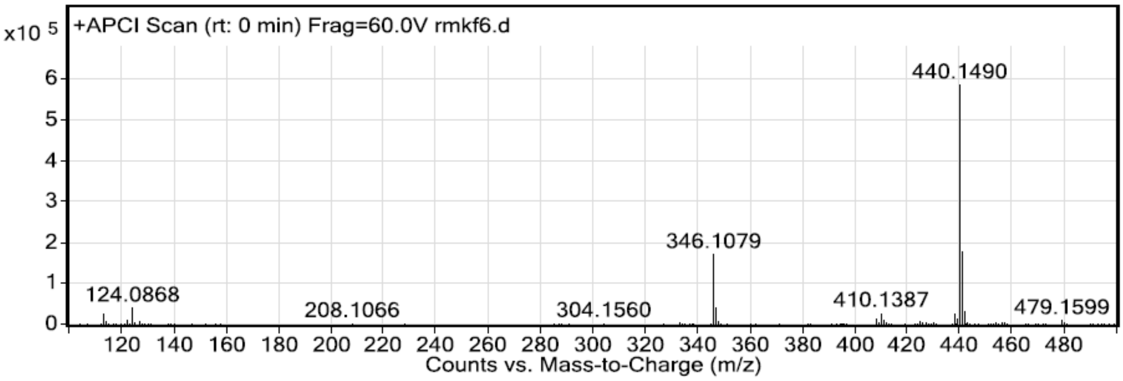

Fig. S87. HRMS spectrum of **15a**.

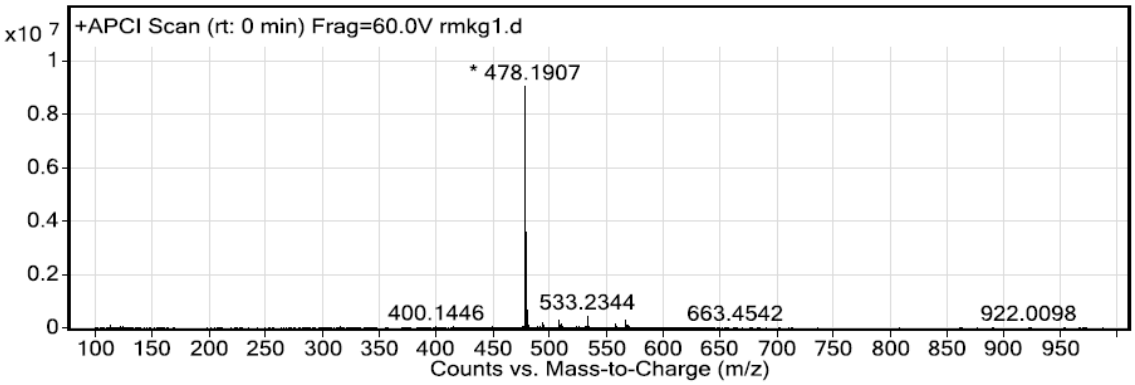

Fig. S88. HRMS spectrum of **15b**.

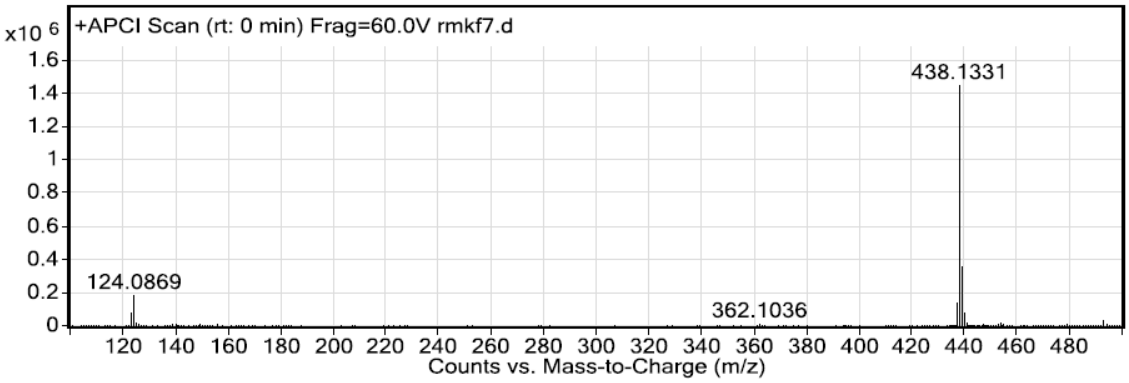

Fig. S89. HRMS spectrum of **16a**.

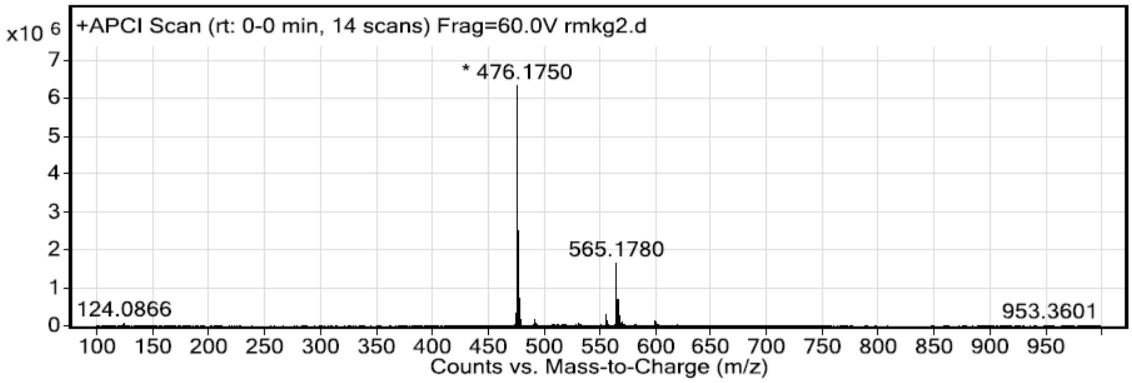

Fig. S90. HRMS spectrum of **16b**.
